# Genetic Variability of West Nile Virus in U.S. Blood Donors from the 2012 Epidemic Season

**DOI:** 10.1371/journal.pntd.0004717

**Published:** 2016-05-16

**Authors:** Andriyan Grinev, Caren Chancey, Evgeniya Volkova, Germán Añez, Daniel A. R. Heisey, Valerie Winkelman, Gregory A. Foster, Phillip Williamson, Susan L. Stramer, Maria Rios

**Affiliations:** 1 Laboratory of Emerging Pathogens (LEP), Division of Emerging and Transfusion Transmitted Diseases (DETTD), Office of Blood Research and Review (OBRR), Center for Biologics Evaluation and Research (CBER), Food and Drug Administration (FDA), Silver Spring, Maryland, United States of America; 2 Creative Testing Solutions, Tempe, Arizona, United States of America; 3 American Red Cross, Gaithersburg, Maryland, United States of America; Florida Gulf Coast University, UNITED STATES

## Abstract

West Nile virus (WNV) is an arbovirus maintained in nature in a bird-mosquito enzootic cycle which can also infect other vertebrates including humans. WNV is now endemic in the United States (U.S.), causing yearly outbreaks that have resulted in an estimated total of 4–5 million human infections. Over 41,700 cases of West Nile disease, including 18,810 neuroinvasive cases and 1,765 deaths, were reported to the CDC between 1999 and 2014. In 2012, the second largest West Nile outbreak in the U.S. was reported, which caused 5,674 cases and 286 deaths. WNV continues to evolve, and three major WNV lineage I genotypes (NY99, WN02, and SW/WN03) have been described in the U.S. since introduction of the virus in 1999. We report here the WNV sequences obtained from 19 human samples acquired during the 2012 U.S. outbreak and our examination of the evolutionary dynamics in WNV isolates sequenced from 1999–2012. Maximum-likelihood and Bayesian methods were used to perform the phylogenetic analyses. Selection pressure analyses were performed with the HyPhy package using the Datamonkey web-server. Using different codon-based and branch-site selection models, we detected a number of codons subjected to positive pressure in WNV genes. Thirteen of the 19 completely sequenced isolates from 10 U.S. states were genetically similar, sharing up to 55 nucleotide mutations and 4 amino acid substitutions when compared with the prototype isolate WN-NY99. Overall, these analyses showed that following a brief contraction in 2008–2009, WNV genetic divergence in the U.S. continued to increase in 2012, and that closely related variants were found across a broad geographic range of the U.S., coincident with the second-largest WNV outbreak in U.S. history.

## Author Summary

West Nile virus (WNV; family *Flaviviridae*, genus *Flavivirus*) is a mosquito-borne virus maintained in a bird-mosquito enzootic cycle. WNV can occasionally infect other animals and humans, which are considered dead-end hosts because they produce too little virus in blood to re-infect mosquitoes. Most human infections (~80%) do not cause symptoms, and when symptoms do occur, they may vary from mild flu-like illness to fatal neuroinvasive disease (~1%). WNV can be transmitted by transfusion of blood and blood components and by organ transplantation, posing a risk to the blood supply and public health. There is no specific therapy or vaccine for WNV in humans. WNV now is one of the most widely distributed flaviviruses in the world. Comparative studies of WNV genetic sequences have described two major groupings of WNV, lineages I and II, and up to five newer lineages, which correlate well with the geographical point of isolation. Since 1999, WNV has spread from New York City throughout the U.S. and the Americas including Mexico, Canada, the Caribbean and South America. The emergence of WNV in the U.S. with annual outbreaks represents a unique opportunity to understand how a mosquito-borne virus adapts and evolves in a new environment. Viral adaptation to domestic mosquitoes and birds is considered to have played an important role in the spread of WNV in the U.S. Continuous surveillance of WNV genetic variation is needed to protect public health because the tests used to diagnose infection and screen blood, as well as vaccines and drug therapies currently in development, may not perform as well against newer genetic variants of WNV.

## Introduction

West Nile virus (WNV) emerged in the United States in 1999 and has become endemic, having caused annual outbreaks each subsequent year. WNV is a *Flavivirus* maintained in nature in an enzootic cycle between birds and mosquitoes. Other vertebrate hosts may be infected and develop disease, as occurs with humans and horses, which are considered dead-end hosts since they do not develop sufficient viremia to re-infect mosquitoes [1, 2]. Transmission may also occur between humans via blood transfusion and transplantation of organs from infected individuals [3,4]. Since 2003, donated blood has been routinely screened for WNV by nucleic acid testing (NAT), and thousands of transmissions have been prevented [5]. Approximately 80% of humans infected with WNV develop no symptoms. Symptoms of WNV infections may vary from fever, rash and flu-like symptoms to severe neurological disease, which develops in less than 1% of cases and can result in death [6–8]. According to the U.S. Centers for Disease Control and Prevention (CDC), WNV poses an ongoing public health threat, having infected millions of people and caused 1,765 deaths in the U.S. through the end of 2014 [9].

WNV is the most widely geographically distributed *Flavivirus* in the world, present on every continent except Antarctica. WNV infection had been observed in Africa, Asia, Australia/Oceania, and southern Europe prior to 1999. In 1999, the first cases of WNV in the Americas were observed in the U.S. in New York City, and the virus has since spread westward across the 48 contiguous states and Canada, and southward into Mexico, the Caribbean islands, Central America and South America, where it has caused human disease as far south as Argentina [10–12].

In the U.S., WNV causes annual outbreaks of varying size and severity. Peaks of WNV activity have been observed in 2002–2003, 2006 and 2012. Reduced WNV activity was observed from 2008–2011 compared to 2002–2007 [9]. Following this period of relatively low activity, a large outbreak of WNV disease occurred in the 48 contiguous states in 2012 with 5,674 reported cases including 2,873 neuroinvasive cases and 286 deaths, the largest numbers reported to the ArboNET for any year since 2003. [9]. WNV disease cases peaked in late August 2012, with 5,199 (92%) cases having onset of illness during July—September. The incidence of WNV neuroinvasive disease increased in 2012 to 0.92 per 100,000. More than half of the neuroinvasive disease cases in 2012 were reported from four states: Texas (n = 844), California (n = 297), Illinois (n = 187), and Louisiana (= 155) [9, 14]. There are an estimated 30–70 non-neuroinvasive disease cases for every reported case of WNV neuroinvasive disease [6, 8, 13]. Therefore, an estimated 86,000–200,000 non-neuroinvasive disease cases might have occurred in 2012 but only 2,801 were diagnosed and reported. [14]. The reason for the increased incidence of WNV disease in 2012 is unknown and may involve multiple environmental and ecological factors as well as selection and dissemination of genetically best-fitted viruses.

The spread of WNV in the Americas has offered a unique opportunity to observe evolution and genetic adaptation occurring in an arbovirus introduced to a new environment. The prototype strain from the 1999 New York outbreaks became known as the NY99 genotype, and is believed to share a common genetic origin with a 1998 Israeli isolate IS-98 [11, 15]. In 2002, a new WNV genotype, WN02, appeared and was characterized by one amino acid substitution, E-V_159_A, and 13 conserved nucleotide mutations [16, 17]. WN02 was found to be more efficiently transmitted by New World mosquitoes than NY99, and eventually completely replaced NY99 [18]. This genetic shift coincided in time with large U.S. outbreaks in 2002–2003 and may have contributed to WNV’s spread across North America. Even with the genetic changes observed as WNV spread through North America, genetic variability of human isolates remained relatively low, increasing from 0.18% in 2002 to 0.37% in 2005 [19]. A second new genotype termed SW/WN03, defined by two additional fixed amino acid substitutions, NS4A-A_85_T and NS5-K_314_R, was first observed in isolates collected in 2003. WN02 and SW/WN03 genotypes displaced the ancestor NY99 genotype in the U.S. [20].

High WNV activity in the U.S. continued through 2006 and 2007, and during this period, further genetic diversification of WNV strains was observed. A new well-defined viral cluster occurring within genotype SW/WN03, termed MW/WN06, was observed in strains collected from blood donors in the Midwestern and Northwestern U. S. in 2006 and 2007 [21]. The number of genetic mutations appearing in U.S. WNV strains continued to increase over this period, but the number of conserved mutations decreased slightly. Some nucleotide mutations which were previously believed to have been fixed in WNV isolates occurring after 2003 appeared to revert to the NY99 sequence, but other mutations associated with the WN02 genotype remained fixed [21]. The increased virulence of the WN02 genotype in mosquitoes is believed to have facilitated westward spread in 2002–2003 with a dramatic increase in infections, causing the largest WNV outbreak ever recognized worldwide and the largest viral encephalitis outbreak ever recognized in North America. This spread highlighted the need to monitor mutations occurring in the WNV genome and the genetic relationships of viral isolates causing disease in the U.S. over time [10–12, 17, 21].

Here we report results obtained from sequencing and phylogenetic analysis of 19 human WNV isolates from 13 U.S. states: Arizona (AZ), California (CA), Georgia (GA), Illinois (IL), Louisiana (LA), Nebraska (NE), New Mexico (NM), North Dakota (ND), Mississippi (MS), Ohio (OH), South Dakota (SD), Texas (TX), and Wyoming (WY), from blood donations collected during the 2012 epidemic season. Thirteen of the 19 completely sequenced isolates from 10 U.S. states (ND, SD, WY, TX, MS, GA, NM, OH, NE, IL) were genetically similar, sharing up to 55 nucleotide mutations and 4 amino acid substitutions when compared with WN-NY99 (GenBank accession number AF196835). Phylogenetically, these 13 isolates clustered together with previously published 2012 isolates from TX [22, 23] and some 2012 isolates from CO published in GenBank suggesting that this genetic variant was widely geographically distributed in 2012. Isolates from AZ and CA were different from these genetic variants and phylogenetically clustered within local clades.

## Methods

### 1. Ethics statement

All human specimens used in this study were obtained from blood donors who signed the blood center’s Institutional Review Board (IRB) approved informed consent. These specimens were anonymized (unlinked) before shipment. Use of these unlinked specimens has been approved as exempt by the U.S. Food and Drug Administration (FDA) IRB (Human Subjects Research—Exempt RIHSC Protocol #127B).

### 2. Samples

The study included 19 isolates obtained after cultivation of residual blood specimens from blood donors who tested reactive for WNV RNA by FDA-approved commercial nucleic acid test assays used to screen blood donations. These 19 samples were representative of 13 states of the U.S.: AZ, CA, GA, IL, LA, NE, NM, ND, MS, OH, SD, TX, and WY (Table 1).

**Table 1:** List of 2012 WNV isolates completely sequenced in this study.

#	Isolate ID	State	GenBank no.
1	BSL05	AZ	KM012170
2	BSL08	MS	KM012171
3	BSL53	TX	KM012172
4	BSL78	SD	KM012173
5	BSL80	AZ	KM012174
6	BSL85	CA	KM012175
7	BSL93	ND	KM012176
8	BSL101	WY	KM012177
9	BSL107	TX	KM012178
10	BSL116	SD	KM012179
11	BSL140	GA	KM012180
12	BSL178	LA	KM012181
13	BSL195	NM	KM012182
14	BSL221	TX	KM012183
15	ARC1	OH	KM012184
16	ARC3	NE	KM012185
17	ARC4	OH	KM012186
18	ARC6	NE	KM012187
19	ARC13	IL	KM012188

### 3. Virus isolation, RNA extraction and Reverse Transcription-Polymerase Chain Reaction (RT-PCR)

A single passage in Vero cells (ATCC # CCL-81) was performed for virus isolation from each specimen as previously described by Grinev et al. [19]; cell culture supernatants were harvested within 7 days and used for viral RNA extraction by the QIAamp Viral Mini RNA extraction kit (Qiagen, Valencia, CA) according to the manufacturer’s protocol. Reverse transcription reactions, PCR amplification and purification of amplicons were performed as described earlier [19].

### 4. DNA sequencing, assembly and alignment

Amplicons covering an entire WNV genome of each studied isolate were subjected to Sanger sequencing using the amplification primers and additional internal sequencing primers. Sequencing reactions were performed as described before [19]. Sequencing data were assembled and analyzed using the Vector NTI Advance 11.5 software package (Invitrogen). Nucleotide (nt) and deduced amino acid (aa) sequences from studied isolates were aligned using the Align X program and compared to the genomic sequence of the parental WNV isolate WN-NY99 (AF196835). Nucleotide sequences reported in this paper were deposited into the GenBank database and accession numbers are shown in Table 1 (KM012170—KM012188).

### 5. Phylogenetic analysis

For Maximum-likelihood phylogeny we used MEGA 6 [24]. The Maximum-likelihood method employing the General Time Reversible (GTR) + Γ + I model was used to produce phylogenetic trees. This model was determined using the selection tool available in MEGA 6. The parental strain WN-NY99 (AF196835) was used to root the trees. The 19 newly sequenced WNV strains from this study (Table 1) were aligned with 851 complete or near complete North American WNV sequences available in GenBank, as of September 2015, using MEGA 6. The dataset used in this study is composed of a total of 870 WNV ORF sequences from strains derived from the 1999–2012 epidemic seasons (1999, n = 13; 2000, n = 15; 2001, n = 84; 2002, n = 129; 2003, n = 176; 2004, n = 60; 2005, n = 60; 2006, n = 55; 2007, n = 49; 2008, n = 65; 2009, n = 36; 2010, n = 20; 2011, n = 31; 2012, n = 77), shown in S1 Table.

### 6. Selection pressure analysis

A selection analysis of ORFs of WNV strains isolated in 2012 (n = 77, S1 Table) was performed using the Datamonkey web-server (www.datamonkey.org). In addition to the Single-likelihood ancestor counting (SLAC), Internal Fixed effects likelihood (IFEL), Fixed effects likelihood (FEL), Random Effects likelihood (REL), Mixed Effects Model of Evolution (MEME), Fast, Unconstrained Bayesian Approximation for inferring selection (FUBAR) methods, and Evolutionary fingerprint, we also employed the Conant-Stadler Property Informed Models of Evolution (PRIME) method. We have used the PRIME method to study site-specific aa properties (e.g. chemical composition, charge, polarity) which are being conserved or altered by the evolutionary process. Because of Datamonkey server restrictions, the REL method was only used to evaluate 74 sequences, which was the largest dataset that could be successfully analyzed (KJ501432, KJ501434 and KJ501437 were excluded randomly).

### 7. Time-scale analysis

A Bayesian skyline plot (BSP) was used to estimate the viral effective population size through time. Evolutionary rates for the WNV ORF sequences (n = 870) were calculated using the Bayesian Markov-chain Monte Carlo (MCMC) approach employed by BEAST ver. 1.8.1 [25] and the BEAGLE library [26]. The dataset was analyzed using the TN93+Γ_4_ substitution model and the non-parametric Bayesian Skyline plot model, under relaxed uncorrelated lognormal (UCLN) molecular clocks as described elsewhere [21]. Four independent MCMC chains were run on a Tesla K20 computing processor until convergence to the stationary distribution was achieved (~500–600 million states with sampling frequency of 50,000). Posterior distributions were examined in Tracer v1.6 [27] to ensure adequate mixing and convergence. All chains were combined in LogCombiner with a burn-in value set to 30% of generations. The maximum clade credibility tree (MCC) and BSP (after resampling to 100,000) were generated. The MCC tree was visualized using FigTree v1.4.2 [27].

## Results

### 1. Nucleotide changes and amino acid substitutions

Complete genomic sequences from 19 studied isolates from the 2012 epidemic were compared to the prototype strain WN-NY99 (AF196835). Most mutations (~89%) were silent transitions (U↔C, A↔G). The total number of nt mutations ranged from 54 to 83. Shared nucleotide mutations identified in the studied WNV isolates are shown in Table 2. All 19 WNV 2012 isolates from this study shared 7 nt mutations (T_1442_C, C_2466_T, A_4146_G, C_4803_T, C_6426_T, C_6996_T and A_10851_G). Four mutations were shared by 18 of the 19 isolates: T_7938_C and T_8811_C (excepting BSL140); T_7015_C (excepting BSL178); and C_9352_T (excepting BSL85). In addition, seventeen isolates except BSL53 and BSL178 shared transition C_6138_T. Thirteen of the 19 completely sequenced isolates from 10 U.S. states (ND, SD, WY, TX, MS, GA, NM, OH, NE, IL) shared more than 50 nucleotide mutations when compared with prototype strain WN-NY99 (Table 2 and S2 Table).

**Table 2:** Common nucleotide mutations present in the 2012 human WNV isolates, compared to the prototype strain WN-NY99 (AF196835).

Gene	Capsid		prM	Env						NS1		NS2A						NS2B	NS3	
**Isolate/nt#**	**340**	**435**	**642**	**1442**	**1662**	**1750**	**2109**	**2304**	**2466**	**2475**	**3501**	**3528**	**3697**	**3912**	**3969**	**4088**	**4146**	**4530**	**4749**	**4803**
WN-NY99	**C**	**C**	**T**	**T**	**G**	**C**	**C**	**C**	**C**	**T**	**C**	**T**	**G**	**C**	**C**	**G**	**A**	**T**	**C**	**C**
BSL53	.	.	.	C	.	.	.	.	T	.	.	.	.	.	.	.	G	.	.	T
BSL178	.	.	.	C	.	.	.	.	T	.	.	.	.	.	.	.	G	.	.	T
BSL78	.	.	.	C	.	.	.	.	T	.	.	.	.	.	.	.	G	.	.	T
BSL85	.	.	C	C	.	.	.	.	T	.	.	.	.	.	.	.	G	.	.	T
BSL05	.	T	C	C	.	.	.	.	T	.	.	.	.	.	.	.	G	.	.	T
BSL80	.	T	C	C	.	.	.	.	T	.	.	.	.	.	.	.	G	.	.	T
BSL93	T	T	.	C	A	T	T	T	T	C	T	C	A	T	T	A	G	C	T	T
BSL101	T	T	.	C	A	T	T	T	T	C	T	C	A	T	T	A	G	C	T	T
BSL107	T	T	.	C	A	T	T	T	T	C	T	C	A	T	T	A	G	C	T	T
BSL116	T	T	.	C	A	T	T	T	T	.	T	C	.	T	T	A	G	C	T	T
BSL221	T	T	.	C	A	T	T	T	T	C	T	C	A	T	T	A	G	C	T	T
BSL08	T	T	.	C	A	T	T	T	T	.	T	C	.	T	T	A	G	C	T	T
BSL140	T	T	.	C	.	T	T	T	T	C	.	C	A	T	T	A	G	C	T	T
BSL195	T	T	.	C	.	T	.	T	T	.	.	C	.	.	T	A	G	C	.	T
ARC1	T	.	.	C	A	T	T	T	T	.	T	C	.	T	T	A	G	C	T	T
ARC3	T	T	.	C	A	T	.	T	T	.	.	C	.	.	T	A	G	C	.	T
ARC4	T	T	.	C	A	T	.	T	T	.	.	C	.	.	T	A	G	C	T	T
ARC6	T	T	.	C	A	T	T	T	T	C	T	C	A	T	T	A	G	C	T	T
ARC13	T	T	.	C	A	T	T	T	T	.	T	C	.	T	T	.	G	C	T	T
**Gene**	NS3									NS4A				NS4B						NS5
**Isolate/nt#**	**5400**	**5457**	**5889**	**6063**	**6138**	**6165**	**6312**	**6426**	**6540**	**6618**	**6639**	**6675**	**6996**	**7015**	**7395**	**7419**	**7527**	**7602**	**7635**	**7731**
WN-NY99	**T**	**C**	**C**	**G**	**C**	**T**	**T**	**C**	**T**	**T**	**T**	**C**	**C**	**T**	**A**	**C**	**C**	**C**	**A**	**C**
BSL53	.	.	.	.		.	.	T	.	.	.	.	T	C	.	.	.	.	.	.
BSL178	.	.	.	.		.	.	T	.	.	.	.	T	.	.	.	.	.	.	.
BSL78	.	.	.	.	T	.	.	T	.	.	.	.	T	C	.	.	.	.	.	.
BSL85	.	.	.	.	T	.	.	T	.	.	.	.	T	C	.	T	.	.	.	.
BSL05	.	.	.	.	T	.	.	T	.	.	.	.	T	C	.	.	.	.	G	.
BSL80	.	.	.	.	T	.	.	T	.	.	.	.	T	C	.	.	.	.	G	.
BSL93	C	T	T	T	T	C	C	T	C	C	C	T	T	C	C	T	T	T	G	T
BSL101	C	T	T	T	T	C	C	T	C	C	C	T	T	C	C	T	T	T	G	T
BSL107	C	T	T	T	T	C	C	T	C	C	C	T	T	C	C	T	T	T	G	T
BSL116	C	T	T	T	T	C	C	T	C	C	.	.	T	C	C	T	T	T	G	T
BSL221	C	T	T	T	T	C	C	T	C	C	C	T	T	C	C	T	T	T	G	T
BSL08	C	T	T	T	T	C	C	T	C	C	C	.	T	C	C	T	T	T	G	T
BSL140	C	T	.	.	T	C	C	T	C	C	C	T	T	C	C	T	T	T	G	.
BSL195	.	T	T	T	T	C	C	T	C	.	.	.	T	C	C	T	.	T	.	.
ARC1	C	T	T	T	T	C	C	T	C	C	.	.	T	C	C	T	T	T	G	T
ARC3	.	T	T	T	T	C	C	T	C	.	.	.	T	C	C	T	.	T	.	.
ARC4	.	.	T	T	T	C	C	T	C	C	.	.	T	C	C	T	T	T	G	T
ARC6	C	T	T	T	T	C	C	T	C	C	C	T	T	C	C	T	T	T	G	T
ARC13	.	.	T	T	T	C	C	T	C	C	.	.	T	C	.	.	T	T	G	T
**Gene**	NS5																		3'UTR	
**Isolate/nt#**	**7812**	**7893**	**7938**	**8361**	**8491**	**8565**	**8607**	**8622**	**8778**	**8811**	**8985**	**9136**	**9352**	**9381**	**9687**	**10248**	**10317**	**10393**	**10408**	**10851**
WN-NY99	**G**	**C**	**T**	**C**	**C**	**C**	**C**	**A**	**T**	**T**	**G**	**C**	**C**	**C**	**C**	**T**	**C**	**C**	**C**	**A**
BSL53	.	.	C	.	.	.	.	G	.	C	.	.	T	.	.	.	.	.	.	G
BSL178	.	.	C	.	.	.	.	.	.	C	.	.	T	.	.	.	.	.	.	G
BSL78	.	.	C	.	.	T	.	.	.	C	.	.	T	.	.	.	.	.	.	G
BSL85	.	.	C	.	.	.	.	.	.	C	.	T		.	.	.	.	T	T	G
BSL05	.	.	C	.	.	.	.	G	.	C	.	T	T	.	.	.	.	T	T	G
BSL80	.	.	C	.	.	.	.	G	.	C	.	T	T	.	.	.	.	T	T	G
BSL93	A	T	C	A	T	T	T	.	A	C	A	.	T	T	T	C	T	T	.	G
BSL101	A	T	C	A	T	T	T	.	A	C	A	.	T	T	T	.	T	.	.	G
BSL107	A	T	C	A	T	T	T	.	A	C	A	.	T	T	T	C	T	.	.	G
BSL116	A	T	C	A	T	T	T	.	A	C	A	.	T	T	T	C	T	.	.	G
BSL221	A	T	C	A	T	T	T	.	A	C	A	.	T	T	T	C	T	.	.	G
BSL08	A	T	C	A	T	T	T	.	A	C	A	.	T	T	T	C	.	T	.	G
BSL140	.	.	.	.	.	.	T	G	.	.	A	.	T	T	.	C	T	.	T	G
BSL195	.	.	C	.	T	.	T	.	A	C		.	T	.	.	C	.	.	.	G
ARC1	A	T	C	A	T	T	T	.	A	C	A	.	T	T	T	C	T	.	.	G
ARC3	.	.	C	.	T	.	T	.	A	C		.	T	.	.	C	.	.	.	G
ARC4	A	T	C	.	T	T	T	.	A	C	A	.	T	T	T	C	T	T	T	G
ARC6	A	T	C	A	T	T	T	.	A	C	A	.	T	T	T	C	T	.	T	G
ARC13	A	T	C	A	.	T	T	.	A	C	A	.	T	T	T	C	T	.	.	G

All nt mutations are shown in S2 Table. Comparison identified nt differences ranging from 54–83 (0.49%–0.75%) per 11,029-nt genome.

**Fig 1:**
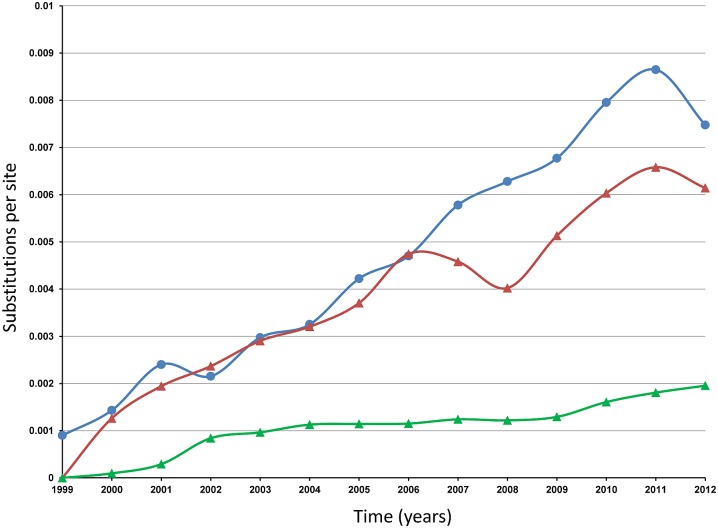
Increasing evolutionary divergence of North American WNV strains over the time of collection. Y-axis: Substitutions per site. X-axis: Years. Blue line: average divergence over sequence pairs within years; the numbers of base substitutions per site from averaging over all sequence pairs within each year are shown. Red line: divergence over sequence pairs between 1999 and other years; the numbers of base substitutions per site from averaging over all sequence pairs between 1999 and other years are shown. Green line: estimates of net evolutionary divergence between groups of sequences, 1999–2012: the numbers of base substitutions per site from estimation of net average between groups of sequences corresponding to each year are shown. Analyses were conducted in MEGA6 [24] using the Maximum Composite Likelihood model [46].

Among 2012 WNV isolates from this study, the number of deduced aa substitutions ranged from 4 to 13 when compared to WN-NY99, most of which are conservative changes. The transition T_1442_C is the non-silent mutation leading to the aa substitution E-V_449_A (V_159_A, in the Envelope protein numeration). This substitution is common for all WNV isolates collected in the U.S. since 2003, and therefore fixed in all strains of the WN02 and SW/WN03 genotypes. In addition to the aa substitution E-V_449_A, six WNV isolates shared NS2A-V_1201_I and 12 isolates shared the substitution NS2A-R_1331_K. Thirteen isolates reported here shared the substitution NS4B-I_2513_M (Table 3 and S3 Table). Analysis of the nt variation in the ORFs of the North American WNV dataset (n = 870, S1 Table) reveals increased evolutionary divergence from year to year (Fig 1). The estimated transition/transversion bias is 10.44 and the majority of the nt changes are transitions with relative rate 25.3 for U↔C and 7.2 for A↔G.

**Table 3:** Amino acid substitutions present in more than one of the 2012 human WNV isolates compared to the WN-NY99 (AF196835).

Protein	Capsid	E	NS1	NS2A		NS2B	NS4B		NS5		
isolate/aa#	104	449	1027	1201	1331	1494	2288	2513	2570	2577	2842
**WN-NY99**	**K**	**V**	**I**	**V**	**R**	**V**	**S**	**I**	**H**	**V**	**K**
BSL53	.	A	.	.	.	.	.	.	.	.	R
BSL178	.	A	.	.	.	.	N	.	.	.	.
BSL78	.	A	.	.	.	.	.	.	.	.	N
BSL85	.	A	.	.	.	.	R	.	.	.	.
BSL05	.	A	.	.	.	.	N	M	Y	.	R
BSL80	.	A	.	.	.	.	N	M	Y	.	R
BSL93	.	A	.	I	K	.	.	M	.	.	.
BSL101	.	A	.	I	K	.	.	M	.	.	.
BSL107	.	A	.	I	K	.	.	M	.	.	.
BSL116	.	A	.	.	K	.	.	M	.	.	.
BSL221	.	A	.	I	K	.	.	M	.	.	.
BSL08	.	A	.	.	K	.	.	M	.	.	.
BSL140	.	A	.	I	K	.	.	M	.	.	R
BSL195	R	A	V	.	K	I	.	.	.	I	.
ARC1	.	A	.	.	K	.	.	M	.	.	.
ARC3	R	A	V	.	K	I	.	.	.	I	.
ARC4	.	A	.	.	K	.	.	M	Y	.	.
ARC6	.	A	.	I	K	.	.	M	.	.	.
ARC13	.	A	.	.	.	.	.	M	.	.	.

Comparison of the 19 WNV isolates from the 2012 epidemic season with the WN-NY99 (AF196835) prototype strain identified aa substitutions ranging from 4–13 (0.11%–0.38%) per 3433-aa polyprotein. All aa substitutions are shown in S3 Table.

### 2. Phylogenetic analysis

**Fig 2:**
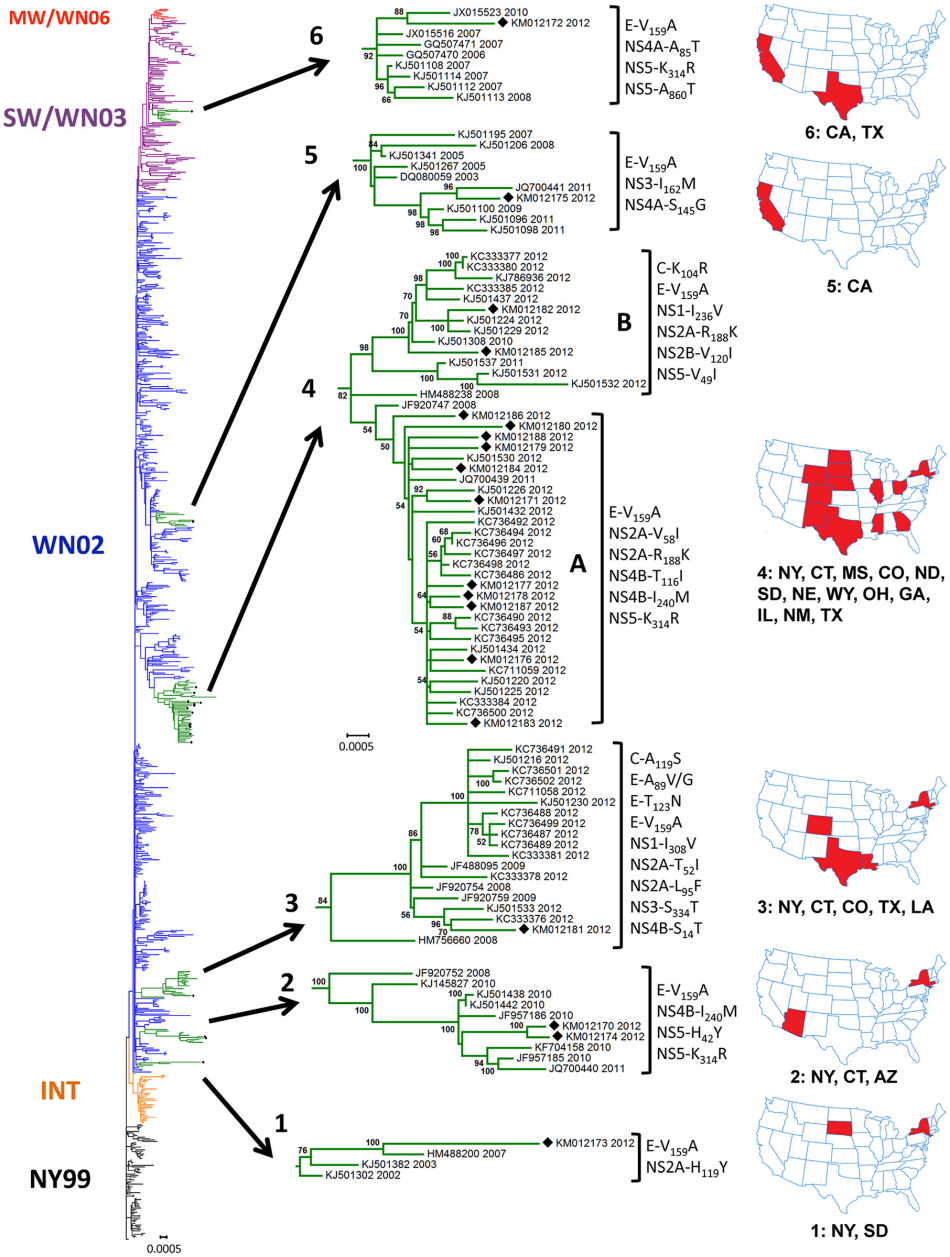
Consensus maximum-likelihood tree of North American WNV ORFs, 1999–2012 (n = 870). WNV genotypes are color-coded as NY99 (black), INTermediate (orange), WN02 (blue), SW/WN03 (purple) and cluster MW/WN06 (red). All WNV sequences derived from this study are labeled by black diamonds, and Nodes 1 to 6 containing these sequences are highlighted in green and shown in detail. Taxon names correspond to GenBank accession numbers and years of collection. Node-specific amino acid substitutions are shown for each node (see also S4 Table). For each node, states shown in red in the adjacent U.S. map are those from which strains have been isolated.

Phylogenetic analysis was performed using the Maximum-likelihood method. In addition to the 19 WNV ORFs sequenced in this study, the North American WNV ORF sequences available from the GenBank database, as of September 2015, were included in the dataset (n = 870, S1 Table). We have analyzed the phylogeny of these sequences and identified, as expected, the presence of the common clades representing the North American WNV genotypes NY99, WN02 and SW/WN03, previously described in the course of study of WNV evolution in North America [11, 16–23, 28–39] (Fig 2 and S1 Fig). The 2012 WNV human isolates from this study are located within six nodes termed here “Node 1” to “Node 6”. Node-specific aa substitutions and geographical origin of isolates are shown in Fig 2 and S4 Table. We have observed that all WNV isolates reported here except BSL53 (KM012172), which is clustered in Node 6 within the SW/WN03 genotype, belong to the WN02 genotype. All studied isolates carried the common North American WNV aa substitution E-V_159_A which is fixed in the WN02 and SW/WN03 genotypes and present in all WNV strains collected in the U.S. since 2003.

Analysis of the entire ORF of WNV isolates circulating in the U.S. has shown that two isolates from AZ, BSL05 (KM012170) and BSL80 (KM012174), clustered together with previously published isolates from AZ in Node 2, and an isolate from CA, BSL85 (KM012175), clustered with other isolates from CA in Node 5. We found that the WNV isolate BSL178 from LA (KM012181) was associated with Node 3, which mainly consisted of previously published WNV strains collected from TX in 2012 [22, 23] and two 2012 isolates from CO. WNV strains presented in Node 3 shared up to nine aa substitutions. Surprisingly, many of the published 2012 isolates (n = 27) were clustered within Node 4 together with 13 genetically related WNV isolates from this study collected from 10 states: ND, SD, WY, TX, MS, GA, NM, OH, NE and IL. All WNV strains from Node 4, except KM012188 and KJ501532, shared the NS2A-R_188_K aa substitution in addition to the common E-V_159_A. Other node-specific aa substitutions are shown in S4 Table.

### 3. Selection pressure analysis

**Fig 3:**
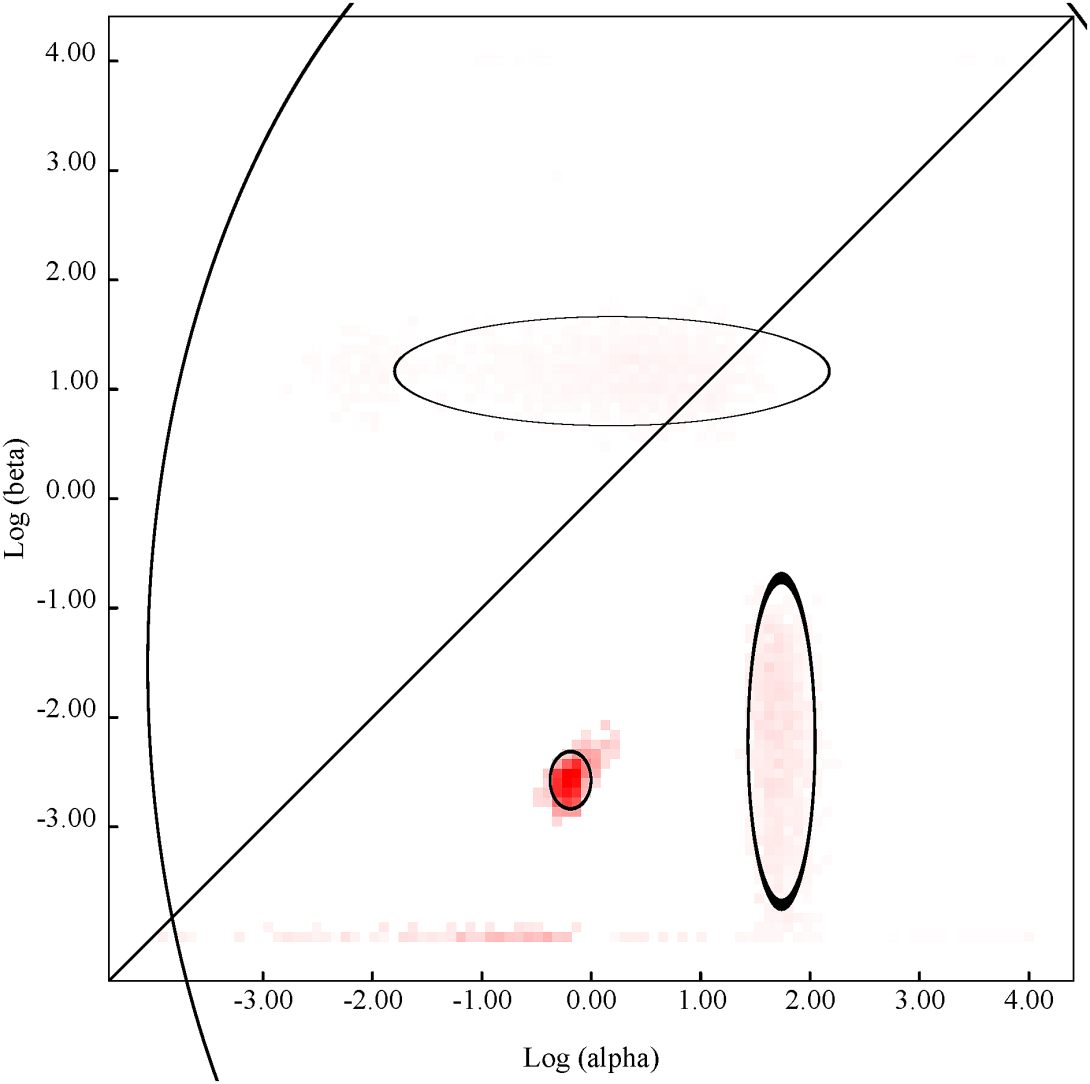
Evolutionary fingerprint based on 1000 distribution samples (Datamonkey server www.datamonkey.org). The plot depicts the estimate of the distribution of synonymous and non-synonymous rates inferred from alignment of WNV sequences (n = 77) from strains collected in the US in 2012. The ellipses reflect a Gaussian-approximated variance in each individual rate estimate, and colored pixels show the density of the posterior sample of the distribution for a given rate. The diagonal line represents the idealized neutral evolution scenario (ω = 1), points above the line correspond to positive selection (ω>1), and points below the line to negative selection (ω<1).

Using different codon-based and branch-site approaches, we detected a number of codons subjected to positive pressure in WNV strains collected in 2012 (n = 74 for REL, n = 77 for all other methods). Analysis was done using the DataMonkey web-server (www.datamonkey.org). We found that eight codons: 379; 1083; 1195; 1238; 1494; 2288; 2389; and 2842; were detected as positively selected by at least two methods. Site 2842, corresponding to the NS5-K_314_R aa substitution, was the only site identified as positively selected by all methods (Table 4). Eleven node-specific aa substitutions identified in the phylogenetic analysis and detected as positively selected by at least one method are shown in Table 5. We performed evolutionary fingerprint analysis, which models site to site variation in selection pressure across the ORF, for WNV isolates from 2012 (n = 77) (Fig 3). The colored pixels on this plot show the density of the posterior sample of the distribution for a given rate and ellipses reflect a Gaussian-approximated variance in each individual rate estimate. Points above the diagonal line corresponded to positive selection (ω>1), and points below the diagonal line corresponded to negative selection (ω<1). Most of the points are concentrated below the diagonal line which represents the idealized neutral evolution scenario (ω = 1). The results suggest that WNV strains collected in 2012 were subjected to strong negative purifying selection. In addition, we conducted a supplementary selection pressure analysis using PRIME to detect whether selection for amino acids with differing chemical properties is occurring within the 2012 dataset (n = 77). Conant-Stadler PRIME analysis allows the non-synonymous substitution rate β to depend not only on the site in question (like FEL and MEME), but also on which residues are being exchanged. Substitution rate analysis identified a single rate class, which suggests that across the 2012 WNV isolates, the rate of substitution between each residue was similar and no particular substitution was favored. PRIME analysis detected an overall substitution rate of 0.05 substitutions/codon site. One site, codon 2842, was negatively selected for volume and positively selected for changes in chemical composition. This codon, corresponding to the NS5-K_314_R aa substitution, was identified as positively selected by all methods used for study of selection pressure.

**Table 4:** Selection pressure analysis of WNV strains collected in the U.S. in 2012.

	Method:	SLAC		IFEL		FEL		MEME		FUBAR		REL*	
Codon	Protein aa #	dN/dS	p-value	dN/dS	p-value	dN/dS	p-value	ω^+^	p-value	dN/dS	Post. Pr.	dN/dS	Bayes Factor
379	E_89_							>100	0.025			0.244	85.5
1083	NS1_292_							>100	0.000			0.430	87.1
1195	NS2A_52_			69.981	0.021	35.309	0.038			2.466	0.934	2.228	220.7
1238	NS2A_95_			64.003	0.037							0.469	90.2
1494	NS2B_120_			41.204	0.087							0.305	88.5
2288	NS4B_15_			65.971	0.057	52.724	0.023			3.494	0.926	0.700	118.3
2389	NS4B_116_							>100	0.082			0.220	85.8
2842	NS5_314_	30.211	0.060	150.347	0.067	158.746	0.029	>100	0.035	16.399	0.995	5.079	10433.7
	nss #	94		52		285		na		981		58	

SLAC = Single-Likelihood Ancestor Counting; IFEL = Internal Fixed effects likelihood; FEL = Fixed Effects Likelihood; REL* = Random Effects Likelihood (done on reduced dataset n = 74); MEME = Mixed Effects Model of Evolution; FUBAR = Fast, Unconstrained Bayesian AppRoximation for inferring selection. All codons present in the table are recognized by at least two methods with p threshold < 0.1; FUBAR Posterior Probability > 0.9; and/or REL Bayes Factor > 50; nss# is a number of negatively selected sites; na = not available.

**Table 5:** Positively selected node-specific aa substitutions.

Protein	E	NS2A	NS2A	NS2A	NS2A	NS2B
Codon/aa prot #	379/89	1195/52	1238/95	1262/119	1331/188	1494/120
Node	3	3	3	1	4	4
Method/p-value or Bayes Factor	REL/85.46	REL/220.73	REL/90.23	REL/87.14	REL/118.29	REL/88.51
	MEME/0.025	FEL/0.038	IFEL/0.037			IFEL/0.087
		FUBAR/109.24				
		IFEL/0.021				
Protein	NS3	NS4A	NS4B	NS4B	NS5	
Codon/aa prot #	1667/162	2269/145	2389/116	2513/240	2842/314	
Node	5	5	4	2, 4	2, 4, 6	
Method/p-value or Bayes Factor	REL/53.64	FEL/0.091	REL/85.77	REL/1456.09	REL/10433.7	
			MEME/0.081		SLAC/0.059	
					FEL/0.029	
					MEME/0.035	
					FUBAR/1459.18	
					IFEL/0.067	

SLAC = Single-Likelihood Ancestor Counting; IFEL = Internal Fixed effects likelihood; FEL = Fixed Effects Likelihood; REL* = Random Effects Likelihood (done on reduced dataset n = 74); MEME = Mixed Effects Model of Evolution; FUBAR = Fast, Unconstrained Bayesian AppRoximation for inferring selection. p threshold < 0.1; FUBAR Posterior Probability > 0.9; and/or REL Bayes Factor > 50.

### 4. Time-scale analysis

**Fig 4:**
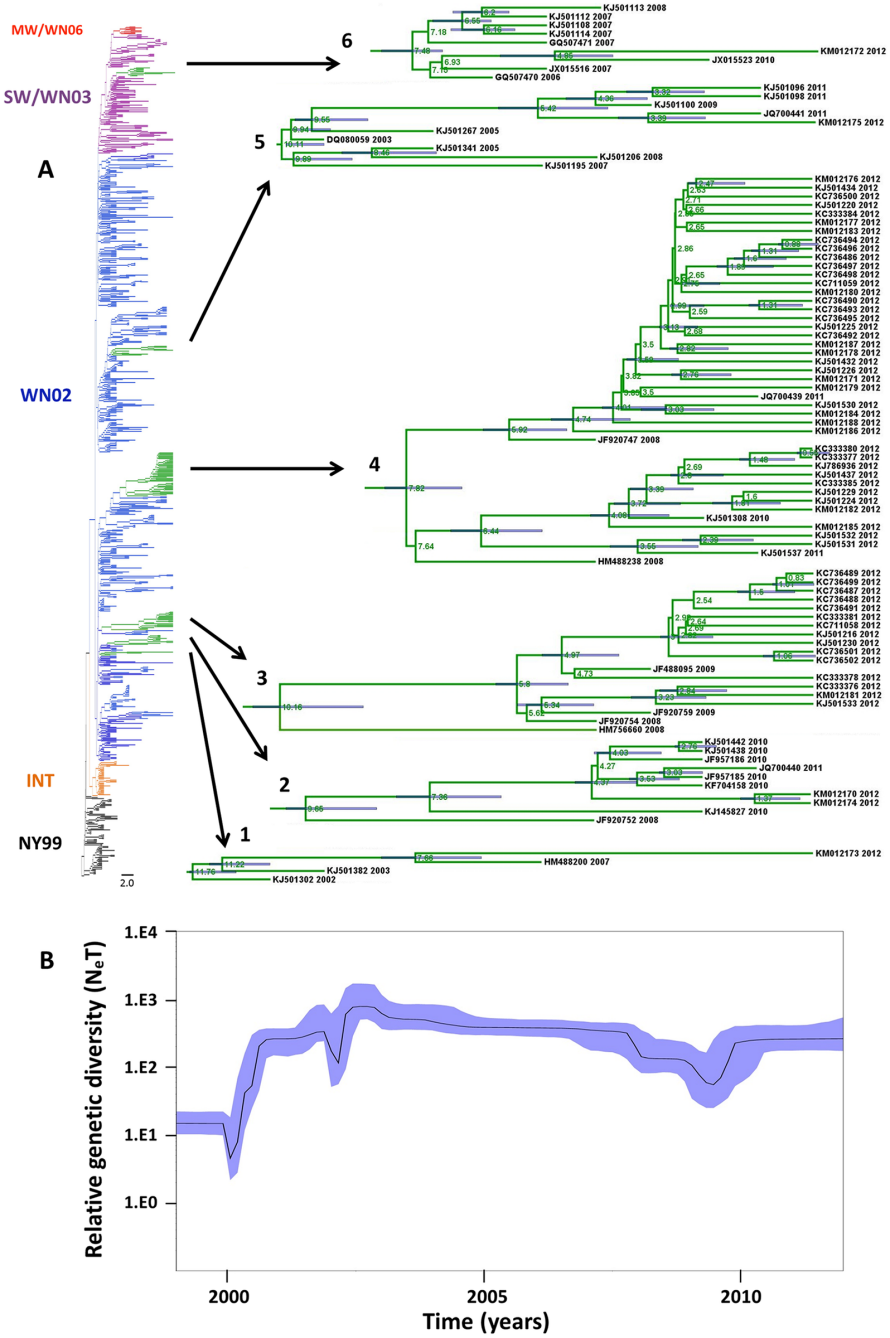
Maximum clade credibility tree from Bayesian analysis of WNV strains from North America, 1999–2012 (n = 870). A) WNV genotypes are color-coded in the branches of the tree as NY99 (black), WN02 (blue), SW/WN03 (purple) and cluster MW/WN06 (red). Nodes 1 to 6 containing WNV isolates from this study are highlighted in green and shown in detail. The mean time to the most recent common ancestor (tMRCA) is shown in each principal node. The 95% highest probability densities (95% HPD) for each node age are shown as blue bars. B) Bayesian coalescent inference of genetic diversity and population dynamics using the Bayesian Skyline plot. The X axis represents years of study and the Y axis, the relative genetic diversity product of the effective population size.

The time-scale analysis was performed using the North American WNV dataset (n = 870, S1 Table) and the non-parametric Bayesian Skyline plot (BSP) model available in BEAST 1.8.1. Previously we found that the BSP with the relaxed molecular clock (UCLN) was the best-fitted model [21]. The maximum clade credibility tree (MCC) was selected and the age for each node containing studied WNV isolates is shown on Fig 4A and S2 Fig. The time to most recent common ancestor (tMRCA) for the entire dataset was 14.78 years ago. Compared to the maximum-likelihood and Bayesian consensus phylogenetic trees, the MCC tree demonstrated a similar topology.

Bayesian coalescent inference of genetic diversity and population dynamics was visualized using the Bayesian Skyline plot available in BEAST (Fig 4B). The plot shows that a period of high genetic variability was observed until 2003 corresponding with the appearance of the new North American genotypes. From 2003–2009, genetic diversity of the U.S. WNV population decreased slightly, with a maximum decrease occurring around 2008–2009. A small increase in diversity occurred after 2009, and the overall diversity of the WNV population then continued to increase through 2012.

## Discussion

WNV now is the most widespread and common cause of viral encephalitis in the U.S. and worldwide [11, 12]. After six years of relatively low WNV incidence in the U.S., a large outbreak was observed in 2012 causing 5,674 total disease cases and 286 deaths, the largest number of deaths ever reported [9]. In this study we investigated the genetic variability of 19 WNV strains isolated from human samples collected in 2012 from 13 states of the U.S. (Table 1). Although humans are considered dead-end hosts for WNV, and therefore, not important for the WNV lifecycle, human isolates represent circulating viruses. Studying human WNV isolates is also important for public health and for the safety of the blood supply.

Multiple factors were potentially involved in the magnitude of the 2012 outbreak. In addition to ecological and environmental factors that have been shown to increase viral transmission [40, 41], viral genetics and selection of new best-fitted variants may play a significant role in WNV outbreaks. Viral adaptation to domestic mosquitoes and birds has played a major role in the spread of WNV in the U.S. since its introduction in 1999. WNV has continued to evolve, as illustrated through the displacement of the ancestor genotype WN99 by the new genotype WN02 in 2002, followed by the appearance and co-circulation of genotype SW/WN03 in 2003 and subtype MW/WN06 in 2006 [11, 16–23, 28–39]. Analysis of nucleotide divergence of newly sequenced isolates from this study together with published North American WNV strains (n = 870) demonstrates increasing evolutionary divergence from year to year (Fig 1).

Previous phylogenetic analysis of WNV isolates shows that with limited exceptions, WNV isolates from circulating genotypes in the U.S. were poorly differentiated spatially and temporally [21]. It has been postulated that WNV genetic variations in the U.S. have occurred in some geographic areas which function as distinct niches of evolution. In these areas, the genetic variant accumulates genetic changes while adapting to the local ecological conditions, hosts and vectors, and may either stay in that area or be disseminated to other regions by migrating birds [42]. We observed that isolate BSL178 from LA was grouped in Node 3 together with WNV strains collected from TX in 2012 [22, 23] and two 2012 isolates from CO. Thirteen other genetically similar human isolates from samples collected in 10 U.S. states for this study clustered with 2012 mosquito and bird isolates from TX [22, 23] and CO in Node 4 (Fig 2). Nodes 3 and 4 are good examples of strong temporal phylogenetic structures constituted by well temporally differentiated isolates, and they were composed predominantly of isolates collected in the 2012 epidemic season. In contrast, isolates from AZ and CA clustered within local Nodes 2 and 5. These nodes are good examples of strong spatial phylogenetic structures, which are supported by high bootstrapping values. The finding of similar isolates across a broad geographic area in 2012 suggests that closely related genetic variants of WNV represented in Node 4 spread over the Atlantic, Mississippi and Central bird flyways, but not the Pacific, and were identified coincident with the largest U.S. WNV outbreak since 2003. In CA and AZ, both of which are located on the Pacific bird flyway, specimens clustered with local circulating clades suggesting predominantly local scale evolution in this area [21, 39, 43].

Previous studies of 2012 U.S. isolates have suggested that viral genetic composition was not a determinant of outbreak intensity at the local level. Duggal et al. noted that the genetic composition of viruses circulating in Texas in 2012 was similar between isolates from a county that experienced a large outbreak (Dallas County) and a county that didn’t (Montgomery County) [22]. Our data supports this conclusion on a broader geographic basis, because WNV isolates from the Nodes 3 and 4 circulated alongside isolates that were similar to those that circulated in 2008–2011, and high numbers of disease cases occurred in areas where isolates from these Nodes were not detected at all, such as CA. Rather, increased replication in a favorable environment may have provided opportunity for genetically related co-existing strains to circulate and spread over migratory bird flyways, as has been reported on a local scale in TX and AZ [22, 23, 39].

The degree of genetic diversity and fitness of viral population is a balance between positive or negative selection and genetic drift as accumulation of random neutral mutations [44]. Previous studies have shown a low level of positive selection in WNV isolates from the U.S. [21, 34, 36] suggesting that most aa changes were the result of genetic drift. In our study of WNV isolates from 2012, selection pressure analysis revealed only one site that was positively selected by all employed methods, codon 2842 (NS5_314_). This site has been previously identified as subject to positive selection in other studies of North American WNV sequences [21, 22, 37]. We found that this site is associated with nodes 2, 4 and 6 (Tables 4, 5 and S4) and aa substitution NS5-K_314_R is involved in the emergence of the SW/WN03 genotype [20, 21]. Site 1195 in NS2A was detected as positively selected by four methods. This site is associated with Node 3 aa substitution NS2A-T_52_I. Overall for the 2012 isolates, three aa substitutions in Node 3 and five in Node 4 were identified as positively selected by at least one method. Potentially aa substitutions could impact viral fitness and virulence, and the biological significance of those changes in viral proteins warrants further investigation. In general, our results are consistent with previous studies which have demonstrated that only limited positive selection is acting on the population of WNV circulating in the U.S., and purifying selection is predominant [21, 22].

In previous studies, results of time-scale analysis were only reported for select genes of WNV or reduced datasets [17, 21, 34, 35, 45]. In this study we performed comprehensive time-scale analysis using 870 full-length ORFs of WNV strains isolated in the U.S. in 1999–2012 (Fig 4A). We found that the time to most recent common ancestor (tMRCA) for the whole dataset (n = 870) was 14.78 years (95% HPD = 13.87–15.49 years), which is consistent with the value of 15.57 years (95% HPD = 14.23–16.98 years) previously reported in the study of human isolates (n = 62) when strain IS-98 (AF481864) from 1998 was used to root the tree [21]. We calculated the mean nucleotide substitution rate (MNSR), using the BSP model with the relaxed molecular clock, to be 6.81 x 10^-4^ substitutions/site/year (s/s/y), which also correlates with published data [21, 36]. Analysis of the BSP (Fig 4B) shows that genetic divergence had continued to slowly increase through 2012 following a brief period of contraction in 2008–2009, which also agrees with data published by us and others [21, 36, 45].

Overall, our findings in this study suggest that the patterns of WNV genetic evolution in the U.S. following the 2012 outbreak remained consistent with previous trends. Additionally, our observation of the broad geographic distribution of genetically similar isolates suggests that these WNV variants may have spread via migratory birds, and were detected coincident with the largest WNV outbreak since 2003. The emergence of this genetic variant may potentially mark the beginning of a new genetic shift and spread of a new WNV genotype after 10 years of steady drift.

## Supporting Information

S1 Table List of North American WNV strains used in this study. (DOCX)

S2 Table Nucleotide mutations present in 2012 human WNV isolates, compared to the prototype strain WN-NY99 (AF196835). (DOCX)

S3 Table Amino acid substitutions present in 2012 human WNV isolates, compared to the prototype strain WN-NY99 (AF196835). (DOCX)

S4 Table Node-specific amino acid substitutions. (DOCX)

S1 Fig Consensus maximum-likelihood tree of North American WNV ORFs, 1999–2012 (n = 870). WNV genotypes are color-coded as NY99 (black), INTermediate (orange), WN02 (blue), SW/WN03 (purple) and cluster MW/WN06 (red). All WNV sequences derived from this study are labeled by black diamonds, and Nodes 1 to 6 containing these sequences are highlighted in green.
(TIFF)

S2 Fig Maximum clade credibility tree from Bayesian analysis of WNV strains from North America, 1999–2012 (n = 870). WNV genotypes are color-coded in the branches of the tree as NY99 (black), WN02 (blue), SW/WN03 (purple) and cluster MW/WN06 (red). Nodes 1 to 6 containing WNV isolates from this study are highlighted in green. The mean time to the most recent common ancestor (tMRCA) is shown in each principal node. The 95% highest probability densities (95% HPD) for each node age are shown as blue bars.
(TIFF)
