# Supplementary material for: Genetic Variability of West Nile Virus in U.S. Blood Donors from the 2012 Epidemic Season
Source: PLoS Negl Trop Dis. 2016 May 16;10(5):e0004717. doi: 10.1371/journal.pntd.0004717 (PMC4868353; doi:10.1371/journal.pntd.0004717)
Supplement: S1 Table — (DOCX) [file pntd.0004717.s001.docx]

**Supplemental Table 1.** List of North American WNV strains used in this study.

| # | GenBank accession # | Strain name | Year of | State/ |
| --- | --- | --- | --- | --- |
|  |  |  | Isolation | Country |
| 1 | AF196835 | NY99-flamingo382-99 | 1999 | NY |
| 2 | AF260967 | NY99-eqhs | 1999 | NY |
| 3 | AF202541 | HNY1999 | 1999 | NY |
| 4 | AF206518 | 2741 | 1999 | CT |
| 5 | HM488127 | BID-V4188 | 1999 | CT |
| 6 | HM488128 | BID-V4189 | 1999 | CT |
| 7 | HM488125 | BID-V4186 | 1999 | CT |
| 8 | HM488126 | BID-V4187 | 1999 | CT |
| 9 | FJ411043 | NY99iso-1 | 1999 | NY |
| 10 | HQ671706 | BID-V4898 | 1999 | CT |
| 11 | HQ671707 | BID-V4899 | 1999 | CT |
| 12 | FJ151394 | NY99-crow-V76/1 | 1999 | NY |
| 13 | HQ596519 | 4132 | 1999 | NY |
| 14 | HQ671710 | BID-V4902 | 2000 | CT |
| 15 | HQ671709 | BID-V4901 | 2000 | CT |
| 16 | HQ671711 | BID-V4903 | 2000 | CT |
| 17 | HQ671712 | BID-V4904 | 2000 | CT |
| 18 | EF657887 | 3356K VP2 | 2000 | NY |
| 19 | EF530047 | 3356.2.1.1 | 2000 | NY |
| 20 | HQ671708 | BID-V4900 | 2000 | CT |
| 21 | AF404753 | MD 2000-crow265 | 2000 | MD |
| 22 | HM488129 | BID-V4191 | 2000 | CT |
| 23 | HM488130 | BID-V4192 | 2000 | CT |
| 24 | HM488131 | BID-V4193 | 2000 | CT |
| 25 | AF404756 | NY 2000-crow3356 | 2000 | NY |
| 26 | AF404755 | NY 2000-grouse3282 | 2000 | NY |
| 27 | AF404754 | NJ 2000 MQ5488 | 2000 | NJ |
| 28 | HM488132 | BID-V4194 | 2000 | CT |
| 29 | HQ671719 | BID-V4912 | 2001 | CT |
| 30 | HQ671697 | BID-V4197 | 2001 | CT |
| 31 | HM756663 | BID-V4697 | 2001 | NY |
| 32 | HM756661 | BID-V4692 | 2001 | NY |
| 33 | HM756662 | BID-V4693 | 2001 | NY |
| 34 | HQ671717 | BID-V4910 | 2001 | CT |
| 35 | HQ671718 | BID-V4911 | 2001 | CT |
| 36 | HQ671715 | BID-V4908 | 2001 | CT |
| 37 | HQ671713 | BID-V4905 | 2001 | CT |
| 38 | HQ671714 | BID-V4906 | 2001 | CT |
| 39 | KJ501452 | BID-V6405 | 2001 | USA |
| 40 | KJ501453 | BID-V6407 | 2001 | USA |
| 41 | KJ501445 | BID-V6381 | 2001 | USA |
| 42 | KJ501448 | BID-V6393 | 2001 | USA |
| 43 | HM488136 | BID-V4200 | 2001 | CT |
| 44 | HM488133 | BID-V4195 | 2001 | CT |
| 45 | KJ501317 | BID-V6479 | 2001 | USA |
| 46 | HM488135 | BID-V4199 | 2001 | CT |
| 47 | HM488134 | BID-V4198 | 2001 | CT |
| 48 | HM488249 | BID-V4696 | 2001 | NY |
| 49 | HM488248 | BID-V4694 | 2001 | NY |
| 50 | KJ501288 | BID-V6420 | 2001 | USA |
| 51 | KJ501289 | BID-V6422 | 2001 | USA |
| 52 | HM488247 | BID-V4691 | 2001 | NY |
| 53 | KJ501439 | BID-V6194 | 2001 | USA |
| 54 | KJ501443 | BID-V6373 | 2001 | USA |
| 55 | HM488246 | BID-V4689 | 2001 | NY |
| 56 | KJ501292 | BID-V6425 | 2001 | USA |
| 57 | KJ501283 | BID-V6409 | 2001 | USA |
| 58 | KJ501516 | BID-V6625 | 2001 | USA |
| 59 | KJ501468 | BID-V6458 | 2001 | USA |
| 60 | KJ501374 | BID-V6622 | 2001 | USA |
| 61 | KJ501509 | BID-V6575 | 2001 | USA |
| 62 | KJ501512 | BID-V6616 | 2001 | USA |
| 63 | KJ501515 | BID-V6620 | 2001 | USA |
| 64 | KJ786934 | NY2001-6263 | 2001 | NY |
| 65 | KJ501465 | BID-V6450 | 2001 | USA |
| 66 | KJ501393 | BID-V6649 | 2001 | USA |
| 67 | KJ501377 | BID-V6628 | 2001 | USA |
| 68 | KJ501378 | BID-V6629 | 2001 | USA |
| 69 | KJ501379 | BID-V6630 | 2001 | USA |
| 70 | KJ501343 | BID-V6527 | 2001 | USA |
| 71 | KJ501361 | BID-V6566 | 2001 | USA |
| 72 | KJ501362 | BID-V6567 | 2001 | USA |
| 73 | KJ501480 | BID-V6478 | 2001 | USA |
| 74 | KJ501486 | BID-V6496 | 2001 | USA |
| 75 | KJ501493 | BID-V6526 | 2001 | USA |
| 76 | KJ501496 | BID-V6531 | 2001 | USA |
| 77 | KJ501497 | BID-V6532 | 2001 | USA |
| 78 | KJ501507 | BID-V6556 | 2001 | USA |
| 79 | KJ501363 | BID-V6568 | 2001 | USA |
| 80 | KJ501494 | BID-V6528 | 2001 | USA |
| 81 | KJ501473 | BID-V6469 | 2001 | USA |
| 82 | KJ501264 | BID-V6374 | 2001 | USA |
| 83 | KJ501268 | BID-V6379 | 2001 | USA |
| 84 | KJ501269 | BID-V6380 | 2001 | USA |
| 85 | KJ501248 | BID-V6192 | 2001 | USA |
| 86 | KJ501249 | BID-V6193 | 2001 | USA |
| 87 | KJ501332 | BID-V6505 | 2001 | USA |
| 88 | KJ501277 | BID-V6394 | 2001 | USA |
| 89 | KJ501278 | BID-V6396 | 2001 | USA |
| 90 | KJ501282 | BID-V6406 | 2001 | USA |
| 91 | JF920307 | BID-V4907 | 2001 | CT |
| 92 | KJ501274 | BID-V6390 | 2001 | USA |
| 93 | KJ501275 | BID-V6391 | 2001 | USA |
| 94 | KJ501234 | BID-V6175 | 2001 | USA |
| 95 | KJ501235 | BID-V6176 | 2001 | USA |
| 96 | KJ501401 | BID-V6659 | 2001 | USA |
| 97 | KJ501394 | BID-V6650 | 2001 | USA |
| 98 | KJ501395 | BID-V6651 | 2001 | USA |
| 99 | KJ501233 | BID-V6173 | 2001 | USA |
| 100 | KJ501245 | BID-V6189 | 2001 | USA |
| 101 | KJ501246 | BID-V6190 | 2001 | USA |
| 102 | KJ501247 | BID-V6191 | 2001 | USA |
| 103 | KJ501336 | BID-V6517 | 2001 | USA |
| 104 | KJ501243 | BID-V6187 | 2001 | USA |
| 105 | KJ501244 | BID-V6188 | 2001 | USA |
| 106 | KJ501455 | BID-V6418 | 2001 | USA |
| 107 | KJ501454 | BID-V6408 | 2001 | USA |
| 108 | AF533540 | NY_2001 | 2001 | NY |
| 109 | FJ527738 | LSU-AR01 | 2001 | LA |
| 110 | DQ080072 | FL232 | 2001 | FL |
| 111 | GQ379156 | FL2001_67030 | 2001 | FL |
| 112 | DQ164194 | NY 2001 Suffolk | 2001 | NY |
| 113 | DQ164200 | IN 2002 | 2002 | IN |
| 114 | KJ501270 | BID-V6382 | 2002 | USA |
| 115 | DQ164202 | OH 2002 | 2002 | OH |
| 116 | KJ501266 | BID-V6376 | 2002 | USA |
| 117 | KJ501263 | BID-V6372 | 2002 | USA |
| 118 | KJ501265 | BID-V6375 | 2002 | USA |
| 119 | HM488184 | BID-V4346 | 2002 | IL |
| 120 | JN183891 | BID-V4344 | 2002 | IL |
| 121 | HM488183 | BID-V4345 | 2002 | IL |
| 122 | KJ501295 | BID-V6431 | 2002 | USA |
| 123 | KJ501458 | BID-V6432 | 2002 | USA |
| 124 | DQ080062 | TWN165 | 2002 | LA |
| 125 | KJ501474 | BID-V6470 | 2002 | USA |
| 126 | JF730043 | BID-V5170 | 2002 | CT |
| 127 | KJ501296 | BID-V6433 | 2002 | USA |
| 128 | KJ501495 | BID-V6530 | 2002 | USA |
| 129 | KJ501328 | BID-V6498 | 2002 | USA |
| 130 | KJ501511 | BID-V6613 | 2002 | USA |
| 131 | KJ501333 | BID-V6510 | 2002 | USA |
| 132 | KJ501300 | BID-V6443 | 2002 | USA |
| 133 | DQ164195 | NY_2002_Nassau | 2002 | NY |
| 134 | DQ164197 | GA_2002_2 | 2002 | GA |
| 135 | DQ164196 | GA_2002_1 | 2002 | GA |
| 136 | KJ501510 | BID-V6612 | 2002 | USA |
| 137 | KJ501476 | BID-V6473 | 2002 | USA |
| 138 | DQ164193 | NY_2002_Clinton | 2002 | NY |
| 139 | KJ501331 | BID-V6502 | 2002 | USA |
| 140 | KJ501257 | BID-V6208 | 2002 | USA |
| 141 | HM488208 | BID-V4204 | 2002 | CT |
| 142 | KJ501298 | BID-V6436 | 2002 | USA |
| 143 | KJ501299 | BID-V6438 | 2002 | USA |
| 144 | DQ164198 | TX_2002_1 | 2002 | TX |
| 145 | KJ501505 | BID-V6550 | 2002 | USA |
| 146 | KJ501383 | BID-V6634 | 2002 | USA |
| 147 | KJ501284 | BID-V6410 | 2002 | USA |
| 148 | KJ501364 | BID-V6569 | 2002 | USA |
| 149 | KJ501286 | BID-V6413 | 2002 | USA |
| 150 | KJ501285 | BID-V6412 | 2002 | USA |
| 151 | HQ671742 | BID-V4343 | 2002 | IL |
| 152 | HM756665 | BID-V4709 | 2002 | NY |
| 153 | HM756664 | BID-V4701 | 2002 | NY |
| 154 | HQ705669 | BID-V4342 | 2002 | IL |
| 155 | KJ501475 | BID-V6471 | 2002 | USA |
| 156 | KJ501355 | BID-V6558 | 2002 | USA |
| 157 | KJ501467 | BID-V6453 | 2002 | USA |
| 158 | HQ671720 | BID-V4913 | 2002 | CT |
| 159 | KJ501373 | BID-V6617 | 2002 | USA |
| 160 | KJ501371 | BID-V6611 | 2002 | USA |
| 161 | KJ501427 | BID-V6699 | 2002 | USA |
| 162 | HQ671698 | BID-V4203 | 2002 | CT |
| 163 | KJ501287 | BID-V6419 | 2002 | USA |
| 164 | HQ671722 | BID-V4704 | 2002 | NY |
| 165 | HQ671699 | BID-V4206 | 2002 | CT |
| 166 | KJ501290 | BID-V6423 | 2002 | USA |
| 167 | KJ501291 | BID-V6424 | 2002 | USA |
| 168 | KJ501466 | BID-V6451 | 2002 | USA |
| 169 | HM756648 | BID-V4205 | 2002 | CT |
| 170 | AY646354 | NY 2002 | 2002 | NY |
| 171 | KJ501380 | BID-V6631 | 2002 | USA |
| 172 | KJ501491 | BID-V6508 | 2002 | USA |
| 173 | KJ501514 | BID-V6619 | 2002 | USA |
| 174 | AY795965 | ARC10 | 2002 | MI |
| 175 | KJ501376 | BID-V6627 | 2002 | USA |
| 176 | KJ501517 | BID-V6626 | 2002 | USA |
| 177 | KJ501281 | BID-V6404 | 2002 | USA |
| 178 | KJ501519 | BID-V6652 | 2002 | USA |
| 179 | DQ176637 | TX_2002-HC | 2002 | TX |
| 180 | KJ501279 | BID-V6401 | 2002 | USA |
| 181 | DQ164205 | TX 2002_2 | 2002 | TX |
| 182 | KJ501276 | BID-V6392 | 2002 | USA |
| 183 | KJ501375 | BID-V6623 | 2002 | USA |
| 184 | KJ501426 | BID-V6698 | 2002 | USA |
| 185 | KJ501400 | BID-V6658 | 2002 | USA |
| 186 | HM488114 | BID-V4102 | 2002 | CT |
| 187 | KJ501388 | BID-V6643 | 2002 | USA |
| 188 | KJ501503 | BID-V6545 | 2002 | USA |
| 189 | KJ501344 | BID-V6529 | 2002 | USA |
| 190 | KJ501481 | BID-V6483 | 2002 | USA |
| 191 | KJ501502 | BID-V6542 | 2002 | USA |
| 192 | KJ501309 | BID-V6459 | 2002 | USA |
| 193 | KJ501464 | BID-V6448 | 2002 | USA |
| 194 | JN183887 | BID-V4706 | 2002 | NY |
| 195 | KJ501310 | BID-V6460 | 2002 | USA |
| 196 | KJ501399 | BID-V6657 | 2002 | USA |
| 197 | KJ501311 | BID-V6461 | 2002 | USA |
| 198 | KJ501387 | BID-V6642 | 2002 | USA |
| 199 | KJ501370 | BID-V6610 | 2002 | USA |
| 200 | KJ501501 | BID-V6538 | 2002 | USA |
| 201 | DQ164187 | NY_2002_Broome | 2002 | NY |
| 202 | KJ501315 | BID-V6472 | 2002 | USA |
| 203 | DQ080064 | TVP9222 | 2002 | Mexico |
| 204 | AY289214 | TVP 8533 | 2002 | TX |
| 205 | DQ164186 | NY 2002 Queens | 2002 | NY |
| 206 | KJ501347 | BID-V6543 | 2002 | USA |
| 207 | KJ501463 | BID-V6442 | 2002 | USA |
| 208 | KJ501482 | BID-V6491 | 2002 | USA |
| 209 | KJ501369 | BID-V6574 | 2002 | USA |
| 210 | KJ501462 | BID-V6441 | 2002 | USA |
| 211 | KJ501484 | BID-V6493 | 2002 | USA |
| 212 | KJ501483 | BID-V6492 | 2002 | USA |
| 213 | HM488137 | BID-V4202 | 2002 | CT |
| 214 | KJ501397 | BID-V6654 | 2002 | USA |
| 215 | KJ501384 | BID-V6635 | 2002 | USA |
| 216 | KJ501318 | BID-V6480 | 2002 | USA |
| 217 | KJ501337 | BID-V6518 | 2002 | USA |
| 218 | DQ080063 | TVP9223 | 2002 | Mexico |
| 219 | KJ501490 | BID-V6507 | 2002 | USA |
| 220 | KJ501321 | BID-V6484 | 2002 | USA |
| 221 | KJ501368 | BID-V6573 | 2002 | USA |
| 222 | KJ501319 | BID-V6481 | 2002 | USA |
| 223 | HM488181 | BID-V4340 | 2002 | IL |
| 224 | HM488182 | BID-V4341 | 2002 | IL |
| 225 | HM488180 | BID-V4339 | 2002 | IL |
| 226 | HM488177 | BID-V4336 | 2002 | IL |
| 227 | HM488178 | BID-V4337 | 2002 | IL |
| 228 | HM488179 | BID-V4338 | 2002 | IL |
| 229 | KJ501302 | BID-V6445 | 2002 | USA |
| 230 | KJ501322 | BID-V6485 | 2002 | USA |
| 231 | KJ501306 | BID-V6452 | 2002 | USA |
| 232 | KJ501339 | BID-V6521 | 2002 | USA |
| 233 | KJ501304 | BID-V6447 | 2002 | USA |
| 234 | KJ501303 | BID-V6446 | 2002 | USA |
| 235 | GU827998 | Bird114 | 2002 | TX |
| 236 | KJ501489 | BID-V6506 | 2002 | USA |
| 237 | KJ501450 | BID-V6399 | 2002 | USA |
| 238 | KJ501449 | BID-V6398 | 2002 | USA |
| 239 | KJ501345 | BID-V6539 | 2002 | USA |
| 240 | KJ501301 | BID-V6444 | 2002 | USA |
| 241 | KJ501338 | BID-V6519 | 2002 | USA |
| 242 | DQ080070 | TVP9115 | 2003 | Mexico |
| 243 | KJ501280 | BID-V6403 | 2003 | USA |
| 244 | JN051152 | TM171-03 pp1 | 2003 | Mexico |
| 245 | JN051153 | TM171-03 pp5 | 2003 | Mexico |
| 246 | DQ080058 | S0334814 | 2003 | CA |
| 247 | HQ671723 | BID-V4715 | 2003 | NY |
| 248 | DQ431694 | 03-22TX | 2003 | TX |
| 249 | DQ431697 | 03-113FL | 2003 | FL |
| 250 | DQ431698 | 03-120FL | 2003 | FL |
| 251 | DQ431695 | 03-82IL | 2003 | IL |
| 252 | DQ431696 | 03-104WI | 2003 | WI |
| 253 | DQ431693 | 03-20TX | 2003 | TX |
| 254 | HQ705660 | BID-V4714 | 2003 | NY |
| 255 | KJ501392 | BID-V6648 | 2003 | USA |
| 256 | KJ501391 | BID-V6647 | 2003 | USA |
| 257 | HQ705659 | BID-V4209 | 2003 | CT |
| 258 | KJ501356 | BID-V6559 | 2003 | USA |
| 259 | AY660002 | TM171-03 | 2003 | Mexico |
| 260 | KJ501390 | BID-V6646 | 2003 | USA |
| 261 | KJ501389 | BID-V6645 | 2003 | USA |
| 262 | KJ501396 | BID-V6653 | 2003 | USA |
| 263 | KJ501340 | BID-V6522 | 2003 | USA |
| 264 | DQ164199 | TX_2003 | 2003 | TX |
| 265 | KJ501327 | BID-V6490 | 2003 | USA |
| 266 | KJ501326 | BID-V6489 | 2003 | USA |
| 267 | KJ501359 | BID-V6563 | 2003 | USA |
| 268 | JF920728 | BID-V4568 | 2003 | CT |
| 269 | AY712947 | Bird 1461 | 2003 | TX |
| 270 | JN183890 | BID-V4699 | 2003 | CT |
| 271 | AY712946 | Bird 1171 | 2003 | TX |
| 272 | KJ501329 | BID-V6500 | 2003 | USA |
| 273 | KJ501103 | BID-V7391 | 2003 | CA |
| 274 | DQ164190 | NY_2003_Suffolk | 2003 | NY |
| 275 | DQ164192 | NY_2003_Rockland | 2003 | NY |
| 276 | DQ164191 | NY_2003_Chautauqua | 2003 | NY |
| 277 | KJ501104 | BID-V7392 | 2003 | CA |
| 278 | DQ164189 | NY_2003_Albany | 2003 | NY |
| 279 | KJ501105 | BID-V7393 | 2003 | CA |
| 280 | KJ501360 | BID-V6565 | 2003 | USA |
| 281 | AY712945 | Bird1153 | 2003 | TX |
| 282 | AY712948 | Mosquito v4369 | 2003 | TX |
| 283 | KJ501382 | BID-V6633 | 2003 | USA |
| 284 | JQ700437 | NY10-03 | 2003 | NY |
| 285 | KJ501460 | BID-V6439 | 2003 | USA |
| 286 | KJ501381 | BID-V6632 | 2003 | USA |
| 287 | DQ080060 | WNVCc | 2003 | Mexico |
| 288 | DQ080059 | L-CA-04_SAC-04-7168 | 2003 | CA |
| 289 | DQ164204 | CO_2003_1 | 2003 | CO |
| 290 | KJ501357 | BID-V6561 | 2003 | USA |
| 291 | KJ501358 | BID-V6562 | 2003 | USA |
| 292 | KJ501271 | BID-V6384 | 2003 | USA |
| 293 | JF703164 | CA-03 IMPR116 | 2003 | CA |
| 294 | JF920306 | BID-V4597 | 2003 | CT |
| 295 | KJ501107 | BID-V7395 | 2003 | CA |
| 296 | DQ164188 | NY_2003_Westchester | 2003 | NY |
| 297 | KJ501273 | BID-V6389 | 2003 | USA |
| 298 | DQ164203 | CO 2003 2 | 2003 | CO |
| 299 | JF703162 | CA-03 COAV997 | 2003 | CA |
| 300 | KJ501272 | BID-V6387 | 2003 | USA |
| 301 | DQ431699 | 03-124FL | 2003 | FL |
| 302 | KJ501506 | BID-V6553 | 2003 | USA |
| 303 | HM488187 | BID-V4351 | 2003 | IL |
| 304 | HM488186 | BID-V4350 | 2003 | IL |
| 305 | DQ080052 | B-AZ-03-1681 | 2003 | AZ |
| 306 | KJ501351 | BID-V6551 | 2003 | USA |
| 307 | DQ080053 | C-AZ-03_03-1799 | 2003 | AZ |
| 308 | KJ501508 | BID-V6560 | 2003 | USA |
| 309 | HM488185 | BID-V4347 | 2003 | IL |
| 310 | HM488172 | BID-V4561 | 2003 | CT |
| 311 | HM488171 | BID-V4560 | 2003 | CT |
| 312 | KJ501451 | BID-V6402 | 2003 | USA |
| 313 | HM488173 | BID-V4562 | 2003 | CT |
| 314 | HM488176 | BID-V4575 | 2003 | CT |
| 315 | HM488175 | BID-V4569 | 2003 | CT |
| 316 | HM488174 | BID-V4563 | 2003 | CT |
| 317 | HM488214 | BID-V4572 | 2003 | CT |
| 318 | HM488213 | BID-V4571 | 2003 | CT |
| 319 | HM488212 | BID-V4567 | 2003 | CT |
| 320 | HM488215 | BID-V4573 | 2003 | CT |
| 321 | HM488218 | BID-V4583 | 2003 | CT |
| 322 | HM488217 | BID-V4581 | 2003 | CT |
| 323 | HM488216 | BID-V4574 | 2003 | CT |
| 324 | HM488211 | BID-V4566 | 2003 | CT |
| 325 | KJ501323 | BID-V6486 | 2003 | USA |
| 326 | KJ501297 | BID-V6434 | 2003 | USA |
| 327 | KJ501352 | BID-V6552 | 2003 | USA |
| 328 | KJ501447 | BID-V6388 | 2003 | USA |
| 329 | HM488210 | BID-V4565 | 2003 | CT |
| 330 | HM488209 | BID-V4564 | 2003 | CT |
| 331 | KJ501446 | BID-V6386 | 2003 | USA |
| 332 | KJ501477 | BID-V6474 | 2003 | USA |
| 333 | KJ501313 | BID-V6466 | 2003 | USA |
| 334 | KJ501314 | BID-V6467 | 2003 | USA |
| 335 | KJ501365 | BID-V6570 | 2003 | USA |
| 336 | GQ507472 | 024WG-CA03OR | 2003 | CA |
| 337 | KJ501366 | BID-V6571 | 2003 | USA |
| 338 | KJ501488 | BID-V6499 | 2003 | USA |
| 339 | KJ501478 | BID-V6475 | 2003 | USA |
| 340 | GU828000 | Bird1175 | 2003 | TX |
| 341 | GU827999 | Bird1576 | 2003 | TX |
| 342 | KJ501320 | BID-V6482 | 2003 | USA |
| 343 | GU828001 | v4380 | 2003 | TX |
| 344 | GU828004 | Bird1519 | 2003 | TX |
| 345 | GU828003 | Bird1881 | 2003 | TX |
| 346 | GU828002 | v4095 | 2003 | TX |
| 347 | KJ501349 | BID-V6546 | 2003 | USA |
| 348 | KJ501348 | BID-V6544 | 2003 | USA |
| 349 | KJ501346 | BID-V6541 | 2003 | USA |
| 350 | KJ501312 | BID-V6464 | 2003 | USA |
| 351 | KJ501504 | BID-V6548 | 2003 | USA |
| 352 | KJ501305 | BID-V6449 | 2003 | USA |
| 353 | KJ501324 | BID-V6487 | 2003 | USA |
| 354 | KJ501499 | BID-V6535 | 2003 | USA |
| 355 | HM488140 | BID-V4210 | 2003 | CT |
| 356 | HM488139 | BID-V4208 | 2003 | CT |
| 357 | HM488138 | BID-V4207 | 2003 | CT |
| 358 | HM488141 | BID-V4212 | 2003 | CT |
| 359 | KJ501498 | BID-V6534 | 2003 | USA |
| 360 | DQ080051 | A-AZ-03-1623 | 2003 | AZ |
| 361 | DQ005530 | BSL5-2003 | 2003 | UT |
| 362 | HM488220 | BID-V4586 | 2003 | CT |
| 363 | KJ501472 | BID-V6468 | 2003 | USA |
| 364 | DQ080056 | G-CA-03_IMPR-1075 | 2003 | CA |
| 365 | HM756659 | BID-V4687 | 2003 | CT |
| 366 | HM756666 | BID-V4711 | 2003 | NY |
| 367 | HM756669 | BID-V4718 | 2003 | NY |
| 368 | HM756668 | BID-V4716 | 2003 | NY |
| 369 | HM756667 | BID-V4712 | 2003 | NY |
| 370 | HM756653 | BID-V4588 | 2003 | CT |
| 371 | HM756652 | BID-V4587 | 2003 | CT |
| 372 | HM756651 | BID-V4584 | 2003 | CT |
| 373 | HM756654 | BID-V4598 | 2003 | CT |
| 374 | HM756658 | BID-V4686 | 2003 | CT |
| 375 | HM756657 | BID-V4685 | 2003 | CT |
| 376 | HM756656 | BID-V4615 | 2003 | CT |
| 377 | HQ671704 | BID-V4618 | 2003 | CT |
| 378 | HQ671703 | BID-V4611 | 2003 | CT |
| 379 | HQ671702 | BID-V4595 | 2003 | CT |
| 380 | HQ671705 | BID-V4620 | 2003 | CT |
| 381 | KJ501372 | BID-V6615 | 2003 | USA |
| 382 | KJ501527 | BID-V6696 | 2003 | USA |
| 383 | DQ080057 | S0331532 | 2003 | CA |
| 384 | HM756676 | BID-V4349 | 2003 | IL |
| 385 | KJ501354 | BID-V6555 | 2003 | USA |
| 386 | HM756670 | BID-V4720 | 2003 | NY |
| 387 | KJ501470 | BID-V6463 | 2003 | USA |
| 388 | HQ671701 | BID-V4590 | 2003 | CT |
| 389 | HQ671700 | BID-V4576 | 2003 | CT |
| 390 | KJ501469 | BID-V6462 | 2003 | USA |
| 391 | HM756650 | BID-V4582 | 2003 | CT |
| 392 | HM488233 | BID-V4616 | 2003 | CT |
| 393 | HM488232 | BID-V4614 | 2003 | CT |
| 394 | HM488231 | BID-V4613 | 2003 | CT |
| 395 | HM488235 | BID-V4619 | 2003 | CT |
| 396 | HM488234 | BID-V4617 | 2003 | CT |
| 397 | HM488223 | BID-V4603 | 2003 | CT |
| 398 | HM488230 | BID-V4612 | 2003 | CT |
| 399 | HM488226 | BID-V4607 | 2003 | CT |
| 400 | HM488225 | BID-V4605 | 2003 | CT |
| 401 | HM488224 | BID-V4604 | 2003 | CT |
| 402 | HM488229 | BID-V4610 | 2003 | CT |
| 403 | HM488228 | BID-V4609 | 2003 | CT |
| 404 | HM488227 | BID-V4608 | 2003 | CT |
| 405 | KJ501353 | BID-V6554 | 2003 | USA |
| 406 | DQ080054 | E-CA-03_GRLA-1260 | 2003 | CA |
| 407 | EU155484 | OK03 | 2003 | OK |
| 408 | HM488250 | BID-V4717 | 2003 | NY |
| 409 | HM488221 | BID-V4593 | 2003 | CT |
| 410 | DQ080055 | F-CA-03_IMPR_102 | 2003 | CA |
| 411 | HM488251 | BID-V4719 | 2003 | NY |
| 412 | HM488222 | BID-V4599 | 2003 | CT |
| 413 | KJ501456 | BID-V6426 | 2003 | USA |
| 414 | HM488236 | BID-V4700 | 2003 | CT |
| 415 | KJ501293 | BID-V6427 | 2003 | USA |
| 416 | KJ501294 | BID-V6428 | 2003 | USA |
| 417 | KJ501457 | BID-V6429 | 2003 | USA |
| 418 | KJ501525 | BID-V6689 | 2004 | USA |
| 419 | JN367277 | BID-V4803 | 2004 | NY |
| 420 | DQ080061 | TWN496 | 2004 | LA |
| 421 | KJ501522 | BID-V6676 | 2004 | USA |
| 422 | KJ501520 | BID-V6661 | 2004 | USA |
| 423 | DQ666448 | BSL5-2004 | 2004 | AZ |
| 424 | DQ431712 | 04-252AZ | 2004 | AZ |
| 425 | DQ431711 | 04-251AZ | 2004 | AZ |
| 426 | KJ501123 | BID-V7411 | 2004 | CA |
| 427 | HM488191 | BID-V4369 | 2004 | IL |
| 428 | KJ501128 | BID-V7417 | 2004 | CA |
| 429 | KJ501125 | BID-V7414 | 2004 | CA |
| 430 | HM756673 | BID-V4801 | 2004 | NY |
| 431 | DQ431708 | 04-238CA | 2004 | CA |
| 432 | DQ431707 | 04-237NM | 2004 | NM |
| 433 | HM756672 | BID-V4799 | 2004 | NY |
| 434 | DQ431710 | 04-244CA | 2004 | CA |
| 435 | DQ431709 | 04-240CA | 2004 | CA |
| 436 | HM756671 | BID-V4798 | 2004 | NY |
| 437 | HM488144 | BID-V4216 | 2004 | CT |
| 438 | HM488145 | BID-V4217 | 2004 | CT |
| 439 | HM488146 | BID-V4218 | 2004 | CT |
| 440 | HM488143 | BID-V4215 | 2004 | CT |
| 441 | GQ507474 | 091WG-CA04SB | 2004 | CA |
| 442 | GQ507473 | 080WG-CA04LA | 2004 | CA |
| 443 | HM488142 | BID-V4214 | 2004 | CT |
| 444 | HM488188 | BID-V4353 | 2004 | IL |
| 445 | HM488189 | BID-V4367 | 2004 | IL |
| 446 | HM488190 | BID-V4368 | 2004 | IL |
| 447 | KJ501129 | BID-V7418 | 2004 | CA |
| 448 | HM488147 | BID-V4219 | 2004 | CT |
| 449 | HM488148 | BID-V4220 | 2004 | CT |
| 450 | KJ501131 | BID-V7420 | 2004 | CA |
| 451 | JF488089 | BID-V5179 | 2004 | CT |
| 452 | JF488090 | BID-V5180 | 2004 | CT |
| 453 | JF488091 | BID-V5181 | 2004 | CT |
| 454 | JF488088 | BID-V5178 | 2004 | CT |
| 455 | KJ501419 | BID-V6686 | 2004 | USA |
| 456 | JF488086 | BID-V5176 | 2004 | CT |
| 457 | JF488087 | BID-V5177 | 2004 | CT |
| 458 | JF488092 | BID-V5182 | 2004 | CT |
| 459 | KJ501402 | BID-V6662 | 2004 | USA |
| 460 | KJ501398 | BID-V6656 | 2004 | USA |
| 461 | KJ501385 | BID-V6637 | 2004 | USA |
| 462 | DQ164201 | AZ_2004 | 2004 | AZ |
| 463 | JF488094 | BID-V5150 | 2004 | NY |
| 464 | JF703161 | CA-04 COAV689 | 2004 | CA |
| 465 | JF899528 | BID-V4800 | 2004 | NY |
| 466 | DQ431700 | 04-213CA | 2004 | CA |
| 467 | DQ431704 | 04-219CO | 2004 | CO |
| 468 | DQ164206 | TX_2004 | 2004 | TX |
| 469 | DQ431703 | 04-218CO | 2004 | CO |
| 470 | DQ431702 | 04-216CO | 2004 | CO |
| 471 | DQ431701 | 04-214CO | 2004 | CO |
| 472 | DQ431705 | 04-233ND | 2004 | ND |
| 473 | KJ501422 | BID-V6690 | 2004 | USA |
| 474 | KJ501421 | BID-V6688 | 2004 | USA |
| 475 | KJ501420 | BID-V6687 | 2004 | USA |
| 476 | KJ501423 | BID-V6691 | 2004 | USA |
| 477 | DQ431706 | 04-236NM | 2004 | NM |
| 478 | KJ501487 | BID-V6497 | 2005 | USA |
| 479 | KJ501119 | BID-V7407 | 2005 | CA |
| 480 | KJ501492 | BID-V6512 | 2005 | USA |
| 481 | KJ501115 | BID-V7403 | 2005 | CA |
| 482 | KJ501186 | BID-V7484 | 2005 | CA |
| 483 | HM488152 | BID-V4226 | 2005 | CT |
| 484 | HM488153 | BID-V4227 | 2005 | CT |
| 485 | HM488154 | BID-V4228 | 2005 | CT |
| 486 | HM488149 | BID-V4223 | 2005 | CT |
| 487 | HM488150 | BID-V4224 | 2005 | CT |
| 488 | HM488151 | BID-V4225 | 2005 | CT |
| 489 | GQ507468 | 007WG-TX05EP | 2005 | TX |
| 490 | HM488196 | BID-V4376 | 2005 | IL |
| 491 | HM488197 | BID-V4377 | 2005 | IL |
| 492 | HM488198 | BID-V4378 | 2005 | IL |
| 493 | HM488192 | BID-V4371 | 2005 | IL |
| 494 | HM488193 | BID-V4373 | 2005 | IL |
| 495 | HM488195 | BID-V4375 | 2005 | IL |
| 496 | GQ507469 | 009WG-NM05LC | 2005 | NM |
| 497 | HM488118 | BID-V4107 | 2005 | CT |
| 498 | HM488119 | BID-V4108 | 2005 | CT |
| 499 | HM488120 | BID-V4109 | 2005 | CT |
| 500 | HM488115 | BID-V4103 | 2005 | CT |
| 501 | HM488116 | BID-V4104 | 2005 | CT |
| 502 | HM488117 | BID-V4105 | 2005 | CT |
| 503 | HM488121 | BID-V4110 | 2005 | CT |
| 504 | GQ507477 | 103WG-CA05LA | 2005 | CA |
| 505 | GQ507476 | 101WG-CA05SB | 2005 | CA |
| 506 | GQ507475 | 099WG-CA05SB | 2005 | CA |
| 507 | GQ507480 | 132WG-CA05LA | 2005 | CA |
| 508 | GQ507479 | 124WG-AZ05PI | 2005 | AZ |
| 509 | GQ507478 | 116WG-CA05LA | 2005 | CA |
| 510 | DQ666452 | BSL2-2005 | 2005 | SD |
| 511 | KJ501414 | BID-V6678 | 2005 | USA |
| 512 | KJ501404 | BID-V6664 | 2005 | USA |
| 513 | KJ501403 | BID-V6663 | 2005 | USA |
| 514 | KJ501267 | BID-V6378 | 2005 | USA |
| 515 | KJ501330 | BID-V6501 | 2005 | USA |
| 516 | KJ501335 | BID-V6513 | 2005 | USA |
| 517 | KJ501341 | BID-V6523 | 2005 | USA |
| 518 | JX015515 | TX_AR5-2686 | 2005 | TX |
| 519 | KJ501471 | BID-V6465 | 2005 | USA |
| 520 | KJ145799 | BID-V4106 | 2005 | CT |
| 521 | KJ501342 | BID-V6524 | 2005 | USA |
| 522 | KJ501461 | BID-V6440 | 2005 | USA |
| 523 | JN183892 | BID-V4379 | 2005 | IL |
| 524 | JF899529 | BID-V4808 | 2005 | NY |
| 525 | HM488252 | BID-V4805 | 2005 | NY |
| 526 | HM756675 | BID-V4806 | 2005 | NY |
| 527 | HM756677 | BID-V4530 | 2005 | NM |
| 528 | DQ666451 | BSL13-2005 | 2005 | AZ |
| 529 | DQ666450 | GCTX2-2005 | 2005 | TX |
| 530 | DQ666449 | GCTX1-2005 | 2005 | TX |
| 531 | HQ671724 | BID-V4883 | 2005 | NY |
| 532 | JF415929 | TX5058 | 2005 | TX |
| 533 | JF488093 | BID-V5188 | 2005 | CT |
| 534 | JF703163 | CA-05 COAV2900 | 2005 | CA |
| 535 | HQ671725 | BID-V4885 | 2005 | NY |
| 536 | HQ671726 | BID-V4887 | 2005 | NY |
| 537 | JF415914 | M12214 | 2005 | TX |
| 538 | JF415930 | M6019 | 2006 | TX |
| 539 | JF415916 | TX6276 | 2006 | TX |
| 540 | JF920729 | BID-V5196 | 2006 | CT |
| 541 | KJ501416 | BID-V6683 | 2006 | USA |
| 542 | HQ671728 | BID-V4891 | 2006 | NY |
| 543 | HQ671727 | BID-V4889 | 2006 | NY |
| 544 | JF415915 | TX5810 | 2006 | TX |
| 545 | HQ671729 | BID-V4892 | 2006 | NY |
| 546 | JF920730 | BID-V5197 | 2006 | CT |
| 547 | JF920736 | BID-V5206 | 2006 | CT |
| 548 | JF920735 | BID-V5205 | 2006 | CT |
| 549 | KJ501415 | BID-V6681 | 2006 | USA |
| 550 | JF920737 | BID-V5207 | 2006 | CT |
| 551 | JF920732 | BID-V5202 | 2006 | CT |
| 552 | JF920731 | BID-V5201 | 2006 | CT |
| 553 | JF920734 | BID-V5204 | 2006 | CT |
| 554 | JF920733 | BID-V5203 | 2006 | CT |
| 555 | KJ501428 | BID-V6700 | 2006 | USA |
| 556 | HM488124 | BID-V4113 | 2006 | CT |
| 557 | HM488123 | BID-V4112 | 2006 | CT |
| 558 | HM488156 | BID-V4230 | 2006 | CT |
| 559 | HM488155 | BID-V4229 | 2006 | CT |
| 560 | GQ507481 | 142WG-NE06DO | 2006 | NE |
| 561 | GQ507470 | 011WG-TX06EP | 2006 | TX |
| 562 | HM488122 | BID-V4111 | 2006 | CT |
| 563 | GQ507482 | 144WG-AZ06PI | 2006 | AZ |
| 564 | HM488157 | BID-V4231 | 2006 | CT |
| 565 | KJ501429 | BID-V6702 | 2006 | USA |
| 566 | KJ501430 | BID-V6703 | 2006 | USA |
| 567 | HM756649 | BID-V4354 | 2006 | CT |
| 568 | HM488253 | BID-V4553 | 2006 | IL |
| 569 | HM488159 | BID-V4233 | 2006 | CT |
| 570 | HM488158 | BID-V4232 | 2006 | CT |
| 571 | KJ501431 | BID-V6704 | 2006 | USA |
| 572 | HM488160 | BID-V4355 | 2006 | CT |
| 573 | KJ501410 | BID-V6670 | 2006 | USA |
| 574 | JF957163 | ARC17-06 | 2006 | ID |
| 575 | JF957164 | ARC23-06 | 2006 | ID |
| 576 | JF957165 | ARC27-06 | 2006 | ID |
| 577 | JF957162 | ARC13-06 | 2006 | ID |
| 578 | KJ501406 | BID-V6666 | 2006 | USA |
| 579 | KJ501405 | BID-V6665 | 2006 | USA |
| 580 | JF957161 | ARC10-06 | 2006 | ID |
| 581 | JF957166 | ARC33-06 | 2006 | UT |
| 582 | KJ501526 | BID-V6692 | 2006 | USA |
| 583 | KJ501529 | BID-V6701 | 2006 | USA |
| 584 | JN183888 | BID-V4896 | 2006 | NY |
| 585 | KJ501524 | BID-V6682 | 2006 | USA |
| 586 | KJ501521 | BID-V6672 | 2006 | USA |
| 587 | JF957167 | BSL106-06 | 2006 | ND |
| 588 | KJ501523 | BID-V6680 | 2006 | USA |
| 589 | KJ501485 | BID-V6495 | 2006 | USA |
| 590 | KJ501409 | BID-V6669 | 2006 | USA |
| 591 | KJ501408 | BID-V6668 | 2006 | USA |
| 592 | KJ501407 | BID-V6667 | 2006 | USA |
| 593 | HM488199 | BID-V4090 | 2007 | NY |
| 594 | HM488200 | BID-V4092 | 2007 | NY |
| 595 | HM488165 | BID-V4361 | 2007 | CT |
| 596 | HM488164 | BID-V4360 | 2007 | CT |
| 597 | HM488163 | BID-V4359 | 2007 | CT |
| 598 | KJ501168 | BID-V7463 | 2007 | CA |
| 599 | GQ379158 | ORCO0559-07 | 2007 | CA |
| 600 | JX015516 | TX_AR7-6745 | 2007 | TX |
| 601 | HM488201 | BID-V4093 | 2007 | NY |
| 602 | HM488202 | BID-V4094 | 2007 | NY |
| 603 | GQ507471 | 013WG-TX07EP | 2007 | TX |
| 604 | KJ501126 | BID-V7415 | 2007 | CA |
| 605 | KJ501195 | BID-V7494 | 2007 | CA |
| 606 | KJ145832 | BID-V4529 | 2007 | NM |
| 607 | KJ501108 | BID-V7396 | 2007 | CA |
| 608 | KJ501112 | BID-V7400 | 2007 | CA |
| 609 | KJ501114 | BID-V7402 | 2007 | CA |
| 610 | KJ501132 | BID-V7421 | 2007 | CA |
| 611 | HM488161 | BID-V4356 | 2007 | CT |
| 612 | HM488162 | BID-V4357 | 2007 | CT |
| 613 | KJ501518 | BID-V6640 | 2007 | USA |
| 614 | GQ507484 | 149WG-CA07LA | 2007 | CA |
| 615 | GQ507483 | 148WG-CA07LA | 2007 | CA |
| 616 | JF920738 | BID-V5208 | 2007 | CT |
| 617 | JF920739 | BID-V5209 | 2007 | CT |
| 618 | KJ501258 | BID-V6209 | 2007 | USA |
| 619 | JF730042 | BID-V5147 | 2007 | NY |
| 620 | KJ501425 | BID-V6695 | 2007 | USA |
| 621 | KJ501424 | BID-V6694 | 2007 | USA |
| 622 | JF488097 | BID-V5148 | 2007 | NY |
| 623 | JF920744 | BID-V5215 | 2007 | CT |
| 624 | JF920745 | BID-V5216 | 2007 | CT |
| 625 | JF920746 | BID-V5217 | 2007 | CT |
| 626 | JF920743 | BID-V5214 | 2007 | CT |
| 627 | JF920740 | BID-V5210 | 2007 | CT |
| 628 | JF920741 | BID-V5212 | 2007 | CT |
| 629 | JF920742 | BID-V5213 | 2007 | CT |
| 630 | JF957172 | ID28_bird | 2007 | ID |
| 631 | JF957171 | ID21_bird | 2007 | ID |
| 632 | JF957170 | CO5-07 | 2007 | CO |
| 633 | HM488254 | BID-V4559 | 2007 | IL |
| 634 | KJ501386 | BID-V6639 | 2007 | USA |
| 635 | HM756678 | BID-V4095 | 2007 | NY |
| 636 | JF957169 | CO4-07 | 2007 | CO |
| 637 | JF415918 | TX6747 | 2007 | TX |
| 638 | JF415919 | M19433 | 2007 | TX |
| 639 | JF415920 | TX7191 | 2007 | TX |
| 640 | JF415917 | TX6647 | 2007 | TX |
| 641 | JF957168 | ARC140-07 | 2007 | ID |
| 642 | KJ501412 | BID-V6674 | 2008 | USA |
| 643 | KJ501411 | BID-V6673 | 2008 | USA |
| 644 | KJ501440 | BID-V6212 | 2008 | USA |
| 645 | KJ501418 | BID-V6685 | 2008 | USA |
| 646 | KJ501441 | BID-V6213 | 2008 | USA |
| 647 | JF920747 | BID-V5218 | 2008 | CT |
| 648 | HM488237 | BID-V4622 | 2008 | NY |
| 649 | JF920754 | BID-V5226 | 2008 | CT |
| 650 | HM488238 | BID-V4623 | 2008 | NY |
| 651 | HM488240 | BID-V4627 | 2008 | NY |
| 652 | JF920748 | BID-V5219 | 2008 | CT |
| 653 | HM488239 | BID-V4624 | 2008 | NY |
| 654 | HM488207 | BID-V4101 | 2008 | NY |
| 655 | JF920757 | BID-V5230 | 2008 | CT |
| 656 | KJ501106 | BID-V7394 | 2008 | CA |
| 657 | KJ501109 | BID-V7397 | 2008 | CA |
| 658 | JF920756 | BID-V5229 | 2008 | CT |
| 659 | HM488206 | BID-V4100 | 2008 | NY |
| 660 | HM488205 | BID-V4099 | 2008 | NY |
| 661 | JF920755 | BID-V5227 | 2008 | CT |
| 662 | JF920749 | BID-V5220 | 2008 | CT |
| 663 | JF972636 | BID-V5228 | 2008 | CT |
| 664 | JN183885 | BID-V4626 | 2008 | NY |
| 665 | JN183886 | BID-V4629 | 2008 | NY |
| 666 | HM488245 | BID-V4635 | 2008 | NY |
| 667 | JF920753 | BID-V5225 | 2008 | CT |
| 668 | JF920752 | BID-V5224 | 2008 | CT |
| 669 | HM756660 | BID-V4097 | 2008 | NY |
| 670 | HM488244 | BID-V4634 | 2008 | NY |
| 671 | JX015518 | TX_AR8-6866 | 2008 | Mexico |
| 672 | JF920751 | BID-V5223 | 2008 | CT |
| 673 | JF920750 | BID-V5222 | 2008 | CT |
| 674 | JX015517 | TX_AR8-5947 | 2008 | TX |
| 675 | HM488243 | BID-V4632 | 2008 | NY |
| 676 | HM488242 | BID-V4631 | 2008 | NY |
| 677 | HM488241 | BID-V4628 | 2008 | NY |
| 678 | KJ501240 | BID-V6183 | 2008 | USA |
| 679 | JF957173 | BSL173-08 | 2008 | AZ |
| 680 | HM488166 | BID-V4362 | 2008 | CT |
| 681 | KJ501239 | BID-V6182 | 2008 | USA |
| 682 | KJ501260 | BID-V6211 | 2008 | USA |
| 683 | KJ501237 | BID-V6178 | 2008 | USA |
| 684 | KJ501238 | BID-V6179 | 2008 | USA |
| 685 | KJ501255 | BID-V6204 | 2008 | USA |
| 686 | HQ671721 | BID-V4625 | 2008 | NY |
| 687 | KJ501259 | BID-V6210 | 2008 | USA |
| 688 | KJ501254 | BID-V6203 | 2008 | USA |
| 689 | JF957174 | BSL176-08 | 2008 | NV |
| 690 | JF415921 | TX7558 | 2008 | TX |
| 691 | KJ501253 | BID-V6200 | 2008 | USA |
| 692 | KJ501206 | BID-V7384 | 2008 | CA |
| 693 | HM488204 | BID-V4098 | 2008 | NY |
| 694 | HM488203 | BID-V4096 | 2008 | NY |
| 695 | HM488170 | BID-V4366 | 2008 | CT |
| 696 | KJ501116 | BID-V7404 | 2008 | CA |
| 697 | GQ379157 | DB080718-14 | 2008 | CA |
| 698 | KJ501113 | BID-V7401 | 2008 | CA |
| 699 | GQ379159 | JPW080813-01 | 2008 | CA |
| 700 | HM488168 | BID-V4364 | 2008 | CT |
| 701 | HM488167 | BID-V4363 | 2008 | CT |
| 702 | KJ501147 | BID-V7437 | 2008 | CA |
| 703 | HM488169 | BID-V4365 | 2008 | CT |
| 704 | KJ501127 | BID-V7416 | 2008 | CA |
| 705 | KJ501130 | BID-V7419 | 2008 | CA |
| 706 | KJ501133 | BID-V7422 | 2008 | CA |
| 707 | JF920759 | BID-V5234 | 2009 | CT |
| 708 | JF920758 | BID-V5233 | 2009 | CT |
| 709 | JF920760 | BID-V5235 | 2009 | CT |
| 710 | JF415924 | TX7827 | 2009 | TX |
| 711 | JF415925 | M38488 | 2009 | TX |
| 712 | JF415922 | M37012 | 2009 | TX |
| 713 | JF415923 | M37906 | 2009 | TX |
| 714 | JF415926 | M20140 | 2009 | TX |
| 715 | JF488095 | BID-V5157 | 2009 | NY |
| 716 | JF488096 | BID-V5159 | 2009 | NY |
| 717 | JF415927 | M20141 | 2009 | TX |
| 718 | JF415928 | M20122 | 2009 | TX |
| 719 | KJ501100 | BID-V7388 | 2009 | CA |
| 720 | KJ501101 | BID-V7389 | 2009 | CA |
| 721 | KJ501102 | BID-V7390 | 2009 | CA |
| 722 | JX015521 | TX_AR9-7465 | 2009 | TX |
| 723 | KJ501500 | BID-V6536 | 2009 | USA |
| 724 | KJ501099 | BID-V7387 | 2009 | CA |
| 725 | KJ501250 | BID-V6196 | 2009 | USA |
| 726 | KJ501251 | BID-V6197 | 2009 | USA |
| 727 | KJ501256 | BID-V6207 | 2009 | USA |
| 728 | KJ501110 | BID-V7398 | 2009 | CA |
| 729 | KJ501236 | BID-V6177 | 2009 | USA |
| 730 | KJ501241 | BID-V6184 | 2009 | USA |
| 731 | JF957179 | BSL18-09 | 2009 | LA |
| 732 | JF957180 | BSL20-09 | 2009 | NV |
| 733 | JF957181 | BSL22-09 | 2009 | SD |
| 734 | JF957178 | BSL11-09 | 2009 | NV |
| 735 | JF957175 | BSL2-09 | 2009 | NV |
| 736 | JF957176 | BSL5-09 | 2009 | AZ |
| 737 | JF957177 | BSL6-09 | 2009 | NV |
| 738 | JX015520 | TX_AR9-6115 | 2009 | Mexico |
| 739 | JX015519 | TX_AR9-5282 | 2009 | TX |
| 740 | JF957184 | CO7-09 | 2009 | CO |
| 741 | JF957182 | BSL24-09 | 2009 | TX |
| 742 | JF957183 | BSL27-09 | 2009 | TX |
| 743 | KJ501438 | BID-V6171 | 2010 | USA |
| 744 | KJ501442 | BID-V6371 | 2010 | USA |
| 745 | KJ501117 | BID-V7405 | 2010 | CA |
| 746 | KJ501120 | BID-V7408 | 2010 | CA |
| 747 | KJ501124 | BID-V7412 | 2010 | CA |
| 748 | KF704158 | AZ10-892 | 2010 | AZ |
| 749 | KJ145827 | BID-V6056 | 2010 | NY |
| 750 | KJ145828 | BID-V6057 | 2010 | NY |
| 751 | KJ501242 | BID-V6185 | 2010 | USA |
| 752 | KJ501262 | BID-V6215 | 2010 | USA |
| 753 | KJ501308 | BID-V6456 | 2010 | USA |
| 754 | KJ501134 | BID-V7423 | 2010 | CA |
| 755 | KJ501135 | BID-V7425 | 2010 | CA |
| 756 | KJ501136 | BID-V7426 | 2010 | CA |
| 757 | JX015522 | TX_AR10-5718 | 2010 | TX |
| 758 | JX015523 | TX_AR10-6572 | 2010 | TX |
| 759 | JF957185 | BSL2-10 | 2010 | AZ |
| 760 | JF957186 | BSL3-10 | 2010 | AZ |
| 761 | KF704147 | AZ10-91 | 2010 | AZ |
| 762 | KF704153 | AZ10-581 | 2010 | AZ |
| 763 | KJ501538 | BID-V7822 | 2011 | NY |
| 764 | KJ501539 | BID-V7823 | 2011 | NY |
| 765 | KJ501534 | BID-V7818 | 2011 | NY |
| 766 | KJ501535 | BID-V7819 | 2011 | NY |
| 767 | KJ501537 | BID-V7821 | 2011 | NY |
| 768 | KJ501536 | BID-V7820 | 2011 | NY |
| 769 | KJ501140 | BID-V7430 | 2011 | CA |
| 770 | KJ501141 | BID-V7431 | 2011 | CA |
| 771 | KJ501139 | BID-V7429 | 2011 | CA |
| 772 | KJ501137 | BID-V7427 | 2011 | CA |
| 773 | KJ501138 | BID-V7428 | 2011 | CA |
| 774 | KJ501142 | BID-V7432 | 2011 | CA |
| 775 | KJ501148 | BID-V7438 | 2011 | CA |
| 776 | KJ501149 | BID-V7441 | 2011 | CA |
| 777 | KJ501146 | BID-V7436 | 2011 | CA |
| 778 | KJ501143 | BID-V7433 | 2011 | CA |
| 779 | KJ501144 | BID-V7434 | 2011 | CA |
| 780 | JQ700440 | BSL23-11 | 2011 | AZ |
| 781 | JQ700439 | BSL6-11 | 2011 | MS |
| 782 | JQ700441 | BSL24-11 | 2011 | CA |
| 783 | KC333374 | TX8092 | 2011 | TX |
| 784 | JQ700442 | BSL26-11 | 2011 | NY |
| 785 | JQ700438 | BSL4-11 | 2011 | AZ |
| 786 | KJ501121 | BID-V7409 | 2011 | CA |
| 787 | KJ501122 | BID-V7410 | 2011 | CA |
| 788 | KJ501118 | BID-V7406 | 2011 | CA |
| 789 | KJ501096 | BID-V7383 | 2011 | CA |
| 790 | KJ501098 | BID-V7386 | 2011 | CA |
| 791 | KJ501201 | BID-V7507 | 2011 | CA |
| 792 | KJ501170 | BID-V7465 | 2011 | CA |
| 793 | KJ501150 | BID-V7442 | 2011 | CA |
| 794 | KC333375 | TX8349 | 2012 | TX |
| 795 | KC333376 | TX8546 | 2012 | TX |
| 796 | KC333377 | TX8551 | 2012 | TX |
| 797 | KC333378 | TX8559 | 2012 | TX |
| 798 | KC333379 | TX8560 | 2012 | TX |
| 799 | KC333380 | TX8562 | 2012 | TX |
| 800 | KC333381 | TX8567 | 2012 | TX |
| 801 | KC333382 | TX8571 | 2012 | TX |
| 802 | KC333383 | TX8572 | 2012 | TX |
| 803 | KC333384 | TX8589 | 2012 | TX |
| 804 | KC333385 | TX8590 | 2012 | TX |
| 805 | KC333386 | TX8599 | 2012 | TX |
| 806 | KC333387 | TX8604 | 2012 | TX |
| 807 | KC711057 | TX_AR12-1486 | 2012 | TX |
| 808 | KC711058 | TX_AR12-1648 | 2012 | TX |
| 809 | KC711059 | TX_AR12-10674 | 2012 | TX |
| 810 | KC736486 | AVA1202598 | 2012 | TX |
| 811 | KC736487 | AVA1202600 | 2012 | TX |
| 812 | KC736488 | AVA1202606 | 2012 | TX |
| 813 | KC736489 | AVA1202615 | 2012 | TX |
| 814 | KC736490 | AVA1202621 | 2012 | TX |
| 815 | KC736491 | AVA1202624 | 2012 | TX |
| 816 | KC736492 | AVA1202689 | 2012 | TX |
| 817 | KC736493 | AVA1202696 | 2012 | TX |
| 818 | KC736494 | AVA1204250 | 2012 | TX |
| 819 | KC736495 | AVA1204331 | 2012 | TX |
| 820 | KC736496 | AVA1204356 | 2012 | TX |
| 821 | KC736497 | AVA1204485 | 2012 | TX |
| 822 | KC736498 | AVA1204579 | 2012 | TX |
| 823 | KC736499 | AVA1204580 | 2012 | TX |
| 824 | KC736500 | AVA1204753 | 2012 | TX |
| 825 | KC736501 | AVA1204895 | 2012 | TX |
| 826 | KC736502 | AVA1204260 | 2012 | TX |
| 827 | KJ501207 | BID-V6414 | 2012 | CO |
| 828 | KJ501208 | BID-V6578 | 2012 | CO |
| 829 | KJ501211 | BID-V6584 | 2012 | CO |
| 830 | KJ501214 | BID-V6589 | 2012 | CO |
| 831 | KJ501215 | BID-V6592 | 2012 | CO |
| 832 | KJ501216 | BID-V6594 | 2012 | CO |
| 833 | KJ501217 | BID-V6597 | 2012 | CO |
| 834 | KJ501218 | BID-V6598 | 2012 | CO |
| 835 | KJ501219 | BID-V6600 | 2012 | CO |
| 836 | KJ501220 | BID-V6602 | 2012 | CO |
| 837 | KJ501222 | BID-V7773 | 2012 | CO |
| 838 | KJ501224 | BID-V7788 | 2012 | CO |
| 839 | KJ501225 | BID-V7789 | 2012 | TX |
| 840 | KJ501226 | BID-V7791 | 2012 | CO |
| 841 | KJ501229 | BID-V7795 | 2012 | CO |
| 842 | KJ501230 | BID-V7790 | 2012 | CO |
| 843 | KJ501432 | BID-V7270 | 2012 | USA |
| 844 | KJ501434 | BID-V7277 | 2012 | USA |
| 845 | KJ501437 | BID-V7286 | 2012 | USA |
| 846 | KJ501530 | BID-V7281 | 2012 | USA |
| 847 | KJ501531 | BID-V7815 | 2012 | NY |
| 848 | KJ501532 | BID-V7816 | 2012 | NY |
| 849 | KJ501533 | BID-V7817 | 2012 | NY |
| 850 | KJ786935 | TX8779 | 2012 | TX |
| 851 | KJ786936 | TX8759 | 2012 | TX |
| 852 | KM012170 | BSL05 | 2012 | AZ |
| 853 | KM012171 | BSL08 | 2012 | MS |
| 854 | KM012172 | BSL53 | 2012 | TX |
| 855 | KM012173 | BSL78 | 2012 | SD |
| 856 | KM012174 | BSL80 | 2012 | AZ |
| 857 | KM012175 | BSL85 | 2012 | CA |
| 858 | KM012176 | BSL93 | 2012 | ND |
| 859 | KM012177 | BSL101 | 2012 | WY |
| 860 | KM012178 | BSL107 | 2012 | TX |
| 861 | KM012179 | BSL116 | 2012 | SD |
| 862 | KM012180 | BSL140 | 2012 | GA |
| 863 | KM012181 | BSL178 | 2012 | LA |
| 864 | KM012182 | BSL195 | 2012 | NM |
| 865 | KM012183 | BSL221 | 2012 | TX |
| 866 | KM012184 | ARC1 | 2012 | OH |
| 867 | KM012185 | ARC3 | 2012 | NE |
| 868 | KM012186 | ARC4 | 2012 | OH |
| 869 | KM012187 | ARC6 | 2012 | NE |
| 870 | KM012188 | ARC13 | 2012 | IL |
