# Supplementary material for: Genetic Variability of West Nile Virus in U.S. Blood Donors from the 2012 Epidemic Season
Source: PLoS Negl Trop Dis. 2016 May 16;10(5):e0004717. doi: 10.1371/journal.pntd.0004717 (PMC4868353; doi:10.1371/journal.pntd.0004717)
Supplement: S2 Table — (DOCX) [file pntd.0004717.s002.docx]

Supplemental Table 2. Nucleotide mutations present in 2012 human WNV isolates, compared to the prototype strain WN-NY99 (AF196835).

| Gene | 5'UTR | | core | | | | | | | | | | | | | | | | | prM | | | | | | | | | | | | | | | | | | | | | | | Env | | | | | | | | | |
| --- | --- | --- | --- | --- | --- | --- | --- | --- | --- | --- | --- | --- | --- | --- | --- | --- | --- | --- | --- | --- | --- | --- | --- | --- | --- | --- | --- | --- | --- | --- | --- | --- | --- | --- | --- | --- | --- | --- | --- | --- | --- | --- | --- | --- | --- | --- | --- | --- | --- | --- | --- | --- |
| Isolate/nt# |  |  |  |  |  |  |  |  |  |  |  |  |  |  |  |  |  |  |  |  |  |  |  |  |  |  |  |  |  |  |  |  |  |  |  |  |  |  |  |  |  |  |  | 1 | 1 | 1 | 1 | 1 | 1 | 1 | 1 | 1 |
|  |  |  | 1 | 1 | 2 | 2 | 2 | 2 | 2 | 3 | 3 | 3 | 3 | 4 | 4 | 4 | 4 | 4 | 4 | 4 | 4 | 4 | 5 | 5 | 5 | 6 | 6 | 7 | 7 | 7 | 7 | 8 | 8 | 8 | 8 | 8 | 8 | 8 | 9 | 9 | 9 | 9 | 9 | 0 | 0 | 0 | 0 | 1 | 1 | 1 | 1 | 1 |
|  | 5 | 6 | 1 | 2 | 0 | 0 | 0 | 1 | 3 | 0 | 4 | 4 | 6 | 0 | 2 | 3 | 3 | 3 | 5 | 7 | 7 | 9 | 1 | 2 | 7 | 4 | 6 | 4 | 5 | 7 | 8 | 0 | 2 | 3 | 4 | 4 | 8 | 9 | 3 | 4 | 5 | 5 | 6 | 4 | 6 | 7 | 8 | 0 | 1 | 4 | 5 | 5 |
|  | 2 | 3 | 7 | 2 | 2 | 4 | 5 | 0 | 1 | 6 | 0 | 8 | 6 | 7 | 8 | 2 | 4 | 5 | 9 | 4 | 7 | 3 | 9 | 4 | 0 | 2 | 0 | 4 | 6 | 5 | 9 | 7 | 8 | 1 | 0 | 9 | 4 | 1 | 0 | 5 | 1 | 4 | 9 | 7 | 5 | 1 | 6 | 7 | 9 | 6 | 6 | 8 |
| **WN-NY99** | **C** | **A** | **G** | **G** | **A** | **C** | **C** | **C** | **A** | **A** | **C** | **T** | **C** | **A** | **T** | **A** | **T** | **C** | **A** | **C** | **T** | **G** | **C** | **C** | **T** | **T** | **C** | **A** | **G** | **C** | **G** | **C** | **G** | **A** | **A** | **C** | **T** | **T** | **T** | **T** | **G** | **C** | **C** | **C** | **C** | **T** | **C** | **T** | **C** | **C** | **A** | **C** |
| BSL53 | . | . | . | . | . | . | . | . | . | . | . | . | . | . | . | . | . | . | . | . | . | . | . | . | . | . | T | . | . | . | . | . | . | . | . | . | C | . | C | . | . | . | . | . | . | C | . | . | . | . | . | . |
| BSL178 | . | . | C | A | . | . | . | . | . | . | . | . | . | . | . | . | . | . | . | . | . | . | . | . | A | . | . | . | . | . | . | T | . | . | T | . | . | . | . | . | . | . | . | . | . | . | . | . | . | . | G | . |
| BSL78 | . | . | . | . | . | T | . | . | . | G | . | . | . | . | . | . | . | . | . | T | . | . | . | . | . | . | . | . | . | . | A | . | . | . | . | . | . | C | . | . | . | . | . | . | . | . | . | . | . | . | . | . |
| BSL85 | . | . | . | . | . | . | . | . | G | . | . | . | . | . | . | . | . | . | . | . | C | A | . | . | . | C | T | . | . | . | . | . | . | . | . | . | . | G | . | C | . | . | . | . | . | . | . | . | . | . | . | . |
| BSL05 | . | G | . | . | . | . | . | . | . | . | . | . | . | . | . | . | . | T | . | . | . | . | . | . | . | C | . | . | A | T | . | . | A | . | . | . | . | . | . | . | A | . | . | . | . | C | . | C | T | T | . | . |
| BSL80 | . | G | . | . | . | . | . | . | . | . | . | . | . | . | . | . | . | T | . | . | . | . | . | . | . | C | . | . | A | T | . | . | A | . | . | . | . | . | . | . | A | . | . | . | . | C | . | C | T | T | . | . |
| BSL93 | . | . | . | . | . | . | . | . | . | . | T | . | T | . | . | . | . | T | . | . | . | . | . | . | . | . | . | . | . | . | . | . | . | . | . | . | . | . | . | . | . | . | . | . | . | . | . | . | . | . | . | . |
| BSL101 | . | . | . | . | . | . | . | . | . | . | T | . | . | . | . | . | . | T | . | . | . | . | . | . | . | . | . | . | . | . | . | . | . | . | . | . | . | . | . | . | . | . | . | . | . | . | . | . | . | . | . | . |
| BSL107 | . | . | . | . | . | . | . | . | . | . | T | . | . | . | . | . | . | T | . | . | . | . | . | . | . | . | . | . | . | . | . | . | . | . | . | . | . | . | . | . | . | . | . | . | . | . | . | . | . | . | . | . |
| BSL116 | . | . | . | . | G | . | . | . | . | . | T | . | . | . | . | . | . | T | . | . | . | . | . | . | . | . | . | . | . | . | . | . | . | . | . | . | . | . | . | . | . | T | . | . | . | . | . | . | . | . | . | . |
| BSL221 | . | . | . | . | . | . | . | . | . | . | T | C | . | . | . | . | . | T | . | . | . | . | T | . | . | . | . | . | . | . | . | . | . | . | . | . | . | . | . | . | . | . | . | . | . | . | . | . | . | . | . | . |
| BSL08 | . | . | . | . | . | . | . | . | . | . | T | . | . | . | . | . | . | T | . | . | . | . | . | T | . | . | . | . | . | . | . | . | . | . | . | . | . | . | . | . | . | . | . | . | . | . | . | . | . | . | . | . |
| BSL140 | . | . | . | . | . | . | . | . | . | . | T | . | . | . | . | . | . | T | . | . | . | . | . | . | . | . | . | . | . | . | . | . | . | . | . | . | . | . | . | . | . | . | . | . | . | . | . | . | . | . | . | . |
| BSL195 | . | . | . | . | . | T | . | . | . | . | T | . | . | G | . | . | . | T | . | . | . | . | . | . | . | . | . | . | . | . | . | . | . | . | . | . | . | . | . | . | . | . | T | . | T | . | . | . | . | . | . | . |
| ARC1 | . | . | . | . | . | . | . | T | . | . | T | . | . | . | . | . | . | . | . | . | . | . | . | . | . | . | . | G | . | . | . | . | . | . | . | . | . | . | . | . | . | . | . | . | . | . | . | . | . | . | . | . |
| ARC3 | T | . | . | . | . | . | T | . | . | . | T | . | . | G | C | . | C | T | C | . | . | . | . | . | . | . | . | . | . | . | . | . | . | . | . | T | . | . | . | . | . | . | T | . | . | . | . | . | . | . | . | . |
| ARC4 | . | . | . | . | . | . | . | . | . | . | T | . | . | . | . | . | . | T | . | . | . | . | . | . | . | . | . | . | . | . | . | . | . | . | . | . | . | . | . | . | . | . | . | T | . | . | T | . | . | . | . | T |
| ARC6 | . | . | . | . | . | . | . | . | . | . | T | . | . | . | . | T | . | T | . | . | . | . | . | . | . | . | . | . | . | . | . | . | . | G | . | . | . | . | . | . | . | . | . | . | . | . | . | . | . | . | . | . |
| ARC13 | . | . | . | . | . | . | . | . | . | . | T | . | . | . | . | . | . | T | . | . | . | . | . | . | . | . | . | . | . | . | . | . | . | . | . | . | . | . | . | . | . | . | . | T | . | . | . | . | . | . | . | . |
|  |  |  |  |  |  |  |  |  |  |  |  |  |  |  |  |  |  |  |  |  |  |  |  |  |  |  |  |  |  |  |  |  |  |  |  |  |  |  |  |  |  |  |  |  |  |  |  |  |  |  |  |  |
| Gene | Env | | | | | | | | | | | | | | | | | | | | | | | | | | | | | | | | | | | | | | | | | | | | | | | | | NS1 | | |
| Isolate/nt# | 1 | 1 | 1 | 1 | 1 | 1 | 1 | 1 | 1 | 1 | 1 | 1 | 1 | 1 | 1 | 1 | 1 | 1 | 1 | 1 | 1 | 1 | 1 | 1 | 1 | 1 | 1 | 1 | 1 | 1 | 1 | 1 | 1 | 1 | 1 | 2 | 2 | 2 | 2 | 2 | 2 | 2 | 2 | 2 | 2 | 2 | 2 | 2 | 2 | 2 | 2 | 2 |
|  | 1 | 1 | 1 | 2 | 2 | 3 | 4 | 4 | 4 | 4 | 4 | 4 | 5 | 5 | 5 | 5 | 5 | 6 | 6 | 6 | 6 | 7 | 7 | 7 | 7 | 8 | 8 | 9 | 9 | 9 | 9 | 9 | 9 | 9 | 9 | 0 | 0 | 0 | 0 | 1 | 2 | 2 | 2 | 3 | 3 | 3 | 3 | 4 | 4 | 4 | 5 | 5 |
|  | 7 | 7 | 8 | 4 | 6 | 2 | 0 | 2 | 4 | 4 | 6 | 6 | 2 | 3 | 5 | 6 | 7 | 0 | 2 | 3 | 6 | 0 | 5 | 5 | 6 | 4 | 7 | 0 | 2 | 5 | 5 | 5 | 6 | 8 | 9 | 1 | 6 | 8 | 9 | 0 | 6 | 9 | 9 | 0 | 5 | 7 | 7 | 4 | 6 | 7 | 6 | 8 |
|  | 0 | 9 | 5 | 2 | 0 | 0 | 1 | 2 | 2 | 6 | 1 | 7 | 1 | 3 | 4 | 7 | 5 | 8 | 0 | 5 | 2 | 7 | 0 | 5 | 2 | 8 | 8 | 8 | 3 | 3 | 6 | 9 | 5 | 3 | 8 | 0 | 7 | 0 | 7 | 9 | 8 | 0 | 2 | 4 | 6 | 0 | 1 | 8 | 6 | 5 | 5 | 6 |
| **WN-NY99** | **C** | **A** | **G** | **C** | **C** | **A** | **A** | **C** | **T** | **A** | **G** | **C** | **G** | **C** | **T** | **T** | **G** | **T** | **C** | **C** | **G** | **C** | **C** | **T** | **T** | **A** | **C** | **T** | **T** | **C** | **T** | **C** | **T** | **T** | **T** | **C** | **C** | **C** | **A** | **C** | **G** | **G** | **C** | **C** | **T** | **C** | **C** | **C** | **C** | **T** | **C** | **A** |
| BSL53 | . | . | A | . | . | G | . | . | C | . | . | . | . | . | . | . | . | . | . | . | . | . | . | . | . | . | . | . | . | . | . | . | . | . | . | . | . | . | . | . | A | . | . | . | . | . | . | . | T | . | . | . |
| BSL178 | . | . | . | . | . | . | G | . | C | . | . | . | . | . | . | . | . | . | . | . | . | . | . | . | . | . | . | . | C | . | . | . | C | . | . | . | . | . | C | . | . | . | . | . | . | . | T | . | T | . | . | . |
| BSL78 | . | . | . | . | . | . | . | T | C | . | A | . | . | . | . | . | . | . | . | . | . | . | . | . | C | . | . | C | . | T | . | T | . | . | . | . | . | . | . | . | . | A | . | . | . | . | . | . | T | . | T | . |
| BSL85 | . | . | . | . | T | . | . | . | C | . | . | . | . | . | . | . | A | . | . | T | . | . | . | . | . | . | . | . | . | . | . | . | . | . | . | . | . | . | . | . | . | . | . | . | . | . | . | . | T | . | . | . |
| BSL05 | . | . | . | . | . | . | . | . | C | . | . | T | . | T | . | . | . | C | . | . | . | . | . | . | . | . | T | . | . | . | A | . | . | C | . | . | . | . | . | . | . | . | . | . | . | . | . | . | T | . | . | G |
| BSL80 | . | . | . | . | . | . | . | . | C | . | . | T | . | T | . | . | . | C | . | . | . | . | . | . | . | . | T | . | . | . | . | . | . | C | C | . | . | . | . | . | . | . | . | . | . | T | . | . | T | . | . | G |
| BSL93 | . | . | . | . | . | . | . | . | C | . | . | . | . | . | . | . | . | . | . | . | A | . | T | . | . | . | . | . | . | . | . | . | . | . | . | T | . | . | . | T | . | . | . | T | . | . | . | . | T | C | . | . |
| BSL101 | . | . | . | . | . | . | . | . | C | . | . | . | . | . | . | . | . | . | T | . | A | . | T | . | . | . | . | . | . | . | . | . | . | . | . | . | . | T | . | T | . | . | . | T | . | . | . | . | T | C | . | . |
| BSL107 | . | . | . | . | . | . | . | . | C | G | . | . | . | . | . | . | . | . | . | . | A | . | T | . | . | . | T | . | . | . | . | . | . | . | . | . | . | . | . | T | . | . | . | T | . | . | . | . | T | C | . | . |
| BSL116 | . | G | . | . | . | . | . | . | C | . | . | . | . | . | . | . | . | . | . | . | A | . | T | . | . | . | . | . | C | . | . | . | . | . | . | . | . | . | . | T | . | . | T | T | . | . | . | . | T | . | . | . |
| BSL221 | . | . | . | . | . | . | . | . | C | . | . | . | . | . | . | . | . | . | . | . | A | . | T | . | . | . | . | . | . | . | . | . | . | . | . | . | . | . | . | T | . | . | . | T | . | . | . | . | T | C | . | . |
| BSL08 | . | . | . | . | . | . | . | . | C | . | . | . | . | . | . | . | . | . | . | . | A | . | T | . | . | . | . | . | . | . | . | . | . | . | . | . | . | . | . | T | . | . | . | T | . | . | . | T | T | . | . | . |
| BSL140 | . | . | . | . | . | . | . | . | C | . | . | . | . | . | C | . | . | . | . | . | . | . | T | . | . | G | . | . | . | . | . | . | . | . | . | . | . | . | . | T | . | . | . | T | . | . | . | . | T | C | . | . |
| BSL195 | T | . | . | . | . | . | . | . | C | . | . | . | . | . | . | C | . | . | . | . | . | . | T | . | . | . | . | . | . | . | . | . | . | . | . | . | . | . | . | . | . | . | . | T | . | . | . | . | T | . | . | . |
| ARC1 | . | . | . | . | . | . | . | . | C | . | . | . | . | . | . | . | . | . | . | . | A | . | T | . | . | G | . | . | . | . | . | . | . | . | . | . | T | . | . | T | . | . | . | T | C | . | . | . | T | . | . | . |
| ARC3 | T | . | . | . | . | . | . | . | C | . | . | . | . | . | . | . | . | . | . | . | A | T | T | . | . | . | . | . | . | . | . | . | . | . | . | . | . | . | . | . | . | . | . | T | . | . | . | . | T | . | . | . |
| ARC4 | . | . | . | T | . | G | . | . | C | . | . | . | . | . | . | . | . | . | . | . | A | . | T | . | . | G | . | . | . | . | . | . | . | . | . | . | . | . | . | . | . | . | . | T | . | . | . | . | T | . | . | . |
| ARC6 | . | . | . | . | . | . | . | . | C | . | . | . | . | . | . | . | . | . | . | . | A | . | T | . | . | . | . | . | . | . | . | . | . | . | . | . | . | . | . | T | . | . | . | T | . | . | . | . | T | C | . | . |
| ARC13 | . | . | . | . | . | . | . | . | C | . | . | . | A | . | . | . | . | . | . | . | A | . | T | C | . | . | . | . | . | . | . | . | . | . | . | . | . | . | . | T | . | . | . | T | . | . | . | . | T | . | . | . |
|  |  |  |  |  |  |  |  |  |  |  |  |  |  |  |  |  |  |  |  |  |  |  |  |  |  |  |  |  |  |  |  |  |  |  |  |  |  |  |  |  |  |  |  |  |  |  |  |  |  |  |  |  |
| Gene | NS1 | | | | | | | | | | | | | | | | | | | | | | | | | | | | | | | | | | | | | | NS2A | | | | | | | | | | | | | |
| Isolate/nt# | 2 | 2 | 2 | 2 | 2 | 2 | 2 | 2 | 2 | 2 | 2 | 2 | 2 | 2 | 2 | 2 | 3 | 3 | 3 | 3 | 3 | 3 | 3 | 3 | 3 | 3 | 3 | 3 | 3 | 3 | 3 | 3 | 3 | 3 | 3 | 3 | 3 | 3 | 3 | 3 | 3 | 3 | 3 | 3 | 3 | 3 | 3 | 3 | 3 | 3 | 3 | 3 |
|  | 6 | 7 | 7 | 7 | 7 | 7 | 7 | 7 | 8 | 8 | 8 | 8 | 8 | 9 | 9 | 9 | 0 | 0 | 0 | 0 | 1 | 1 | 1 | 1 | 2 | 2 | 2 | 3 | 3 | 3 | 3 | 3 | 3 | 4 | 4 | 4 | 5 | 5 | 5 | 5 | 5 | 5 | 5 | 6 | 6 | 6 | 6 | 6 | 7 | 7 | 7 | 8 |
|  | 4 | 0 | 1 | 2 | 5 | 5 | 6 | 7 | 0 | 3 | 4 | 6 | 9 | 0 | 3 | 9 | 4 | 7 | 8 | 9 | 2 | 3 | 7 | 7 | 4 | 5 | 8 | 0 | 4 | 4 | 5 | 6 | 9 | 2 | 3 | 4 | 0 | 0 | 2 | 3 | 4 | 7 | 9 | 4 | 4 | 7 | 9 | 9 | 2 | 4 | 8 | 0 |
|  | 9 | 3 | 8 | 4 | 1 | 4 | 0 | 2 | 2 | 2 | 4 | 2 | 8 | 4 | 4 | 7 | 5 | 8 | 3 | 3 | 6 | 8 | 1 | 5 | 6 | 2 | 8 | 0 | 5 | 7 | 1 | 2 | 9 | 3 | 5 | 7 | 1 | 4 | 8 | 1 | 6 | 0 | 2 | 6 | 9 | 2 | 3 | 7 | 0 | 7 | 3 | 8 |
| **WN-NY99** | **T** | **T** | **T** | **C** | **G** | **A** | **C** | **T** | **G** | **T** | **A** | **C** | **T** | **T** | **G** | **A** | **G** | **A** | **G** | **T** | **A** | **T** | **T** | **A** | **G** | **G** | **A** | **C** | **A** | **A** | **T** | **C** | **T** | **A** | **C** | **C** | **C** | **C** | **T** | **T** | **C** | **C** | **G** | **C** | **C** | **T** | **C** | **G** | **C** | **G** | **A** | **C** |
| BSL53 | . | . | . | . | . | . | T | . | . | . | . | . | . | . | A | . | . | . | . | . | G | . | A | . | C | . | . | . | . | G | . | . | C | . | . | . | . | . | . | . | . | . | . | . | . | . | . | . | . | A | G | . |
| BSL178 | . | . | . | . | . | . | . | . | . | . | . | . | . | . | . | . | . | . | . | . | . | C | . | . | . | . | . | . | . | . | . | . | . | . | . | . | . | . | . | . | . | . | . | . | . | . | . | . | . | . | . | T |
| BSL78 | . | . | C | . | T | T | . | . | . | . | . | . | . | . | . | . | . | . | . | . | . | . | . | . | . | . | . | . | . | . | . | . | . | . | . | . | . | T | . | . | T | . | . | . | . | . | . | . | . | . | . | . |
| BSL85 | . | . | . | . | . | . | . | . | . | . | . | . | . | . | . | . | . | . | . | . | . | . | . | . | . | . | . | T | . | . | . | . | . | . | . | . | . | . | . | . | . | T | . | . | . | . | . | . | . | . | . | . |
| BSL05 | . | C | . | . | . | . | . | . | A | . | . | . | . | C | . | G | . | . | . | . | . | . | . | . | . | . | . | . | . | . | C | . | . | G | . | T | . | . | . | . | . | . | . | . | . | . | T | . | . | . | . | . |
| BSL80 | C | C | . | . | . | . | . | . | . | . | . | . | . | C | . | G | . | . | . | . | . | . | . | . | . | . | . | . | . | . | C | . | . | G | . | T | . | . | . | . | . | . | . | . | . | . | T | . | . | . | . | . |
| BSL93 | . | . | . | . | . | . | . | . | . | . | . | . | . | . | . | . | . | G | . | . | . | . | . | . | . | . | . | . | . | . | . | . | . | . | . | . | T | . | C | . | . | . | . | . | T | . | . | A | . | . | . | . |
| BSL101 | . | . | . | . | . | . | . | . | . | . | . | . | . | . | . | . | . | . | . | . | . | . | . | . | . | . | . | . | . | . | . | . | . | . | . | . | T | . | C | . | . | . | . | . | . | . | . | A | . | . | . | . |
| BSL107 | . | . | . | . | . | . | . | . | . | . | . | . | . | . | . | . | . | . | . | . | . | . | . | . | . | . | . | . | . | . | . | . | . | . | . | . | T | . | C | . | . | . | A | . | . | . | . | A | . | . | . | . |
| BSL116 | . | . | . | . | . | . | . | . | . | . | . | T | . | . | . | . | . | . | . | . | . | . | . | . | . | . | . | . | . | . | . | T | . | . | . | . | T | . | C | C | . | . | . | T | . | . | . | . | T | . | . | . |
| BSL221 | . | . | . | . | . | . | . | C | . | . | . | . | . | . | . | . | . | . | T | . | . | . | . | . | . | . | . | . | . | . | . | . | . | . | T | . | T | . | C | . | . | . | . | . | . | . | . | A | . | . | . | . |
| BSL08 | . | . | . | . | . | . | . | . | . | . | . | . | . | . | . | . | . | . | . | . | . | . | . | . | . | . | . | . | . | . | . | . | . | . | . | . | T | . | C | . | . | . | . | . | . | . | . | . | . | . | . | . |
| BSL140 | . | . | . | . | . | . | . | . | . | . | . | . | . | . | . | . | A | . | . | . | . | . | . | . | . | . | . | . | . | . | . | . | . | . | . | . | T | . | C | . | . | . | . | . | . | . | . | A | . | . | . | . |
| BSL195 | . | . | . | . | . | . | . | . | . | . | . | . | C | . | . | . | . | . | . | . | . | . | . | G | . | A | . | T | . | . | . | . | . | . | . | . | . | . | C | . | . | . | . | . | . | C | . | . | . | . | . | . |
| ARC1 | . | . | . | . | . | . | . | . | . | . | . | . | . | . | . | . | . | G | . | . | . | . | . | . | . | . | . | . | . | . | . | . | . | . | . | . | T | . | C | . | . | . | . | . | . | . | . | . | . | . | . | . |
| ARC3 | . | . | . | T | . | . | . | . | . | . | . | . | C | . | . | . | . | . | . | C | . | . | . | G | . | . | . | . | G | . | . | . | . | . | . | . | . | . | C | . | . | . | . | . | . | . | . | . | . | . | . | . |
| ARC4 | . | . | . | . | . | . | . | . | . | . | . | . | . | . | . | . | . | . | . | . | . | . | . | . | . | . | T | . | . | . | . | . | . | . | . | . | . | . | C | . | . | . | . | . | . | . | . | . | . | . | . | . |
| ARC6 | . | . | . | . | . | . | . | . | . | . | G | . | . | . | . | . | . | . | . | . | . | . | . | . | . | . | . | . | . | . | . | . | . | . | . | . | T | . | C | . | . | . | . | . | . | . | . | A | . | . | . | . |
| ARC13 | . | . | . | . | . | . | . | . | . | A | . | . | . | . | . | . | . | . | . | . | . | . | . | . | . | . | . | . | . | . | . | . | . | . | . | . | T | . | C | . | . | . | . | . | . | . | . | . | . | . | . | . |
|  |  |  |  |  |  |  |  |  |  |  |  |  |  |  |  |  |  |  |  |  |  |  |  |  |  |  |  |  |  |  |  |  |  |  |  |  |  |  |  |  |  |  |  |  |  |  |  |  |  |  |  |  |
| Gene | NS2A | | | | | | | | | | | | | | | | | | | NS2B | | | | | | | | | | | | | | | | | | NS3 | | | | | | | | | | | | | | |
| Isolate/nt# | 3 | 3 | 3 | 3 | 3 | 3 | 3 | 3 | 3 | 4 | 4 | 4 | 4 | 4 | 4 | 4 | 4 | 4 | 4 | 4 | 4 | 4 | 4 | 4 | 4 | 4 | 4 | 4 | 4 | 4 | 4 | 4 | 4 | 4 | 4 | 4 | 4 | 4 | 4 | 4 | 4 | 4 | 4 | 4 | 4 | 4 | 4 | 4 | 4 | 4 | 4 | 4 |
|  | 8 | 8 | 8 | 8 | 9 | 9 | 9 | 9 | 9 | 0 | 0 | 0 | 0 | 1 | 1 | 1 | 1 | 1 | 1 | 2 | 2 | 2 | 2 | 2 | 3 | 3 | 3 | 3 | 3 | 4 | 5 | 5 | 5 | 5 | 5 | 5 | 6 | 6 | 7 | 7 | 7 | 8 | 8 | 8 | 8 | 8 | 8 | 9 | 9 | 9 | 9 | 9 |
|  | 5 | 5 | 6 | 8 | 1 | 3 | 4 | 5 | 6 | 5 | 8 | 8 | 9 | 2 | 2 | 4 | 7 | 8 | 9 | 2 | 4 | 7 | 8 | 9 | 0 | 2 | 5 | 6 | 8 | 1 | 3 | 4 | 4 | 6 | 6 | 7 | 1 | 2 | 2 | 4 | 6 | 0 | 0 | 2 | 3 | 4 | 5 | 0 | 2 | 3 | 4 | 5 |
|  | 0 | 8 | 1 | 0 | 2 | 9 | 0 | 2 | 9 | 7 | 0 | 8 | 4 | 3 | 9 | 6 | 3 | 9 | 4 | 7 | 5 | 2 | 7 | 0 | 2 | 0 | 3 | 8 | 0 | 6 | 0 | 2 | 8 | 3 | 4 | 6 | 1 | 9 | 5 | 9 | 4 | 3 | 9 | 7 | 0 | 8 | 4 | 5 | 9 | 5 | 4 | 9 |
| **WN-NY99** | **T** | **T** | **T** | **C** | **C** | **A** | **G** | **C** | **C** | **C** | **C** | **G** | **A** | **G** | **C** | **A** | **G** | **C** | **T** | **C** | **A** | **C** | **G** | **T** | **C** | **T** | **T** | **A** | **T** | **A** | **T** | **T** | **C** | **C** | **T** | **G** | **A** | **C** | **G** | **C** | **T** | **C** | **C** | **C** | **G** | **C** | **A** | **T** | **T** | **C** | **G** | **C** |
| BSL53 | C | . | . | . | . | . | . | . | . | . | . | . | G | . | . | G | . | . | . | T | . | . | . | . | . | . | . | . | . | . | . | . | . | . | . | . | . | . | . | . | . | T | . | . | . | . | . | . | . | . | . | . |
| BSL178 | . | . | C | . | . | . | . | . | . | . | . | . | . | . | . | G | . | . | C | . | . | . | . | . | . | . | . | . | . | . | . | . | . | . | C | . | G | . | A | . | . | T | . | . | A | T | . | . | . | . | A | . |
| BSL78 | . | . | . | T | . | . | . | . | . | . | . | . | . | . | T | G | . | . | . | . | G | . | . | . | . | C | . | G | . | . | . | . | . | T | . | . | . | . | . | . | C | T | . | . | . | . | T | . | . | T | . | T |
| BSL85 | . | . | . | . | . | . | . | . | . | . | . | . | . | T | T | G | . | . | . | . | . | T | . | A | . | . | C | . | . | . | . | C | . | . | . | . | . | . | . | . | . | T | . | T | . | . | . | . | . | . | . | . |
| BSL05 | . | C | . | . | . | . | . | T | . | . | . | . | . | . | . | G | . | T | . | . | . | . | A | . | T | . | . | . | . | . | . | . | T | T | . | . | . | . | . | . | . | T | . | . | . | . | . | . | C | . | A | . |
| BSL80 | . | C | . | . | . | . | . | T | . | . | . | . | . | . | . | G | . | T | . | . | . | . | A | . | T | . | . | . | . | . | . | . | T | T | . | . | . | . | . | . | . | T | . | . | . | . | . | . | C | . | . | . |
| BSL93 | . | . | . | . | T | . | . | . | T | . | . | A | . | . | . | G | . | . | . | . | . | . | . | . | . | . | . | . | . | . | C | . | . | . | . | . | . | . | . | T | . | T | . | . | . | . | . | . | . | . | . | . |
| BSL101 | . | . | . | . | T | . | . | . | T | . | . | A | . | . | . | G | . | . | . | . | . | . | . | . | . | . | . | . | . | . | C | . | . | . | . | . | . | . | . | T | . | T | . | . | . | . | . | . | . | . | . | . |
| BSL107 | . | . | . | . | T | . | . | . | T | . | . | A | . | . | . | G | . | . | . | . | . | . | . | . | . | . | . | . | . | . | C | . | . | . | . | . | . | . | . | T | . | T | . | . | . | . | . | . | . | . | . | . |
| BSL116 | . | . | . | . | T | . | . | . | T | . | . | A | . | . | . | G | . | . | . | . | . | . | . | . | . | . | . | . | . | . | C | . | . | . | . | . | . | . | . | T | . | T | . | . | . | . | . | . | . | . | . | . |
| BSL221 | . | . | . | . | T | . | . | . | T | . | . | A | . | . | . | G | . | . | . | . | . | . | . | . | . | . | . | . | . | . | C | . | . | . | . | . | . | . | . | T | . | T | . | . | . | . | . | . | . | . | . | . |
| BSL08 | . | . | . | . | T | G | . | . | T | . | . | A | . | . | . | G | . | . | . | . | . | . | . | . | . | . | . | . | . | . | C | . | . | . | . | . | . | . | . | T | . | T | . | . | . | . | . | . | . | . | . | . |
| BSL140 | . | . | . | . | T | . | . | . | T | . | . | A | . | . | . | G | . | . | . | . | . | . | . | . | . | . | . | . | . | . | C | . | . | . | . | . | . | . | . | T | . | T | . | . | . | . | . | . | . | . | . | . |
| BSL195 | . | . | . | . | . | . | . | . | T | T | T | A | . | . | . | G | . | . | . | . | . | . | . | C | . | . | C | . | . | . | C | . | . | . | . | A | . | T | . | . | . | T | . | . | . | . | . | . | . | . | . | . |
| ARC1 | . | . | . | . | T | . | . | . | T | . | . | A | . | . | . | G | . | . | . | . | . | . | . | . | . | . | . | . | . | G | C | . | . | . | . | . | . | . | . | T | . | T | . | . | . | . | . | . | . | . | . | . |
| ARC3 | . | . | . | . | . | . | . | . | T | . | . | A | . | . | . | G | T | . | . | . | . | . | . | C | . | . | . | . | . | . | C | . | . | . | . | A | . | . | . | . | . | T | . | . | . | . | . | . | . | . | . | . |
| ARC4 | . | . | . | . | . | . | . | . | T | . | . | A | . | . | . | G | . | . | . | . | . | . | . | . | . | . | . | . | . | . | C | . | . | . | . | . | . | . | . | T | . | T | T | . | . | . | . | . | . | . | . | . |
| ARC6 | . | . | . | . | T | . | . | . | T | . | . | A | . | . | . | G | . | . | . | . | . | . | . | . | . | . | . | . | C | . | C | . | . | . | . | . | . | . | . | T | . | T | . | . | . | . | . | . | . | . | . | . |
| ARC13 | . | . | . | . | T | . | A | . | T | . | . | . | . | . | . | G | . | . | . | . | . | . | . | . | . | . | . | . | . | . | C | . | . | . | . | . | . | . | . | T | . | T | . | . | . | . | . | C | . | . | . | . |
|  |  |  |  |  |  |  |  |  |  |  |  |  |  |  |  |  |  |  |  |  |  |  |  |  |  |  |  |  |  |  |  |  |  |  |  |  |  |  |  |  |  |  |  |  |  |  |  |  |  |  |  |  |
| Gene | NS3 | | | | | | | | | | | | | | | | | | | | | | | | | | | | | | | | | | | | | | | | | | | | | | | | | | | |
| Isolate/nt# | 4 | 4 | 4 | 4 | 5 | 5 | 5 | 5 | 5 | 5 | 5 | 5 | 5 | 5 | 5 | 5 | 5 | 5 | 5 | 5 | 5 | 5 | 5 | 5 | 5 | 5 | 5 | 5 | 5 | 5 | 5 | 5 | 5 | 5 | 5 | 5 | 5 | 5 | 5 | 5 | 5 | 5 | 5 | 5 | 5 | 6 | 6 | 6 | 6 | 6 | 6 | 6 |
|  | 9 | 9 | 9 | 9 | 0 | 0 | 1 | 1 | 1 | 1 | 2 | 2 | 3 | 3 | 3 | 4 | 4 | 4 | 4 | 4 | 4 | 4 | 5 | 5 | 5 | 5 | 5 | 5 | 5 | 6 | 6 | 6 | 6 | 6 | 7 | 7 | 7 | 8 | 8 | 8 | 8 | 8 | 9 | 9 | 9 | 0 | 0 | 0 | 0 | 0 | 1 | 1 |
|  | 6 | 7 | 8 | 8 | 8 | 9 | 0 | 4 | 6 | 7 | 8 | 9 | 1 | 5 | 6 | 0 | 2 | 3 | 5 | 5 | 6 | 8 | 1 | 2 | 2 | 4 | 5 | 5 | 6 | 1 | 5 | 6 | 7 | 9 | 4 | 6 | 8 | 0 | 1 | 6 | 7 | 8 | 1 | 1 | 9 | 0 | 0 | 3 | 4 | 6 | 0 | 3 |
|  | 2 | 1 | 6 | 9 | 8 | 7 | 0 | 8 | 1 | 8 | 6 | 2 | 0 | 6 | 7 | 0 | 1 | 0 | 1 | 7 | 9 | 7 | 1 | 6 | 9 | 7 | 0 | 3 | 5 | 1 | 8 | 7 | 4 | 7 | 5 | 6 | 1 | 0 | 7 | 5 | 1 | 9 | 0 | 9 | 4 | 6 | 9 | 9 | 8 | 3 | 2 | 8 |
| **WN-NY99** | **A** | **A** | **C** | **G** | **C** | **A** | **C** | **C** | **C** | **C** | **C** | **T** | **T** | **C** | **T** | **T** | **G** | **T** | **C** | **C** | **G** | **A** | **C** | **C** | **G** | **A** | **A** | **C** | **C** | **T** | **C** | **C** | **T** | **T** | **T** | **A** | **G** | **A** | **T** | **C** | **G** | **C** | **C** | **A** | **T** | **T** | **G** | **G** | **G** | **G** | **C** | **C** |
| BSL53 | G | . | . | . | . | . | . | . | . | . | . | . | . | . | . | . | . | . | . | . | . | G | . | . | . | . | . | . | . | . | . | . | . | . | . | . | . | . | . | . | . | . | . | . | . | . | . | . | . | . | . | . |
| BSL178 | . | G | T | . | . | . | . | T | . | . | . | . | . | . | . | . | . | C | . | . | . | . | . | . | . | . | . | . | . | A | T | T | . | . | . | . | . | . | . | . | . | . | . | . | C | . | . | . | A | . | T | . |
| BSL78 | . | . | . | . | . | . | . | . | . | T | . | A | . | . | . | . | . | . | . | . | . | . | . | . | A | . | C | . | . | . | . | . | . | . | . | G | A | . | C | T | . | . | . | . | . | C | . | . | . | . | . | T |
| BSL85 | . | . | . | A | . | G | T | . | . | . | T | . | . | . | . | . | A | . | . | . | A | . | . | . | . | . | . | . | . | . | . | . | . | . | . | . | . | . | . | . | . | . | T | . | . | . | . | . | . | . | . | T |
| BSL05 | . | . | T | . | . | . | . | . | . | . | . | . | . | . | . | . | . | . | T | . | . | . | . | . | . | . | . | . | . | . | . | . | . | . | . | . | . | . | . | . | . | . | . | . | . | . | . | A | . | . | . | T |
| BSL80 | . | . | T | . | . | . | . | . | . | . | . | . | . | . | . | . | . | . | T | . | . | . | . | . | . | . | . | . | . | . | . | . | . | . | . | . | . | . | . | . | . | . | . | . | . | . | . | A | . | . | . | T |
| BSL93 | . | . | . | . | . | . | . | . | . | . | . | . | . | . | . | C | . | . | . | T | . | . | . | T | . | . | . | . | . | . | . | . | . | . | . | . | . | . | . | . | . | T | . | . | . | . | . | . | . | T | . | T |
| BSL101 | . | . | . | . | . | . | . | . | . | . | . | . | . | . | . | C | . | . | . | T | . | . | . | . | . | . | . | T | . | . | . | . | . | C | . | . | . | . | . | . | . | T | . | . | . | . | . | . | . | T | . | T |
| BSL107 | . | . | . | . | . | . | . | . | . | . | . | . | C | . | . | C | . | . | . | T | . | . | . | . | . | . | . | . | . | . | . | . | . | . | . | . | . | . | . | . | . | T | . | . | . | . | . | . | . | T | . | T |
| BSL116 | . | . | . | . | . | . | . | . | . | . | . | . | . | . | . | C | . | . | . | T | . | . | . | . | . | . | . | . | . | . | . | . | . | . | . | . | . | . | . | . | . | T | . | . | . | . | . | . | . | T | . | T |
| BSL221 | . | . | . | . | . | . | . | . | . | . | . | . | . | . | . | C | . | . | . | T | . | . | T | . | . | G | . | . | . | . | . | . | . | . | . | . | . | . | . | . | . | T | . | . | . | . | A | . | . | T | . | T |
| BSL08 | . | . | . | . | . | . | . | . | . | . | . | . | . | . | C | C | . | . | . | T | . | . | . | . | . | . | . | . | . | . | . | . | . | . | . | . | . | G | . | . | . | T | . | G | . | . | . | . | . | T | . | T |
| BSL140 | . | . | . | . | . | . | . | . | T | . | . | . | . | . | C | C | . | . | . | T | . | . | . | . | . | . | . | . | T | . | . | . | . | . | . | . | . | . | . | . | . | . | . | . | . | . | . | . | . | . | . | T |
| BSL195 | . | . | . | . | T | . | . | . | . | . | . | . | . | T | . | . | . | . | . | T | . | . | . | . | . | . | . | . | . | . | . | . | . | . | . | . | . | . | . | . | . | T | . | . | . | . | . | . | . | T | . | T |
| ARC1 | . | . | . | . | . | . | . | . | . | . | . | . | . | . | . | C | . | . | . | T | . | . | . | . | . | . | . | . | . | . | . | . | . | . | . | . | . | . | . | . | . | T | . | . | . | . | . | . | . | T | . | T |
| ARC3 | . | . | . | . | . | . | . | . | . | . | . | . | . | . | . | . | . | . | . | T | . | . | . | . | . | . | . | . | . | . | . | . | . | . | . | . | . | . | . | . | A | T | . | . | . | . | . | . | . | T | . | T |
| ARC4 | . | . | . | . | . | . | . | . | . | . | . | . | . | . | . | . | . | . | . | . | . | . | . | . | . | . | . | . | . | . | . | . | C | . | . | . | . | . | . | . | . | T | . | . | . | . | . | . | . | T | . | T |
| ARC6 | . | . | . | . | . | . | . | . | . | . | . | . | . | . | . | C | . | . | . | T | . | . | . | . | . | . | . | . | . | . | . | . | . | . | C | . | . | . | . | . | . | T | . | . | . | . | . | . | . | T | . | T |
| ARC13 | . | . | . | . | . | . | . | . | . | . | . | . | . | . | . | . | . | . | . | . | . | . | . | . | . | . | . | . | . | . | . | . | . | . | . | . | . | . | . | T | . | T | . | . | . | . | . | . | . | T | . | T |
|  |  |  |  |  |  |  |  |  |  |  |  |  |  |  |  |  |  |  |  |  |  |  |  |  |  |  |  |  |  |  |  |  |  |  |  |  |  |  |  |  |  |  |  |  |  |  |  |  |  |  |  |  |
| Gene | NS3 | | | | | | | | | | | | | | | | | NS4A | | | | | | | | | | | | | | | | | | | | | | | | | | | | NS4B | | | | | | |
| Isolate/nt# | 6 | 6 | 6 | 6 | 6 | 6 | 6 | 6 | 6 | 6 | 6 | 6 | 6 | 6 | 6 | 6 | 6 | 6 | 6 | 6 | 6 | 6 | 6 | 6 | 6 | 6 | 6 | 6 | 6 | 6 | 6 | 6 | 6 | 6 | 6 | 6 | 6 | 6 | 6 | 6 | 6 | 6 | 6 | 6 | 6 | 6 | 6 | 6 | 6 | 6 | 6 | 6 |
|  | 1 | 1 | 1 | 2 | 2 | 2 | 2 | 3 | 3 | 3 | 3 | 4 | 4 | 4 | 4 | 4 | 4 | 4 | 5 | 5 | 5 | 5 | 5 | 5 | 6 | 6 | 6 | 6 | 6 | 7 | 7 | 7 | 7 | 7 | 7 | 7 | 7 | 7 | 7 | 8 | 8 | 8 | 8 | 8 | 9 | 9 | 9 | 9 | 9 | 9 | 9 | 9 |
|  | 6 | 6 | 7 | 0 | 2 | 5 | 9 | 1 | 3 | 3 | 5 | 2 | 2 | 2 | 5 | 6 | 6 | 9 | 2 | 3 | 4 | 5 | 8 | 9 | 1 | 3 | 4 | 7 | 7 | 0 | 1 | 2 | 2 | 2 | 3 | 4 | 5 | 5 | 6 | 1 | 3 | 6 | 7 | 7 | 0 | 1 | 2 | 3 | 5 | 5 | 5 | 7 |
|  | 2 | 5 | 4 | 4 | 9 | 8 | 7 | 2 | 1 | 3 | 5 | 0 | 3 | 6 | 6 | 2 | 8 | 2 | 2 | 4 | 0 | 8 | 1 | 8 | 8 | 9 | 3 | 2 | 5 | 5 | 2 | 1 | 6 | 7 | 4 | 1 | 0 | 9 | 5 | 6 | 4 | 5 | 1 | 7 | 1 | 8 | 7 | 6 | 6 | 8 | 9 | 7 |
| **WN-NY99** | **A** | **T** | **T** | **A** | **A** | **C** | **G** | **T** | **T** | **A** | **G** | **G** | **T** | **C** | **C** | **A** | **T** | **T** | **G** | **A** | **T** | **G** | **G** | **C** | **T** | **T** | **T** | **C** | **C** | **G** | **T** | **G** | **C** | **T** | **T** | **C** | **T** | **T** | **T** | **T** | **A** | **C** | **G** | **C** | **A** | **C** | **T** | **T** | **G** | **A** | **G** | **T** |
| BSL53 | . | . | . | . | . | . | . | . | . | . | . | . | . | T | . | . | . | . | . | . | . | . | A | . | . | . | . | . | . | . | . | A | . | . | . | . | . | . | C | . | . | T | . | . | . | T | . | C | . | . | . | . |
| BSL178 | . | . | . | . | . | . | . | . | . | . | . | . | . | T | . | . | C | . | A | . | . | . | . | T | . | . | . | . | . | A | C | . | . | . | . | . | C | . | . | . | . | . | . | . | . | . | . | . | T | . | A | C |
| BSL78 | G | . | C | . | . | . | . | . | . | . | . | . | . | T | . | . | . | . | . | . | . | A | . | . | . | . | . | T | . | . | . | . | . | . | . | . | . | . | . | C | . | . | . | T | . | . | . | . | . | . | . | . |
| BSL85 | . | . | . | G | . | . | . | . | . | . | . | A | . | T | . | . | . | . | . | . | . | . | . | . | . | . | . | T | . | . | . | . | . | C | . | . | . | C | . | . | . | . | . | . | G | . | . | . | . | C | . | . |
| BSL05 | . | . | . | . | . | . | . | . | . | . | . | . | . | T | T | . | . | C | . | . | . | . | . | . | . | . | . | . | . | . | . | . | T | . | . | . | . | . | . | . | T | . | . | . | . | . | . | . | . | . | A | . |
| BSL80 | . | . | . | . | . | T | A | . | . | . | . | . | . | T | T | . | . | C | . | . | . | . | . | . | . | . | . | . | . | . | . | . | T | . | . | . | . | . | . | . | T | . | . | . | . | . | . | . | . | . | A | . |
| BSL93 | . | C | . | . | . | . | . | C | . | . | A | . | . | T | . | . | . | . | . | . | C | . | . | . | C | C | . | . | T | . | . | . | . | . | . | . | . | . | . | . | . | . | . | . | . | . | . | . | . | . | . | . |
| BSL101 | . | C | . | . | . | . | . | C | . | . | . | . | . | T | . | . | . | . | . | . | C | . | . | . | C | C | C | . | T | . | . | . | . | . | . | . | . | . | . | . | . | . | A | . | . | . | . | . | . | . | . | . |
| BSL107 | . | C | . | . | . | . | . | C | . | . | . | . | . | T | . | . | . | . | . | . | C | . | . | . | C | C | . | . | T | . | . | . | . | . | . | . | . | . | . | . | . | . | . | . | . | . | . | . | . | . | . | . |
| BSL116 | . | C | . | G | . | . | . | C | C | . | . | . | . | . | . | . | . | . | . | . | . | . | . | . | . | . | . | . | . | . | . | . | . | . | . | . | . | . | . | . | . | . | . | . | . | . | . | . | . | . | . | . |
| BSL221 | . | C | . | . | . | . | . | C | . | . | . | . | . | T | . | . | . | . | . | . | C | . | . | . | C | C | . | . | T | . | . | . | . | . | . | . | . | . | . | . | . | . | . | . | . | . | . | . | . | . | . | . |
| BSL08 | . | C | . | . | . | . | . | C | . | . | . | . | . | T | . | . | . | . | . | . | C | . | . | . | C | C | . | . | . | . | . | . | . | . | . | . | . | . | . | . | . | . | . | . | . | . | . | . | . | . | . | . |
| BSL140 | . | C | . | . | G | . | . | C | . | . | . | . | . | T | . | . | . | . | . | . | C | . | . | . | C | C | . | . | T | . | . | . | . | . | . | . | . | . | . | . | . | . | . | . | . | . | . | . | . | . | . | . |
| BSL195 | . | C | . | . | . | . | . | C | . | . | . | . | C | T | . | G | . | . | . | G | C | . | . | . | . | . | . | . | . | . | . | . | . | . | . | . | . | . | . | . | . | . | . | . | . | . | C | . | . | . | . | . |
| ARC1 | G | C | . | . | . | . | . | C | . | . | . | . | . | T | . | . | . | . | . | . | C | . | . | . | C | . | . | . | . | . | . | . | . | . | . | . | . | . | . | . | . | . | . | . | . | . | . | . | . | . | . | . |
| ARC3 | . | C | . | . | . | . | . | C | . | . | . | . | . | T | . | G | . | . | . | G | C | . | . | . | . | . | . | T | . | . | . | . | . | . | C | . | . | . | . | . | . | . | . | . | . | . | C | . | . | . | . | . |
| ARC4 | . | C | . | . | . | . | . | C | . | . | . | . | . | T | . | . | . | . | . | . | C | . | . | . | C | . | . | . | . | . | . | . | . | . | . | T | . | . | . | . | . | . | . | . | . | . | . | . | . | . | . | . |
| ARC6 | . | C | . | . | . | . | . | C | . | . | . | . | . | T | . | . | . | . | . | . | C | . | . | . | C | C | . | . | T | . | . | . | . | . | . | . | . | . | . | . | . | . | . | . | . | . | . | . | . | . | . | . |
| ARC13 | . | C | . | . | . | . | . | C | . | G | . | . | . | T | . | . | A | . | . | . | C | . | . | . | C | . | . | . | . | . | . | . | . | . | . | . | . | . | . | . | . | . | . | . | . | . | . | . | . | . | . | . |
|  |  |  |  |  |  |  |  |  |  |  |  |  |  |  |  |  |  |  |  |  |  |  |  |  |  |  |  |  |  |  |  |  |  |  |  |  |  |  |  |  |  |  |  |  |  |  |  |  |  |  |  |  |
| Gene | NS4B | | | | | | | | | | | | | | | | | | | | | | | | | | | | | | | | | | | | | | | | NS5 | | | | | | | | | | | |
| Isolate/nt# | 6 | 7 | 7 | 7 | 7 | 7 | 7 | 7 | 7 | 7 | 7 | 7 | 7 | 7 | 7 | 7 | 7 | 7 | 7 | 7 | 7 | 7 | 7 | 7 | 7 | 7 | 7 | 7 | 7 | 7 | 7 | 7 | 7 | 7 | 7 | 7 | 7 | 7 | 7 | 7 | 7 | 7 | 7 | 7 | 7 | 7 | 7 | 7 | 7 | 7 | 7 | 7 |
|  | 9 | 0 | 0 | 0 | 1 | 1 | 1 | 1 | 1 | 2 | 2 | 2 | 2 | 2 | 2 | 2 | 3 | 3 | 3 | 3 | 3 | 3 | 3 | 4 | 4 | 4 | 4 | 5 | 5 | 5 | 5 | 5 | 5 | 6 | 6 | 6 | 6 | 7 | 7 | 7 | 7 | 8 | 8 | 8 | 8 | 8 | 8 | 8 | 9 | 9 | 9 | 9 |
|  | 9 | 1 | 2 | 2 | 3 | 5 | 8 | 9 | 9 | 1 | 3 | 4 | 6 | 6 | 8 | 9 | 0 | 2 | 3 | 4 | 8 | 9 | 9 | 1 | 6 | 7 | 9 | 1 | 2 | 2 | 3 | 3 | 9 | 0 | 2 | 3 | 6 | 3 | 4 | 5 | 9 | 0 | 0 | 1 | 2 | 5 | 7 | 9 | 1 | 2 | 3 | 3 |
|  | 6 | 5 | 1 | 9 | 6 | 0 | 2 | 1 | 4 | 8 | 3 | 8 | 6 | 9 | 4 | 3 | 5 | 6 | 5 | 4 | 3 | 5 | 8 | 9 | 2 | 9 | 7 | 5 | 1 | 7 | 3 | 6 | 3 | 2 | 6 | 5 | 5 | 1 | 6 | 8 | 1 | 4 | 6 | 2 | 5 | 7 | 8 | 3 | 4 | 3 | 2 | 8 |
| **WN-NY99** | **C** | **T** | **T** | **G** | **T** | **C** | **C** | **A** | **G** | **A** | **C** | **G** | **C** | **T** | **C** | **T** | **A** | **C** | **G** | **C** | **C** | **A** | **A** | **C** | **C** | **G** | **A** | **T** | **C** | **C** | **G** | **G** | **C** | **C** | **T** | **A** | **A** | **C** | **A** | **G** | **C** | **C** | **C** | **G** | **G** | **A** | **C** | **C** | **T** | **A** | **A** | **T** |
| BSL53 | T | C | C | . | . | . | . | . | . | . | T | . | . | C | . | . | . | . | . | . | . | . | . | . | . | . | . | . | . | . | . | . | . | . | C | . | . | . | G | . | . | . | . | . | . | . | . | . | . | . | . | C |
| BSL178 | . | . | . | . | . | . | . | . | . | . | . | . | . | . | T | . | G | . | . | . | . | . | . | . | . | . | . | C | . | . | . | . | . | . | . | . | . | . | . | A | . | . | . | . | . | . | . | . | . | . | . | C |
| BSL78 | T | C | . | . | . | T | T | . | . | G | . | . | . | . | . | . | . | . | A | T | . | . | . | . | . | . | . | . | . | . | . | A | . | . | . | . | . | . | . | . | . | . | . | . | . | . | T | . | . | . | . | C |
| BSL85 | T | C | . | A | . | . | . | . | . | . | . | A | . | . | . | . | . | T | . | . | . | . | . | T | T | A | . | . | . | . | . | A | T | . | . | . | . | . | . | . | . | . | . | . | . | . | . | . | . | . | . | C |
| BSL05 | T | C | . | . | . | . | . | . | . | . | . | . | . | . | . | . | . | . | . | . | T | . | . | . | . | . | . | . | . | . | . | . | . | . | . | G | . | . | . | . | . | T | . | . | . | . | . | . | . | . | . | C |
| BSL80 | T | C | . | . | . | . | . | . | . | . | . | . | . | . | . | . | . | . | . | . | T | . | . | . | . | . | . | . | . | . | . | . | . | . | . | G | . | . | . | . | . | T | T | . | . | . | . | . | . | . | . | C |
| BSL93 | T | C | . | . | . | . | . | . | . | . | . | . | . | . | . | . | . | . | . | . | . | C | . | T | . | . | . | . | . | T | . | . | . | T | . | G | . | T | . | . | . | . | . | A | . | . | . | T | . | . | . | C |
| BSL101 | T | C | . | . | . | . | . | . | . | . | . | . | . | . | . | . | . | . | . | . | . | C | . | T | . | . | . | . | . | T | . | . | . | T | . | G | . | T | . | . | . | . | . | A | . | . | . | T | . | . | . | C |
| BSL107 | T | C | . | . | . | . | . | . | . | . | . | . | . | . | . | . | . | . | . | . | . | C | G | T | . | . | . | . | . | T | . | . | . | T | . | G | G | T | . | . | . | . | . | A | . | . | . | T | C | . | . | C |
| BSL116 | T | C | . | . | . | . | . | . | . | . | . | . | . | . | . | . | . | . | . | . | . | C | . | T | . | . | . | . | . | T | . | . | . | T | . | G | . | T | . | . | . | . | . | A | . | . | . | T | . | . | . | C |
| BSL221 | T | C | . | . | . | . | . | . | . | . | . | . | . | . | . | . | . | . | . | . | . | C | . | T | . | . | . | . | . | T | . | . | . | T | . | G | . | T | . | . | T | . | . | A | . | . | . | T | . | . | . | C |
| BSL08 | T | C | . | . | . | . | . | . | A | . | . | . | . | . | . | . | . | . | . | . | . | C | . | T | . | . | . | . | . | T | . | . | . | T | . | G | . | T | . | . | . | . | . | A | . | . | . | T | . | . | . | C |
| BSL140 | T | C | . | . | . | . | . | . | . | . | . | . | T | . | . | C | . | . | . | . | . | C | . | T | . | . | . | . | . | T | . | . | . | T | . | G | . | . | . | . | . | . | . | . | . | . | . | . | . | . | . | . |
| BSL195 | T | C | . | . | C | . | . | . | . | . | . | . | . | . | . | . | . | . | . | . | T | C | . | T | . | . | . | . | . | . | . | . | . | T | . | . | . | . | . | . | . | . | . | . | A | . | T | . | . | . | G | C |
| ARC1 | T | C | . | . | . | . | . | . | . | . | . | . | . | . | . | . | . | . | . | . | . | C | . | T | . | . | . | . | . | T | . | . | . | T | . | G | . | T | . | . | . | . | . | A | . | . | . | T | . | . | . | C |
| ARC3 | T | C | . | . | . | . | . | . | . | . | T | . | . | . | . | . | . | . | . | . | . | C | . | T | . | . | T | . | . | . | A | . | . | T | . | . | . | . | . | . | . | . | . | . | A | . | . | . | . | . | G | C |
| ARC4 | T | C | . | . | . | . | . | G | . | . | . | . | . | . | . | . | . | . | . | . | . | C | . | T | . | . | . | . | . | T | . | . | . | T | . | G | . | T | . | . | . | T | . | A | . | G | . | T | . | . | . | C |
| ARC6 | T | C | . | . | . | . | . | . | . | . | . | . | . | . | . | . | . | . | . | . | . | C | . | T | . | . | . | . | . | T | . | . | . | T | . | G | . | T | . | . | . | . | . | A | . | . | . | T | . | G | . | C |
| ARC13 | . | . | . | . | . | . | . | . | . | . | . | . | . | . | . | . | . | . | . | . | . | . | . | . | . | . | . | . | T | T | . | . | . | T | . | G | . | T | . | . | . | . | . | A | . | . | . | T | . | . | . | C |
|  |  |  |  |  |  |  |  |  |  |  |  |  |  |  |  |  |  |  |  |  |  |  |  |  |  |  |  |  |  |  |  |  |  |  |  |  |  |  |  |  |  |  |  |  |  |  |  |  |  |  |  |  |
| Gene | NS5 | | | | | | | | | | | | | | | | | | | | | | | | | | | | | | | | | | | | | | | | | | | | | | | | | | | |
| Isolate/nt# | 8 | 8 | 8 | 8 | 8 | 8 | 8 | 8 | 8 | 8 | 8 | 8 | 8 | 8 | 8 | 8 | 8 | 8 | 8 | 8 | 8 | 8 | 8 | 8 | 8 | 8 | 8 | 8 | 8 | 8 | 8 | 8 | 8 | 8 | 8 | 8 | 8 | 8 | 8 | 8 | 8 | 8 | 8 | 8 | 8 | 8 | 8 | 8 | 8 | 8 | 9 | 9 |
|  | 0 | 0 | 0 | 1 | 1 | 2 | 2 | 2 | 2 | 2 | 3 | 3 | 3 | 3 | 3 | 4 | 4 | 4 | 4 | 4 | 4 | 4 | 4 | 5 | 5 | 5 | 5 | 5 | 5 | 5 | 5 | 6 | 6 | 6 | 6 | 7 | 7 | 7 | 7 | 8 | 8 | 8 | 8 | 8 | 8 | 8 | 9 | 9 | 9 | 9 | 0 | 0 |
|  | 8 | 8 | 9 | 8 | 9 | 0 | 1 | 2 | 3 | 5 | 2 | 4 | 4 | 6 | 6 | 0 | 2 | 4 | 8 | 8 | 9 | 9 | 9 | 0 | 1 | 1 | 5 | 5 | 5 | 6 | 8 | 0 | 2 | 2 | 6 | 1 | 3 | 7 | 7 | 1 | 1 | 2 | 3 | 4 | 8 | 9 | 1 | 1 | 7 | 8 | 1 | 8 |
|  | 5 | 8 | 7 | 4 | 9 | 5 | 0 | 0 | 2 | 3 | 5 | 6 | 9 | 1 | 5 | 1 | 0 | 2 | 1 | 4 | 1 | 3 | 6 | 5 | 1 | 9 | 0 | 5 | 9 | 5 | 0 | 7 | 1 | 2 | 1 | 2 | 3 | 5 | 8 | 1 | 5 | 3 | 9 | 4 | 3 | 2 | 1 | 9 | 6 | 5 | 5 | 1 |
| **WN-NY99** | **A** | **T** | **T** | **T** | **C** | **G** | **G** | **C** | **C** | **C** | **C** | **G** | **T** | **C** | **G** | **C** | **G** | **C** | **G** | **A** | **C** | **G** | **C** | **C** | **T** | **A** | **C** | **C** | **G** | **C** | **C** | **C** | **A** | **G** | **C** | **C** | **C** | **T** | **T** | **T** | **A** | **C** | **T** | **C** | **C** | **G** | **T** | **C** | **A** | **G** | **G** | **C** |
| BSL53 | . | . | . | . | . | . | . | . | . | . | . | . | . | . | . | T | A | T | . | . | . | . | . | . | . | . | T | . | . | . | . | . | G | . | . | . | . | . | . | C | . | . | . | . | . | . | . | . | . | . | . | . |
| BSL178 | . | . | . | . | . | . | . | . | . | . | . | . | . | . | . | . | . | . | . | . | . | . | . | . | . | . | . | . | . | . | . | . | . | . | T | . | . | . | . | C | . | . | . | . | . | . | . | . | . | . | . | . |
| BSL78 | . | C | . | . | . | . | . | . | . | A | . | A | . | . | . | . | . | . | . | G | . | . | . | . | . | . | . | . | . | T | . | . | . | T | . | . | . | C | . | C | . | . | C | T | . | . | G | . | G | . | . | . |
| BSL85 | . | . | . | C | . | . | . | . | . | . | . | . | . | . | A | . | . | . | . | . | . | . | . | . | . | . | . | . | . | . | T | . | . | . | . | . | . | . | . | C | G | . | . | . | T | . | . | . | . | . | . | . |
| BSL05 | G | . | C | . | . | C | . | . | . | . | . | . | C | . | . | . | . | . | . | . | . | . | . | T | . | . | . | . | A | . | . | . | G | . | . | T | . | . | . | C | . | T | . | . | . | A | . | T | . | . | A | . |
| BSL80 | G | . | C | . | . | C | . | . | . | . | . | . | C | . | . | . | . | . | . | . | . | . | . | T | . | . | . | . | A | . | . | . | G | . | . | T | . | . | . | C | . | T | . | . | . | . | . | T | . | . | A | . |
| BSL93 | . | . | . | . | . | . | . | . | . | . | . | . | . | A | . | . | . | . | . | . | T | . | . | . | . | . | . | . | . | T | . | T | . | . | . | . | . | . | A | C | . | . | . | . | . | . | C | . | . | A | . | . |
| BSL101 | . | . | . | . | . | . | . | . | . | . | . | . | . | A | . | . | . | . | . | . | T | . | T | . | C | . | . | . | . | T | . | T | . | . | . | . | . | . | A | C | . | . | . | . | . | . | . | . | . | A | . | . |
| BSL107 | . | . | . | . | . | . | . | . | . | . | . | . | . | A | . | . | . | . | . | . | T | . | . | . | . | . | . | . | . | T | . | T | . | . | . | . | . | . | A | C | . | . | . | . | . | . | . | . | . | A | . | . |
| BSL116 | . | . | . | . | . | . | . | T | T | . | . | . | . | A | . | . | . | . | . | . | T | A | . | . | . | G | . | . | . | T | . | T | . | . | . | . | . | . | A | C | . | . | . | . | . | . | . | . | . | A | . | . |
| BSL221 | . | . | . | . | . | . | . | . | . | . | . | . | . | A | . | . | . | . | . | . | T | . | . | . | . | . | . | . | . | T | . | T | . | . | . | . | . | . | A | C | . | . | . | . | . | . | . | . | . | A | . | . |
| BSL08 | . | . | . | . | . | . | . | . | . | . | A | . | . | A | . | . | . | . | . | . | T | . | T | . | . | . | . | . | . | T | . | T | . | . | . | . | . | . | A | C | . | . | . | . | . | . | . | . | . | A | . | T |
| BSL140 | . | . | . | . | . | . | . | . | . | . | . | . | . | . | . | . | . | . | . | . | . | . | . | . | . | . | . | . | . | . | . | T | G | . | . | . | . | . | . | . | . | . | . | . | . | . | . | . | . | A | . | . |
| BSL195 | . | . | . | . | . | . | . | . | . | . | . | . | . | . | . | . | . | . | . | . | T | . | . | . | . | . | . | . | . | . | . | T | . | . | . | . | . | . | A | C | . | . | . | . | . | . | . | . | . | . | . | . |
| ARC1 | . | . | . | . | . | . | A | . | . | . | . | . | . | A | . | . | . | . | A | . | T | . | . | . | . | . | . | . | . | T | . | T | . | . | . | . | . | . | A | C | . | . | . | . | . | . | . | . | . | A | . | . |
| ARC3 | . | . | . | . | T | . | . | . | . | . | . | . | . | . | . | . | . | . | . | . | T | . | . | . | . | . | . | . | . | . | . | T | . | . | . | . | T | . | A | C | . | . | . | . | . | . | . | . | . | . | . | . |
| ARC4 | . | . | . | . | . | . | . | . | . | . | . | . | . | . | . | . | . | . | . | . | T | . | . | . | . | . | . | . | . | T | . | T | . | . | . | . | . | . | A | C | . | . | . | . | . | . | . | . | . | A | . | . |
| ARC6 | . | . | . | . | . | . | . | . | . | . | . | . | . | A | . | . | . | . | . | . | T | . | . | . | . | . | . | . | . | T | . | T | . | . | . | . | . | . | A | C | . | . | . | . | . | . | . | . | . | A | . | . |
| ARC13 | . | . | . | . | . | . | . | . | . | . | . | . | . | A | . | . | . | . | . | . | . | . | . | . | . | . | . | T | . | T | . | T | . | . | . | . | . | . | A | C | . | . | . | . | . | . | . | . | . | A | . | . |
|  |  |  |  |  |  |  |  |  |  |  |  |  |  |  |  |  |  |  |  |  |  |  |  |  |  |  |  |  |  |  |  |  |  |  |  |  |  |  |  |  |  |  |  |  |  |  |  |  |  |  |  |  |
| Gene | NS5 | | | | | | | | | | | | | | | | | | | | | | | | | | | | | | | | | | | | | | | | | | | | | | | | | | | |
| Isolate/nt# |  |  |  |  |  |  |  |  |  |  |  |  |  |  |  |  |  |  |  |  |  |  |  |  |  |  |  |  |  |  |  |  |  |  |  |  |  |  |  |  |  |  |  |  |  |  |  |  | 1 | 1 | 1 | 1 |
|  | 9 | 9 | 9 | 9 | 9 | 9 | 9 | 9 | 9 | 9 | 9 | 9 | 9 | 9 | 9 | 9 | 9 | 9 | 9 | 9 | 9 | 9 | 9 | 9 | 9 | 9 | 9 | 9 | 9 | 9 | 9 | 9 | 9 | 9 | 9 | 9 | 9 | 9 | 9 | 9 | 9 | 9 | 9 | 9 | 9 | 9 | 9 | 9 | 0 | 0 | 0 | 0 |
|  | 0 | 1 | 1 | 1 | 1 | 2 | 2 | 2 | 2 | 2 | 2 | 2 | 2 | 2 | 2 | 3 | 3 | 3 | 3 | 3 | 4 | 4 | 4 | 4 | 4 | 5 | 6 | 6 | 6 | 6 | 6 | 7 | 7 | 7 | 7 | 7 | 8 | 8 | 8 | 8 | 9 | 9 | 9 | 9 | 9 | 9 | 9 | 9 | 0 | 0 | 1 | 2 |
|  | 9 | 0 | 3 | 4 | 6 | 2 | 3 | 3 | 4 | 5 | 5 | 6 | 6 | 6 | 9 | 1 | 3 | 4 | 5 | 8 | 1 | 2 | 5 | 6 | 7 | 0 | 0 | 1 | 4 | 6 | 8 | 1 | 2 | 2 | 3 | 5 | 1 | 3 | 3 | 6 | 0 | 0 | 2 | 2 | 5 | 8 | 8 | 9 | 5 | 6 | 8 | 0 |
|  | 0 | 5 | 6 | 7 | 5 | 5 | 6 | 7 | 1 | 5 | 6 | 1 | 2 | 4 | 4 | 2 | 6 | 2 | 2 | 1 | 4 | 6 | 3 | 8 | 1 | 4 | 3 | 2 | 5 | 6 | 7 | 1 | 3 | 9 | 2 | 9 | 9 | 1 | 4 | 5 | 8 | 9 | 6 | 7 | 5 | 7 | 8 | 6 | 9 | 2 | 2 | 6 |
| **WN-NY99** | **C** | **C** | **C** | **G** | **T** | **A** | **A** | **C** | **C** | **C** | **A** | **G** | **C** | **T** | **T** | **C** | **A** | **G** | **C** | **C** | **A** | **G** | **C** | **C** | **C** | **C** | **A** | **A** | **T** | **C** | **C** | **T** | **C** | **C** | **C** | **C** | **T** | **T** | **C** | **C** | **C** | **T** | **C** | **C** | **C** | **T** | **C** | **C** | **C** | **T** | **A** | **A** |
| BSL53 | . | . | . | . | . | . | T | . | T | T | . | . | A | A | . | . | T | . | T | . | . | . | . | . | . | . | . | . | . | . | . | . | . | . | . | . | . | . | . | . | . | . | . | . | . | . | . | . | . | C | . | . |
| BSL178 | . | T | . | . | . | G | . | . | . | . | . | . | . | . | . | . | . | . | T | . | . | . | . | T | . | . | . | . | . | . | . | . | . | . | . | . | . | . | . | . | . | . | . | . | . | . | . | . | A | . | . | . |
| BSL78 | . | . | . | . | . | . | . | . | . | . | . | . | . | . | C | . | . | A | T | . | . | . | . | . | . | . | . | . | . | . | . | . | . | . | . | . | . | . | T | . | . | . | T | . | . | . | T | . | . | . | . | . |
| BSL85 | . | . | T | . | . | . | . | . | . | . | . | . | . | . | . | . | . | . | . | . | . | . | . | . | T | T | . | G | . | T | . | C | . | . | . | . | . | . | . | . | . | . | . | . | . | . | . | T | . | . | G | . |
| BSL05 | T | . | T | . | . | . | . | . | . | . | . | . | . | . | . | T | . | . | T | . | G | . | . | . | . | . | . | . | C | . | . | . | . | . | . | T | . | . | . | . | . | . | . | . | . | . | . | . | . | . | . | . |
| BSL80 | T | . | T | . | . | . | . | . | . | . | . | . | . | . | . | T | . | . | T | . | G | A | . | . | . | . | . | . | C | . | . | . | . | . | . | T | . | . | . | . | . | . | . | . | . | . | . | . | . | . | . | . |
| BSL93 | . | . | . | . | C | . | . | . | . | . | G | . | . | . | . | . | . | . | T | T | . | . | . | . | . | . | . | . | . | . | T | . | . | . | . | . | . | . | . | T | . | . | . | . | . | . | . | . | . | . | . | . |
| BSL101 | . | . | . | . | . | . | . | . | . | . | . | T | . | . | . | . | . | . | T | T | . | . | . | . | . | . | . | . | . | . | T | . | . | . | . | . | . | . | . | T | . | . | . | . | . | . | . | . | . | . | . | . |
| BSL107 | . | . | . | . | . | . | . | . | . | . | . | . | . | . | . | . | . | . | T | T | . | . | T | . | . | . | . | . | . | . | T | . | . | . | . | . | . | . | . | . | . | . | . | . | . | . | . | . | . | . | . | . |
| BSL116 | . | . | . | . | . | . | . | . | . | . | . | . | . | . | . | . | . | . | T | T | . | . | . | . | . | . | . | . | . | . | T | . | . | . | . | . | . | . | . | . | . | . | . | . | . | . | . | . | . | . | . | . |
| BSL221 | . | . | . | . | . | . | . | . | . | . | . | . | . | . | . | . | . | . | T | T | . | . | . | . | . | . | . | . | . | . | T | . | . | . | . | . | . | . | . | T | . | . | . | . | . | C | . | . | . | . | . | . |
| BSL08 | . | . | . | A | . | . | . | . | . | . | . | . | . | . | . | . | . | . | T | T | . | . | . | . | . | . | . | . | . | . | T | . | T | . | T | . | . | . | . | . | . | . | . | . | T | . | . | . | . | . | . | . |
| BSL140 | . | . | . | . | . | . | . | . | . | . | . | . | . | . | . | . | . | . | T | T | . | . | . | . | . | . | . | . | . | . | . | . | . | . | . | . | . | . | . | T | . | . | . | . | . | . | . | . | . | . | . | . |
| BSL195 | . | . | . | . | . | . | . | T | . | . | . | . | . | . | . | . | . | . | T | . | . | . | . | . | T | . | . | . | . | . | . | . | . | T | . | . | . | C | . | . | . | C | . | . | . | . | . | . | . | . | . | . |
| ARC1 | . | . | . | . | . | . | . | . | . | . | . | . | . | . | . | . | . | . | T | T | . | . | . | . | . | . | G | . | . | . | T | . | . | . | . | . | . | . | . | . | . | . | . | . | . | . | . | . | . | . | . | . |
| ARC3 | . | . | . | . | . | . | . | T | . | . | . | . | . | . | . | . | . | . | T | . | . | . | . | . | . | . | . | . | . | . | . | . | . | . | . | . | C | C | . | . | . | . | . | . | . | . | . | . | . | . | . | . |
| ARC4 | . | . | . | . | . | . | . | . | . | . | . | . | . | . | . | . | . | . | T | T | . | . | . | . | . | . | . | . | . | . | T | . | . | . | . | . | . | . | . | . | . | . | . | . | . | . | . | . | . | . | . | G |
| ARC6 | . | . | . | . | . | . | . | . | . | . | . | . | . | . | . | . | . | . | T | T | . | . | . | . | . | . | . | . | . | . | T | . | . | . | . | . | . | . | . | . | T | . | . | G | . | . | . | . | . | . | . | . |
| ARC13 | . | . | . | . | . | . | . | . | . | . | . | . | . | . | . | . | . | . | T | T | . | . | . | . | . | . | . | . | . | . | T | . | . | . | . | . | . | . | . | . | . | . | . | . | . | . | . | . | . | . | . | . |
|  |  |  |  |  |  |  |  |  |  |  |  |  |  |  |  |  |  |  |  |  |  |  |  |  |  |  |  |  |  |  |  |  |  |  |  |  |  |  |  |  |  |  |  |  |  |  |  |  |  |  |  |  |
| Gene | NS5 | | | | | | | | | | | 3'UTR | | | | | | | | | | | | | | | | | | | | | | | |  |  |  |  |  |  |  |  |  |  |  |  |  |  |  |  |  |
| Isolate/nt# | 1 | 1 | 1 | 1 | 1 | 1 | 1 | 1 | 1 | 1 | 1 | 1 | 1 | 1 | 1 | 1 | 1 | 1 | 1 | 1 | 1 | 1 | 1 | 1 | 1 | 1 | 1 | 1 | 1 | 1 | 1 | 1 | 1 | 1 | 1 |  |  |  |  |  |  |  |  |  |  |  |  |  |  |  |  |  |
|  | 0 | 0 | 0 | 0 | 0 | 0 | 0 | 0 | 0 | 0 | 0 | 0 | 0 | 0 | 0 | 0 | 0 | 0 | 0 | 0 | 0 | 0 | 0 | 0 | 0 | 0 | 0 | 0 | 0 | 0 | 0 | 0 | 0 | 0 | 0 |  |  |  |  |  |  |  |  |  |  |  |  |  |  |  |  |  |
|  | 2 | 2 | 2 | 2 | 3 | 3 | 3 | 3 | 3 | 3 | 3 | 4 | 4 | 4 | 4 | 4 | 4 | 4 | 4 | 4 | 4 | 5 | 5 | 5 | 5 | 5 | 6 | 6 | 7 | 7 | 7 | 8 | 8 | 8 | 9 |  |  |  |  |  |  |  |  |  |  |  |  |  |  |  |  |  |
|  | 4 | 5 | 8 | 8 | 0 | 1 | 2 | 2 | 3 | 6 | 9 | 0 | 1 | 2 | 3 | 4 | 5 | 5 | 6 | 8 | 8 | 1 | 3 | 4 | 5 | 6 | 2 | 8 | 1 | 7 | 9 | 2 | 3 | 5 | 0 | Total# | | |  |  |  |  |  |  |  |  |  |  |  |  |  |  |
|  | 8 | 8 | 1 | 4 | 5 | 7 | 1 | 3 | 8 | 8 | 3 | 8 | 1 | 5 | 8 | 3 | 8 | 9 | 1 | 4 | 5 | 6 | 4 | 5 | 0 | 3 | 4 | 8 | 3 | 2 | 5 | 9 | 0 | 1 | 4 |  |  |  |  |  |  |  |  |  |  |  |  |  |  |  |  |  |
| **WN-NY99** | **T** | **G** | **C** | **C** | **C** | **C** | **G** | **T** | **T** | **C** | **C** | **C** | **T** | **T** | **A** | **T** | **G** | **T** | **T** | **T** | **T** | **G** | **A** | **C** | **C** | **A** | **C** | **T** | **A** | **C** | **C** | **T** | **T** | **A** | **C** |  |  |  |  |  |  |  |  |  |  |  |  |  |  |  |  |  |
| BSL53 | . | A | . | . | . | . | . | . | . | . | T | . | . | . | . | . | . | . | . | . | . | . | . | . | . | . | . | . | . | . | . | . | . | G | . | 57 | | |  |  |  |  |  |  |  |  |  |  |  |  |  |  |
| BSL178 | . | . | . | . | . | . | . | . | . | . | . | T | . | . | . | . | . | . | . | . | . | A | . | . | . | . | . | C | . | . | . | . | . | G | . | 61 | | |  |  |  |  |  |  |  |  |  |  |  |  |  |  |
| BSL78 | . | . | . | T | T | . | . | . | . | . | . | . | . | . | . | . | . | . | . | . | . | . | . | . | . | . | . | . | . | . | . | . | . | G | T | 81 | | |  |  |  |  |  |  |  |  |  |  |  |  |  |  |
| BSL85 | . | . | . | . | . | . | . | . | . | . | T | T | . | . | . | . | . | . | . | . | . | . | G | . | . | . | . | . | . | T | . | C | . | G | . | 70 | | |  |  |  |  |  |  |  |  |  |  |  |  |  |  |
| BSL05 | . | . | . | . | . | . | . | C | . | T | T | T | . | . | . | . | . | . | . | . | . | . | . | T | . | . | . | . | . | . | . | . | . | G | . | 81 | | |  |  |  |  |  |  |  |  |  |  |  |  |  |  |
| BSL80 | . | . | . | . | . | . | . | C | . | T | T | T | . | . | . | . | . | . | . | . | . | . | . | . | . | . | . | . | . | . | . | . | . | G | . | 83 | | |  |  |  |  |  |  |  |  |  |  |  |  |  |  |
| BSL93 | C | . | . | . | . | T | . | . | . | . | T | . | . | . | . | C | . | . | . | . | . | . | . | . | . | . | . | . | . | . | . | . | . | G | . | 67 | | |  |  |  |  |  |  |  |  |  |  |  |  |  |  |
| BSL101 | . | . | . | . | . | T | . | . | . | . | . | . | . | . | . | . | . | . | . | . | . | . | . | . | . | . | T | . | . | . | . | . | . | G | . | 65 | | |  |  |  |  |  |  |  |  |  |  |  |  |  |  |
| BSL107 | C | . | . | . | . | T | . | . | . | . | . | . | . | . | . | . | . | . | . | . | . | . | . | . | . | . | . | . | . | . | . | . | . | G | . | 63 | | |  |  |  |  |  |  |  |  |  |  |  |  |  |  |
| BSL116 | C | . | . | . | . | T | . | . | . | . | . | . | . | . | . | . | . | . | . | . | . | . | . | . | . | . | . | . | . | . | . | . | . | G | . | 64 | | |  |  |  |  |  |  |  |  |  |  |  |  |  |  |
| BSL221 | C | . | . | . | . | T | A | . | . | . | . | . | C | . | . | . | . | . | . | . | . | . | . | . | . | . | . | . | G | . | . | . | . | G | . | 69 | | |  |  |  |  |  |  |  |  |  |  |  |  |  |  |
| BSL08 | . | . | . | . | . | . | . | . | . | . | . | . | . | . | . | . | . | . | . | . | . | . | . | . | . | . | . | . | . | . | . | . | . | G | . | 64 | | |  |  |  |  |  |  |  |  |  |  |  |  |  |  |
| BSL140 | C | . | . | . | . | T | . | . | . | . | . | . | . | . | . | . | . | . | . | . | . | . | . | . | . | G | . | . | . | . | . | . | . | G | . | 54 | | |  |  |  |  |  |  |  |  |  |  |  |  |  |  |
| BSL195 | C | . | . | . | . | . | . | . | . | . | . | . | . | . | G | . | . | . | C | G | . | . | . | . | . | . | . | . | . | . | . | . | . | G | . | 69 | | |  |  |  |  |  |  |  |  |  |  |  |  |  |  |
| ARC1 | C | . | . | . | . | T | . | . | . | . | . | . | . | . | . | . | T | C | . | . | . | . | . | . | . | . | . | . | . | . | . | . | . | G | . | 63 | | |  |  |  |  |  |  |  |  |  |  |  |  |  |  |
| ARC3 | C | . | . | . | . | . | . | . | C | . | . | . | . | . | G | . | . | . | C | G | C | . | . | . | . | . | . | . | . | . | . | . | . | G | . | 73 | | |  |  |  |  |  |  |  |  |  |  |  |  |  |  |
| ARC4 | C | . | . | . | . | T | . | . | . | . | T | T | . | C | . | . | . | . | . | . | . | . | . | . | . | . | . | . | . | . | . | . | C | G | . | 63 | | |  |  |  |  |  |  |  |  |  |  |  |  |  |  |
| ARC6 | C | . | T | . | . | T | . | . | . | . | . | T | . | . | . | . | . | . | . | . | . | . | . | . | . | . | T | . | . | . | . | . | . | G | . | 66 | | |  |  |  |  |  |  |  |  |  |  |  |  |  |  |
| ARC13 | C | . | . | . | . | T | . | . | . | . | . | . | . | . | . | . | . | C | . | . | . | . | . | . | T | . | . | . | . | . | T | . | . | G | . | 57 | | |  |  |  |  |  |  |  |  |  |  |  |  |  |  |
