# Supplementary material for: Genetic Variability of West Nile Virus in U.S. Blood Donors from the 2012 Epidemic Season
Source: PLoS Negl Trop Dis. 2016 May 16;10(5):e0004717. doi: 10.1371/journal.pntd.0004717 (PMC4868353; doi:10.1371/journal.pntd.0004717)
Supplement: S3 Table — (DOCX) [file pntd.0004717.s003.docx]

Supplemental Table 3. Amino acid substitutions present in 2012 human WNV isolates, compared to the prototype strain WN-NY99 (AF196835).

| Protein | Core | | | | | prM | | M | | Env | | | | NS1 | | | | | | | NS2A | | | | | | | | | NS2B | NS3 | | |
| --- | --- | --- | --- | --- | --- | --- | --- | --- | --- | --- | --- | --- | --- | --- | --- | --- | --- | --- | --- | --- | --- | --- | --- | --- | --- | --- | --- | --- | --- | --- | --- | --- | --- |
| isolate/aa# | 9 | 36 | 104 | 111 | 113 | 133 | 143 | 248 | 263 | 363 | 449 | 491 | 732 | 885 | 912 | 996 | 1025 | 1027 | 1084 | 1089 | 1166 | 1201 | 1238 | 1262 | 1282 | 1331 | 1333 | 1343 | 1359 | 1494 | 1667 | 1754 | 1839 |
| **WN-NY99** | **G** | **S** | **K** | **I** | **V** | **V** | **T** | **E** | **V** | **T** | **V** | **Y** | **V** | **E** | **S** | **R** | **D** | **I** | **H** | **T** | **V** | **V** | **L** | **H** | **A** | **R** | **K** | **A** | **M** | **V** | **I** | **P** | **S** |
| BSL53 | . | . | . | . | . | . | . | . | A | . | A | . | . | . | . | . | E | . | R | . | . | . | . | . | . | . | R | . | . | . | . | . | . |
| BSL178 | D | . | . | . | . | . | . | D | . | A | A | . | . | . | . | . | . | . | . | . | . | . | F | . | . | . | . | . | . | . | . | . | T |
| BSL78 | . | . | . | . | . | . | . | . | . | . | A | . | I | D | . | . | . | . | . | . | . | . | . | Y | . | . | . | . | . | . | . | . | . |
| BSL85 | . | . | . | . | . | M | . | . | . | . | A | . | . | . | . | . | . | . | . | . | . | . | . | . | . | . | . | S | . | . | M | . | . |
| BSL05 | . | . | . | . | . | . | . | . | . | . | A | . | . | . | . | . | . | . | . | . | . | . | . | . | . | . | . | . | . | . | . | . | . |
| BSL80 | . | . | . | . | . | . | . | . | . | . | A | . | . | . | . | . | . | . | . | . | . | . | . | . | . | . | . | . | . | . | . | . | . |
| BSL93 | . | . | . | . | . | . | . | . | . | . | A | . | . | . | . | . | . | . | . | . | . | I | . | . | . | K | . | . | . | . | . | . | . |
| BSL101 | . | . | . | . | . | . | . | . | . | . | A | . | . | . | . | . | . | . | . | . | . | I | . | . | . | K | . | . | . | . | . | . | . |
| BSL107 | . | . | . | . | . | . | . | . | . | . | A | . | . | . | . | . | . | . | . | . | I | I | . | . | . | K | . | . | . | . | . | . | . |
| BSL116 | . | G | . | . | . | . | . | . | . | . | A | . | . | . | . | . | . | . | . | I | . | . | . | . | . | K | . | . | . | . | . | . | . |
| BSL221 | . | . | . | . | . | . | . | . | . | . | A | . | . | . | . | M | . | . | . | . | . | I | . | . | . | K | . | . | . | . | . | . | . |
| BSL08 | . | . | . | . | . | . | I | . | . | . | A | . | . | . | . | . | . | . | . | . | . | . | . | . | . | K | . | . | . | . | . | . | . |
| BSL140 | . | . | . | . | . | . | . | . | . | . | A | . | . | . | . | . | . | . | . | . | . | I | . | . | . | K | . | . | . | . | . | . | . |
| BSL195 | . | . | R | . | . | . | . | . | . | . | A | H | . | . | . | . | . | V | . | . | . | . | . | . | . | K | . | . | . | I | . | S | . |
| ARC1 | . | . | . | . | . | . | . | . | . | . | A | . | . | . | . | . | . | . | . | . | . | . | . | . | . | K | . | . | . | . | . | . | . |
| ARC3 | . | . | R | T | A | . | . | . | . | . | A | . | . | . | . | . | . | V | . | . | . | . | . | . | . | K | . | . | I | I | . | . | . |
| ARC4 | . | . | . | . | . | . | . | . | . | . | A | . | . | . | . | . | . | . | . | . | . | . | . | . | . | K | . | . | . | . | . | . | . |
| ARC6 | . | . | . | . | . | . | . | . | . | . | A | . | . | . | . | . | . | . | . | . | . | I | . | . | . | K | . | . | . | . | . | . | . |
| ARC13 | . | . | . | . | . | . | . | . | . | . | A | . | . | . | R | . | . | . | . | . | . | . | . | . | T | . | . | . | . | . | . | . | . |
|  |  |  |  |  |  |  |  |  |  |  |  |  |  |  |  |  |  |  |  |  |  |  |  |  |  |  |  |  |  |  |  |  |  |
| Protein | NS3 | | | | NS4A | | | | | NS4B | | | | | NS5 | | | | | | | | | | | | | | | | | |  |
| isolate/aa# | 1860 | 1902 | 2045 | 2087 | 2162 | 2209 | 2213 | 2259 | 2269 | 2287 | 2288 | 2294 | 2347 | 2513 | 2570 | 2577 | 2705 | 2757 | 2775 | 2808 | 2820 | 2842 | 2907 | 2939 | 3047 | 3054 | 3056 | 3080 | 3271 | 3277 | 3388 | 3409 | Total# |
| **NY99** | **Y** | **K** | **T** | **V** | **R** | **A** | **V** | **V** | **S** | **S** | **S** | **I** | **V** | **I** | **H** | **V** | **R** | **V** | **R** | **K** | **S** | **K** | **T** | **L** | **Y** | **T** | **P** | **E** | **A** | **A** | **A** | **D** |  |
| BSL53 | . | . | . | . | K | T | . | . | . | . | . | . | . | . | . | . | . | . | K | . | . | R | . | . | F | . | T | D | . | . | T | . | 13 |
| BSL178 | . | . | . | . | . | . | . | . | . | I | N | T | . | . | . | . | . | . | . | . | . | . | . | . | . | . | . | . | . | . | . | . | 9 |
| BSL78 | . | . | . | . | . | . | . | . | . | . | . | . | . | . | . | . | . | . | . | . | . | N | . | V | . | . | . | . | . | V | . | . | 7 |
| BSL85 | . | . | . | . | . | . | . | . | G | . | R | . | . | . | . | . | . | M | . | . | . | . | A | . | . | . | . | . | . | . | . | . | 8 |
| BSL05 | . | . | . | . | . | . | . | . | . | . | N | . | . | M | Y | . | . | . | . | . | . | R | . | . | . | . | . | . | . | . | . | . | 5 |
| BSL80 | . | . | . | . | . | . | . | . | . | . | N | . | . | M | Y | . | . | . | . | . | . | R | . | . | . | . | . | . | . | . | . | . | 5 |
| BSL93 | . | . | . | I | . | . | . | . | . | . | . | . | . | M | . | . | . | . | . | . | . | . | . | . | . | A | . | . | . | . | . | . | 6 |
| BSL101 | . | . | . | . | . | . | . | M | . | . | . | . | . | M | . | . | . | . | . | . | . | . | . | . | . | . | . | . | . | . | . | . | 5 |
| BSL107 | . | . | . | . | . | . | . | . | . | . | . | . | . | M | . | . | . | . | . | . | . | . | . | . | . | . | . | . | . | . | . | . | 5 |
| BSL116 | . | . | . | . | . | . | . | . | . | . | . | . | . | M | . | . | . | . | . | R | . | . | . | . | . | . | . | . | . | . | . | . | 6 |
| BSL221 | . | . | . | . | . | . | . | . | . | . | . | . | . | M | . | . | . | . | . | . | . | . | . | . | . | . | . | . | . | . | . | N | 6 |
| BSL08 | . | E | . | . | . | . | . | . | . | . | . | . | . | M | . | . | . | . | . | . | . | . | . | . | . | . | . | . | . | . | . | . | 5 |
| BSL140 | . | . | A | . | . | . | . | . | . | . | . | . | . | M | . | . | . | . | . | . | . | R | . | . | . | . | . | . | . | . | . | . | 6 |
| BSL195 | . | . | . | . | . | . | . | . | . | . | . | . | A | . | . | I | . | . | . | . | . | . | . | . | . | . | . | . | . | . | . | . | 9 |
| ARC1 | . | . | . | . | . | . | . | . | . | . | . | . | . | M | . | . | K | . | . | . | . | . | . | . | . | . | . | . | . | . | . | . | 4 |
| ARC3 | . | . | . | . | . | . | A | . | . | . | . | . | . | . | . | I | . | . | . | . | . | . | . | . | . | . | . | . | . | . | . | . | 10 |
| ARC4 | H | . | . | . | . | . | . | . | . | . | . | . | . | M | Y | . | . | . | . | . | . | . | . | . | . | . | . | . | . | . | . | . | 5 |
| ARC6 | . | . | . | . | . | . | . | . | . | . | . | . | . | M | . | . | . | . | . | . | . | . | . | . | . | . | . | . | V | . | . | . | 5 |
| ARC13 | . | . | . | . | . | . | . | . | . | . | . | . | . | M | . | . | . | . | . | . | L | . | . | . | . | . | . | . | . | . | . | . | 5 |
