# Supplementary material for: Genetic Variability of West Nile Virus in U.S. Blood Donors from the 2012 Epidemic Season
Source: PLoS Negl Trop Dis. 2016 May 16;10(5):e0004717. doi: 10.1371/journal.pntd.0004717 (PMC4868353; doi:10.1371/journal.pntd.0004717)
Supplement: S4 Table — (DOCX) [file pntd.0004717.s004.docx]

Supplemental Table 4. Node-specific amino acid substitutions.

| **Node 1** |  |  |  |  |  |  |  |  |  |
| --- | --- | --- | --- | --- | --- | --- | --- | --- | --- |
| **Isolate/Protein** | **E** | **NS2A** |  |  |  |  |  |  |  |
| **AA pp/prot #** | **449/159** | **1262/119** |  |  |  |  |  |  |  |
| **AF196835 1999 WN-NY99** | **V** | **H** |  |  |  |  |  |  |  |
| KJ501302 2002 USA | A | . |  |  |  |  |  |  |  |
| KJ501382 2003 USA | A | . |  |  |  |  |  |  |  |
| HM488200 2007 NY | A | Y |  |  |  |  |  |  |  |
| KM012173 2012 SD | A | Y |  |  |  |  |  |  |  |
|  |  |  |  |  |  |  |  |  |  |
| **Node 2** |  |  |  |  |  |  |  |  |  |
| **Isolate/Protein** | **E** | **NS4B** | **NS5** | |  |  |  |  |  |
| **AA pp/prot #** | **449/159** | **2513/240** | **2570/42** | **2842/314** |  |  |  |  |  |
| **AF196835 1999 WN-NY99** | **V** | **I** | **H** | **K** |  |  |  |  |  |
| JF920752 2008 CT | A | . | Y | . |  |  |  |  |  |
| JF957185 2010 AZ | A | M | Y | R |  |  |  |  |  |
| JF957186 2010 AZ | A | M | Y | . |  |  |  |  |  |
| JQ700440 2011 AZ | A | M | Y | R |  |  |  |  |  |
| KF704158 2010 AZ | A | M | Y | R |  |  |  |  |  |
| KJ145827 2010 NY | A | . | Y | . |  |  |  |  |  |
| KJ501438 2010 USA | A | M | Y | . |  |  |  |  |  |
| KJ501442 2010 USA | A | M | Y | . |  |  |  |  |  |
| KM012170 2012 AZ | A | M | Y | R |  |  |  |  |  |
| KM012174 2012 AZ | A | M | Y | R |  |  |  |  |  |
|  |  |  |  |  |  |  |  |  |  |
| **Node 3** |  |  |  |  |  |  |  |  |  |
| **Isolate/Protein** | **Core** | **E** | | | **NS1** | **NS2A** | | **NS3** | **NS4B** |
| **AA pp/prot #** | **119/119** | **379/89** | **413/123** | **449/159** | **1099/308** | **1195/52** | **1238/95** | **1839/334** | **2287/14** |
| **AF196835 1999 WN-NY99** | **A** | **A** | **T** | **V** | **I** | **T** | **L** | **S** | **S** |
| HM756660 2008 NY | . | . | . | A | . | . | . | . | . |
| JF920754 2008 CT | . | . | . | A | . | I | F | T | I |
| JF488095 2009 NY | . | . | . | A | . | I | F | T | I |
| JF920759 2009 CT | . | . | . | A | . | I | F | T | I |
| KJ501533 2012 NY | . | . | . | A | . | I | F | T | I |
| KM012181 2012 LA | . | . | . | A | . | . | F | T | I |
| KC333376 2012 TX | . | . | . | A | . | I | F | T | I |
| KC333378 2012 TX | . | . | . | A | . | I | F | T | I |
| KC333381 2012 TX | S | . | N | A | V | I | F | T | I |
| KC711058 2012 TX | S | . | N | A | V | I | F | T | I |
| KC736487 2012 TX | S | V | N | A | V | I | F | T | I |
| KC736488 2012 TX | S | V | N | A | V | I | F | T | I |
| KC736489 2012 TX | S | G | N | A | V | I | F | T | I |
| KC736491 2012 TX | S | . | N | A | V | I | F | T | I |
| KC736499 2012 TX | S | V | N | A | V | . | F | T | I |
| KC736501 2012 TX | S | . | N | A | V | I | F | T | I |
| KC736502 2012 TX | S | . | N | A | V | I | F | T | I |
| KJ501216 2012 CO | S | . | N | A | V | I | F | T | I |
| KJ501230 2012 CO | S | . | N | A | V | I | F | T | I |

| **Node 4: 4A in blue,**  **4B in green** | |  |  |  |  |  |  |  |  |  |
| --- | --- | --- | --- | --- | --- | --- | --- | --- | --- | --- |
| **Isolate/Protein** | **C** | **E** | **NS1** | **NS2A** | | **NS2B** | **NS4B** | | **NS5** | |
| **AA pp/prot #** | **104/104** | **449/159** | **1027/236** | **1201/58** | **1331/188** | **1494/120** | **2389/116** | **2513/240** | **2577/49** | **2842/314** |
| **AF196835 1999 WN-NY99** | **K** | **V** | **I** | **V** | **R** | **V** | **T** | **I** | **V** | **K** |
| HM488238 2008 NY | . | A | . | . | K | . | . | . | . | . |
| JF920747 2008 CT | . | A | . | . | K | . | . | . | . | . |
| JQ700439 2011 MS | . | A | . | . | K | . | . | M | . | . |
| KC333384 2012 TX | . | A | . | I | K | . | . | M | . | . |
| KC711059 2012 TX | . | A | . | I | K | . | . | M | . | R |
| KC736486 2012 TX | . | A | . | I | K | . | I | M | . | R |
| KC736490 2012 TX | . | A | . | I | K | . | I | M | . | R |
| KC736492 2012 TX | . | A | . | I | K | . | . | M | . | . |
| KC736493 2012 TX | . | A | . | I | K | . | . | M | . | . |
| KC736494 2012 TX | . | A | . | I | K | . | I | M | . | R |
| KC736495 2012 TX | . | A | . | I | K | . | . | M | . | . |
| KC736496 2012 TX | . | A | . | I | K | . | I | M | . | R |
| KC736497 2012 TX | . | A | . | I | K | . | I | M | . | R |
| KC736498 2012 TX | . | A | . | I | K | . | I | M | . | R |
| KC736500 2012 TX | . | A | . | I | K | . | . | M | . | . |
| KJ501220 2012 CO | . | A | . | I | K | . | . | M | . | . |
| KJ501225 2012 CO | . | A | . | I | K | . | . | M | . | . |
| KJ501226 2012 CO | . | A | . | . | K | . | . | M | . | . |
| KJ501432 2012 USA | . | A | . | . | K | . | . | M | . | . |
| KJ501434 2012 USA | . | A | . | I | K | . | . | M | . | . |
| KJ501530 2012 USA | . | A | . | . | K | . | . | M | . | . |
| KM012171 2012 MS | . | A | . | . | K | . | . | M | . | . |
| KM012176 2012 ND | . | A | . | I | K | . | . | M | . | . |
| KM012177 2012 WY | . | A | . | I | K | . | . | M | . | . |
| KM012178 2012 TX | . | A | . | I | K | . | . | M | . | . |
| KM012179 2012 SD | . | A | . | . | K | . | . | M | . | . |
| KM012180 2012 GA | . | A | . | I | K | . | . | M | . | R |
| KM012183 2012 TX | . | A | . | I | K | . | . | M | . | . |
| KM012184 2012 OH | . | A | . | . | K | . | . | M | . | . |
| KM012186 2012 OH | . | A | . | . | K | . | . | M | . | . |
| KM012187 2012 NE | . | A | . | I | K | . | . | M | . | . |
| KM012188 2012 IL | . | A | . | . | . | . | . | M | . | . |
| KJ501308 2010 USA | R | A | V | . | K | I | . | M | I | . |
| KJ501537 2011 NY | R | A | . | . | K | I | . | . | . | . |
| KC333380 2012 TX | R | A | V | . | K | I | . | . | I | . |
| KC333377 2012 TX | R | A | V | . | K | I | . | . | I | . |
| KC333385 2012 TX | R | A | V | . | K | I | . | . | I | . |
| KJ501224 2012 CO | R | A | V | . | K | I | . | . | I | . |
| KJ501229 2012 CO | R | A | V | . | K | I | . | . | I | Q |
| KJ501437 2012 USA | R | A | V | . | K | I | . | . | I | . |
| KJ501531 2012 NY | R | A | . | . | K | I | . | . | . | . |
| KJ501532 2012 NY | R | A | . | . | . | . | . | . | . | . |
| KJ786936 2012 TX | R | A | V | . | K | I | . | . | I | . |
| KM012182 2012 NM | R | A | V | . | K | I | . | . | I | . |
| KM012185 2012 NE | R | A | V | . | K | I | . | . | I | . |

| **Node 5** |  |  |  |  |
| --- | --- | --- | --- | --- |
| **Isolate/Protein** | **E** | **NS3** | **NS4A** |  |
| **AA pp/prot #** | **449/159** | **1667/162** | **2269/145** |  |
| **AF196835 1999 WN-NY99** | **V** | **I** | **S** |  |
| DQ080059 2003 CA | A | . | G |  |
| KJ501267 2005 USA | A | . | G |  |
| KJ501341 2005 USA | A | . | G |  |
| KJ501195 2007 CA | A | . | G |  |
| KJ501206 2008 CA | A | . | G |  |
| KJ501100 2009 CA | A | M | G |  |
| JQ700441 2011 CA | A | M | . |  |
| KJ501096 2011 CA | A | M | G |  |
| KJ501098 2011 CA | A | M | G |  |
| KM012175 2012 CA | A | M | G |  |
|  |  |  |  |  |
| **Node 6** |  |  |  |  |
| **Isolate/Protein** | **E** | **NS4A** | **NS5** | **NS5** |
| **AA pp/prot #** | **449/159** | **2209/85** | **2842/314** | **3388/860** |
| **AF196835 1999 WN-NY99** | **V** | **A** | **K** | **A** |
| GQ507470 2006 TX | A | T | R | T |
| GQ507471 2007 TX | A | T | R | T |
| JX015516 2007 TX | A | T | R | T |
| KJ501108 2007 CA | A | T | R | T |
| KJ501112 2007 CA | A | T | R | T |
| KJ501113 2008 CA | A | T | R | T |
| KJ501114 2007 CA | A | T | R | T |
| JX015523 2010 TX | A | T | R | T |
| KM012172 2012 TX | A | T | R | T |
