# Supplementary figures and images for: Genetic Variability of West Nile Virus in U.S. Blood Donors from the 2012 Epidemic Season
Source: PLoS Negl Trop Dis. 2016 May 16;10(5):e0004717. doi: 10.1371/journal.pntd.0004717 (PMC4868353; doi:10.1371/journal.pntd.0004717)

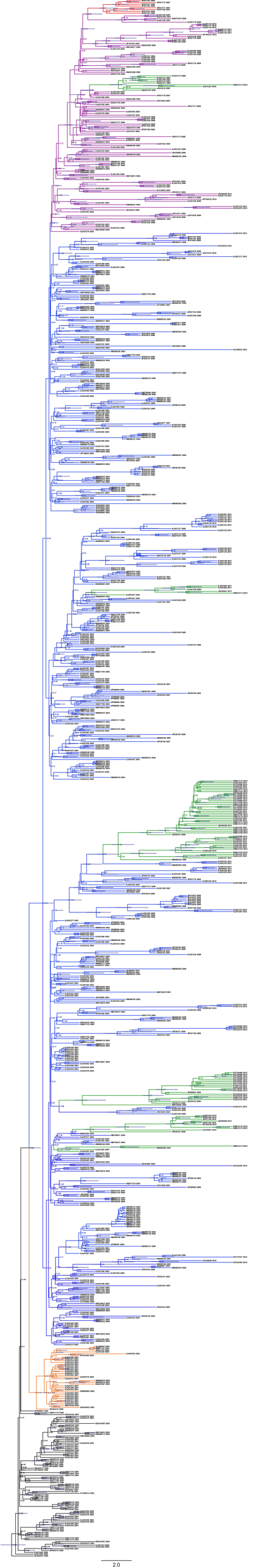

Supplement: S2 Fig — WNV genotypes are color-coded in the branches of the tree as NY99 (black), WN02 (blue), SW/WN03 (purple) and cluster MW/WN06 (red). Nodes 1 to 6 containing WNV isolates from this study are highlighted in green. The mean time to the most recent common ancestor (tMRCA) is shown in each principal node. The 95% highest probability densities (95% HPD) for each node age are shown as blue bars. (TIFF) [file pntd.0004717.s006.tiff]
